# Multivariate genome-wide analysis of aging-related traits identifies novel loci and new drug targets for healthy aging

**DOI:** 10.1038/s43587-023-00455-5

**Published:** 2023-08-07

**Authors:** Daniel B. Rosoff, Lucas A. Mavromatis, Andrew S. Bell, Josephin Wagner, Jeesun Jung, Riccardo E. Marioni, George Davey Smith, Steve Horvath, Falk W. Lohoff

**Affiliations:** 1 Section on Clinical Genomics and Experimental Therapeutics, National Institute on Alcohol Abuse and Alcoholism, National Institutes of Health, Bethesda, MD USA; 2 NIH-Oxford-Cambridge Scholars Program; Radcliffe Department of Medicine, University of Oxford, Oxford, UK; 3 MRC Integrative Epidemiology Unit at the University of Bristol, Bristol, UK; 4 Centre for Genomic and Experimental Medicine, Institute of Genetics and Cancer, University of Edinburgh, Edinburgh, UK; 5 Department of Biostatistics, Fielding School of Public Health, University of California Los Angeles, Los Angeles, CA USA; 6 Department of Human Genetics, David Geffen School of Medicine, University of California Los Angeles, Los Angeles, CA USA; 7 San Diego Institute of Science, Alto Labs, San Diego, CA USA

**Keywords:** Genome-wide association studies, Drug discovery, Ageing

## Abstract

The concept of aging is complex, including many related phenotypes such as healthspan, lifespan, extreme longevity, frailty and epigenetic aging, suggesting shared biological underpinnings; however, aging-related endpoints have been primarily assessed individually. Using data from these traits and multivariate genome-wide association study methods, we modeled their underlying genetic factor (‘mvAge’). mvAge (effective *n* = ~1.9 million participants of European ancestry) identified 52 independent variants in 38 genomic loci. Twenty variants were novel (not reported in input genome-wide association studies). Transcriptomic imputation identified age-relevant genes, including *VEGFA* and *PHB1*. Drug-target Mendelian randomization with metformin target genes showed a beneficial impact on mvAge (*P* value = 8.41 × 10^−5^). Similarly, genetically proxied thiazolidinediones (*P* value = 3.50 × 10^−10^), proprotein convertase subtilisin/kexin 9 inhibition (*P* value = 1.62 × 10^−6^), angiopoietin-like protein 4, beta blockers and calcium channel blockers also had beneficial Mendelian randomization estimates. Extending the drug-target Mendelian randomization framework to 3,947 protein-coding genes prioritized 122 targets. Together, these findings will inform future studies aimed at improving healthy aging.

## Abstract (web summary)

Using a multivariate genome-wide association study approach, the authors identified 52 genetic variants associated with aging-related traits, many of which are promising cardiometabolic targets that could promote healthy aging and inform future interventions.

## Main

While human aging is a multifaceted process influenced by many factors^1,2^ and characterized by reduced maintenance of homeostatic mechanisms, age-related diseases and death^3^, there exists substantial variability in how humans age^3^. Some individuals may be subject to chronic health problems and disease and die early while others may reach old age relatively healthy^3^. Understanding the factors underlying this variation is important for the development of public health interventions and therapeutics to improve healthy aging^4^.

Genome-wide association studies (GWASs) have begun to identify aging-related loci using single-phenotype approaches^5–7^, including extreme longevity^8^, healthspan^9^ and parental lifespan^1^. However, these single-endpoint approaches fail to account for the shared genetics among these traits or other aging-related traits, such as epigenetic age acceleration (EAA)^10^ and frailty^11^, which would provide further insight into the broad genetic architecture underlying how humans age and inform the shifting focus in geroscience from studying survival toward incorporating complementary measures of age-related outcomes^12^ to improve healthy aging—defined as the maintenance of well-being in old age that includes both the absence of disease and the presence of happiness, satisfaction and fulfillment^13^. Further, EAA may be reversible^10^, underscoring the potential value of elucidating mechanisms and discovering targets that slow aging^10^. Recent advances in multivariate GWAS approaches incorporate univariate GWAS summary statistics to facilitate discovery of the genetic architecture underlying related phenotypes^14^. In contrast to single-phenotype GWAS methods, multivariate approaches enhance discovery of novel biological correlates by boosting statistical power through increased effective sample sizes^14^ and have been recently applied to identify genomic loci shared across neuropsychiatric disorders^14^, alcohol consumption behaviors^15^ and externalizing behaviors^16^.

We apply genomic structural equation modeling (genomic SEM)^14^ to summary-level GWASs on healthspan^9^, parental lifespan^1^, extreme longevity^8^, frailty^11^ and epigenetic aging^10^ to construct a multivariate aging-related GWAS (here termed ‘mvAge’) to identify novel genetic variants that broadly impact healthy aging processes. We perform bioannotation—including fine mapping, a transcriptome-wide association study (TWAS) and cell-type enrichment. We also use several applications of Mendelian randomization (MR)^17^ aimed at identifying modifiable risk factors and biomarkers to support healthy aging initiatives. Further, given the importance and interest in repurposing and developing therapeutics to improve healthy aging (for example, ongoing clinical trials evaluating the potential of metformin^18^), and because genetic evidence supporting candidate compounds entering clinical trials increases the probability of clinical success, we use drug-target MR^19^ to investigate potential therapeutic repurposing opportunities among gene targets of metformin, 6 other antidiabetic classes, 15 lipid-lowering therapies and 5 classes of antihypertensive drugs. We also leverage drug-target MR to perform screens of protein-coding genes that will inform future studies investigating potential therapeutic targets to improve healthy aging.

## Results

**Fig. 1:**
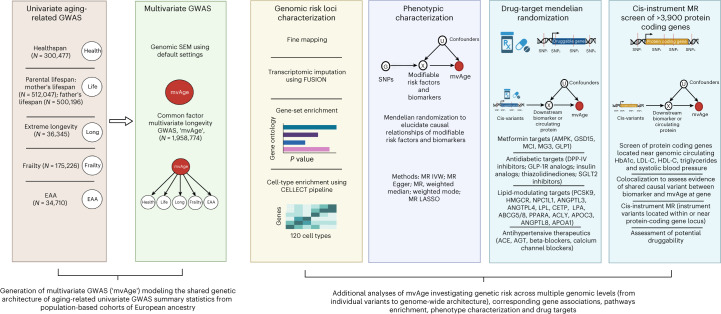
Study overview. An overview of this study’s data sources, analytical flow and methodology. Created with BioRender.com. The univariate input GWASs of frailty and EAA were reverse coded to align their effects to have positive relationships with healthspan, lifespan and extreme longevity. GWAS, genome-wide association study; EAA, epigenetic age acceleration; CELLECT, CELL-type Expression-specific integration for Complex Traits; IVW, inverse variance weighted; MR LASSO, MR Least Absolute Shrinkage and Selection Operator; HbA1c, glycated hemoglobin; LDL-C, low-density lipoprotein cholesterol; HDL-C, high-density lipoprotein cholesterol.

A study overview is presented in Fig. 1.

### Structural equation modeling

**Fig. 2:**
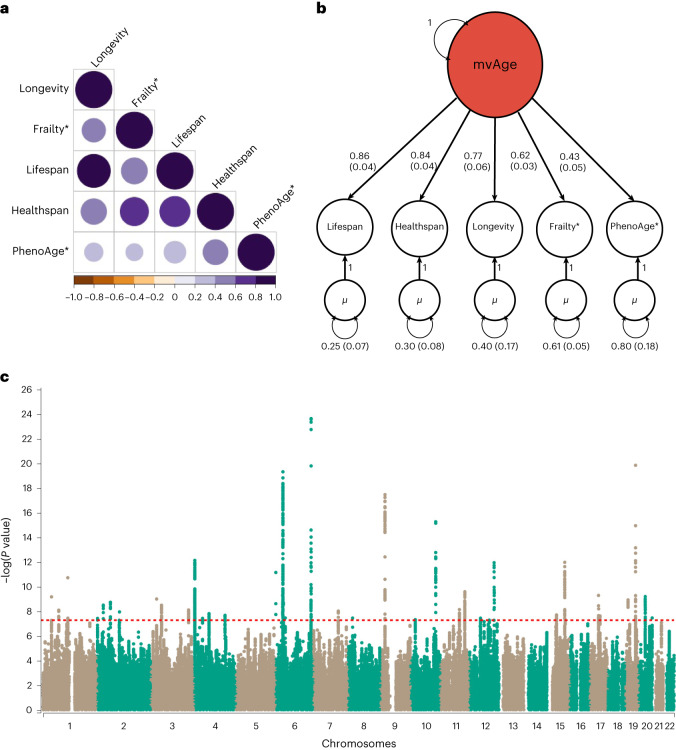
Multivariate aging GWAS modeled with genomic SEM. **a**, Genetic correlations for SEM with genomic SEM, displaying pairwise LD score genetic correlation estimates for the five univariate phenotypes. **b**, Path diagram of the common factor model estimated with genomic SEM, with standardized factor loadings (standard error in parentheses). **c**, Manhattan plot showing SNP associations (−log_10_(*P* value)) with mvAge, ordered by chromosome. The red dashed line indicates the threshold for conventional genome-wide significance (*P* value = 5 × 10^−8^). *P* values are derived from two-sided Wald tests for each SNP on mvAge. * indicates that summary statistics for frailty and PhenoAge (the epigenetic clock variable) were reversed to align with the other longevity-related endpoints. *µ* reflects the residual variance in the genetic indicators for the input univariate age-related GWASs not explained by the mvAge common factor. Source data

Linkage disequilibrium (LD) score regression indicated that the five univariate input GWASs, representing the genetic aging-related liabilities of healthspan, frailty, exceptional longevity, parental lifespan and EAA, were positively correlated (frailty and EAA were reverse coded) (Fig. 2 and Supplementary Tables 1 and 2). We performed SEM in preparation for the multivariate GWAS. The common factor model fit of the implied genetic covariance matrix between the five input GWASs with the empirical covariance matrix was good (comparative fit index (CFI) = 0.97, standardized root mean square residual (SRMR) = 0.069) (Fig. 2 and Supplementary Tables 3 and 4), suggesting evidence for a shared genetic factor mvAge.

### Multivariate GWAS meta-analysis

Expanding the SEM model to incorporate individual variants, we generated a multivariate GWAS estimating 6,793,898 associations at single nucleotide polymorphism (SNP) level (Supplementary Table 5) for our shared aging factor mvAge. Mean *χ*^2^ and λ_GC_ (genomic control) were estimated at 1.43 and 1.52, respectively, and the LD score intercept, 0.997 (s.e. = 0.0098), suggesting inflation due to polygenic heritability signals rather than population stratification bias (Supplementary Fig. 1)^14,20^. Effective sample size was calculated at 1,958,774 using mvAge summary statistics restricted to minor allele frequency (MAF) limits of 10% and 40% to produce stabler estimates^14^. We identified 52 lead SNPs in 38 genomic loci (*P* value < 5 × 10^−8^) (Fig. 2 and Supplementary Table 6). Twenty of the 52 SNPs were novel compared to loci identified in the five input GWASs underlying mvAge (Supplementary Tables 6 and 7 and Supplementary Figs. 2–21), highlighting the increased power of genomic SEM. Novel mvAge lead SNPs were generally enriched for traits, that is, systolic blood pressure (SBP), body mass index (BMI), brain morphology and type 2 diabetes (T2D). For 11 of the 20 novel SNPs, the lead SNP was identified in a previous GWAS from the GWAS Catalog, while the other previous associations were from variants in LD with the lead SNPs; 4 of the 11 novel variants (rs12769128, rs17499404, rs2643826 and rs9277988) were linked with aging-related traits (that is, previous GWASs of specific aging phenotypes like lifespan in the AncestryDNA cohort^21^), while another four (rs114298671, rs1689046, rs78438918 and rs6062322) were not linked with specific aging phenotypes; however, they were associated with important factors that may influence healthy aging (for example, cognition, BMI and hypertension) (Table 1 and Supplementary Tables 8 and 9).

**Table 1:** Novel lead SNPs identified in mvAge

SNP	Location (chr,pos)	EA/OA	MAF	Beta (SE)	*P* value	Nearest mapped gene (SNP function)	SNP *Q* *P* value	Previous GWAS associations (*P* value < 5 × 10^−8^)
rs114298671	4,3281869	G/A	0.12	0.0089 (0.0016)	1.57 × 10^−8^	*MSANTD1* (intergenic)	0.77	Adult body size, BMI
rs12769128	10,21883430	C/T	0.32	0.006 (0.0011)	4.56 × 10^−8^	*MLLT10* (intronic)	0.60	Adult body size, AncestryDNA cohort parental lifespan, BMI, body fat, breast cancer, brain structure, education, ovarian cancer, hypertension, gallstone disease, dietary behavior, GRD, insomnia, lung function, chronic pain, physical activity, smoking behavior, sodium excretion, albumin-to-creatine ratio
rs13141210	4,67891641	C/T	0.50	−0.0058 (0.001)	1.46 × 10^−8^	*RNU6-699P* (intergenic)	0.55	Adult body size, cognitive performance, education, household income, lung cancer
rs1689406	12,89754726	A/G	0.20	0.0069 (0.0013)	4.95 × 10^−8^	*RP11-1109F11.3* (intergenic)	0.24	ADHD
rs17499404	4,38385479	G/A	0.46	−0.0057 (0.001)	3.57 × 10^−8^	*RP11-83C7.1*	0.006	Aging traits, SBP, DBP, use of diuretics, PP
rs2613508	1,72833582	C/T	0.18	0.0077 (0.0013)	7.77 × 10^−9^	*RPL31P12* (intergenic)	0.60	Adult body size, aging traits, BMI, CVD, childhood BMI and obesity, GRD, heart rate response to exercise, insomnia, life satisfaction, neuroticism, extreme obesity, smoking initiation, triglycerides, T2D, waist-to-hip ratio
rs2643826	3,27562988	C/T	0.45	0.0063 (0.001)	9.66 × 10^−10^	AC137675.1 (intergenic)	0.42	Aging traits, CVD, DBP, DBP × alcohol interaction, hypertension, mean arterial pressure × alcohol interaction, antihypertensive use, PP, SBP, SBP × alcohol interaction
rs268	8,19813529	A/G	0.014	0.024 (0.0044)	3.38 × 10^−8^	*LPL* (exonic)	0.87	Apolipoprotein A1, apolipoprotein B, HDL-C, reticulocyte volume and width, statin use, metabolic syndrome, triglycerides
rs28637671	4,67780392	T/G	0.29	0.0063 (0.0011)	1.95 × 10^−8^	*RNU6-699P* (intergenic)	0.24	None
rs36072649	4,140939110	T/A	0.37	−0.006 (0.0011)	2.01 × 10^−8^	*MAML3* (intronic)	0.74	Adult body size, age of first birth, first sexual intercourse age, BMI, personality disorder, CRP, depression, smoking behavior, education, anti-inflammatory medication use, chronic pain, visceral adipose content, T2D, walking pace, waist-to-hip ratio
rs3768321	1,40035928	G/T	0.19	0.0079 (0.0013)	6.46 × 10^−10^	*PABPC4,RP11-69E11* (ncRNA-intronic)	0.02	Apolipoprotein A1, BMI, brain structure, CRP, calcium levels, COPD, DBP, HbA1c, HDL-C, HDL-C/environment interactions, MAP, hemoglobin biology, T2D, T2D medication, liver function enzymes, SHBG, testosterone levels, triglycerides, triglycerides/environment interaction, walking pace, waist-to-hip ratio
rs55686423	12,49963534	A/T	0.09	−0.010	3.45 × 10^−8^	*PRPF40B* (intronic)	0.53	Cognitive performance, smoking, PP, cognitive resilience, SBP
rs6062322	20,62441599	A/T	0.19	−0.0071 (0.0013)	3.30 × 10^−8^	*ZBTB46,RP4-583P15.11* (ncRNA-intronic)	0.38	Birth weight, mean corpuscular volume, antihypertensive use
rs6907508	6,34592090	A/G	0.10	0.010	1.90 × 10^−9^	*C6orf106* (intronic)	0.095	Apolipoprotein A1, apolipoprotein B, basophil count, body fat, BMI, brain structure, CVD, eczema, HDL-C, HDL-C/environment interactions, height, hip circumference, LDL-C, LDL-C/environment interactions, liver enzyme levels, lung function, metabolic syndrome, white blood cell count, chronic pain, baldness, waist-to-hip ratio
rs7174250	15,81018587	C/T	0.46	0.0056 (0.001)	4.82 × 10^−8^	*ABHD17C* (intronic)	0.89	Atrial fibrillation, BMI, CVD, DBP, antihypertensive use, PP, SBP
rs7742789	6,43345803	C/T	0.35	0.006 (0.0011)	3.30 × 10^−8^	*ZNF318* (intergenic)	0.41	Age-related hearing loss, stroke, chronotype, DBP, heel bone density, height, hip circumference, hypertension, testosterone levels, MAP, antihypertensive use, platelets, PP, SBP, uric acid level, waist-to-hip ratio
rs78438918	2,100630115	A/G	0.17	−0.0078 (0.0014)	1.07 × 10^−8^	*AFF3* (intronic)	0.81	Cognitive performance, household income
rs9277988	6,33306235	T/C	0.20	0.0071 (0.0013)	3.59 × 10^−8^	*MYL8P* (upstream)	0.24	Adult body size, BMI, circadian rhythm disruption, education, AncestryDNA cohort parental lifespan, visceral adipose tissue, smoking, walking pace
rs940088	17,47145848	T/C	0.29	−0.0063 (0.0011)	2.27 × 10^−8^	*IGF2BP1* (intergenic)	0.86	Adult body size, AUD, BMI, brain volumes, CVD, cognitive performance, insomnia, AncestryDNA cohort parental lifespan, visceral adipose tissue, walking pace
rs980183	2,59311536	G/A	0.38	−0.0063 (0.0011)	1.76 × 10^−9^	*LINC01122* (intergenic)	0.56	Adult body size, BMI, CRP, HDL-C, visceral adipose tissue, PP, smoking, SBP, triglycerides, T2D, urate levels, waist-to-hip ratio

Lead SNPs were defined as novel if they were >1 Mb from previously identified loci in the univariate aging-related GWASs comprising the mvAge. *Q*_SNP_ heterogeneity statistics (*P* value of *Q*) evaluated whether the multivariate SNP associations are appropriately modeled through a multivariate framework^14^. Because the null hypothesis of the *Q*_SNP_ test is that the SNP associations on the univariate GWASs are statistically mediated by the resultant multivariate GWAS, significant *Q*_SNP_ tests in the multivariate GWAS summary statistics suggest that the SNP impacts the univariate GWASs by pathways other than mvAge (see Methods and Supplementary Methods for additional information). Previous associations of the variants were assessed using the GWAS Catalog and were included if the lead variant, or variants with LD *R*^2^ > 0.6, had *P* values < 5 × 10^−8^. Gene names for the nearest mapped genes are italicized. SNP Cochran’s *P* values for *Q* were derived from two-sided *χ*2 test. *P* values for SNP effects were derived from two-sided Wald tests. ADHD, attention deficit/hyperactivity disorder; AUD, alcohol use disorder; chr, chromosome; COPD, chronic obstructive pulmonary disease; CRP, C-reactive protein; CVD, cardiovascular disease; DBP, diastolic blood pressure; EA, effect allele; GRD, gastroesophageal reflux disease; HbA1c, glycated hemoglobin; HDL-C, high-density lipoprotein cholesterol; LDL-C, low-density lipoprotein cholesterol; MAP, mean arterial pressure; ncRNA, non-coding ribonucleic acid; OA, other allele; pos, genomic position; PP, pulse pressure; SE, standard error of the beta; SHBG, sex hormone binding globulin.

### Fine mapping

Fine-mapping analysis identified strong associations with several loci, including on chromosomes 1 (rs1230666, intronic variant in *MAGI3*); 6 (rs12203592 and rs9277988); 8 (rs268, an exonic variant within *LPL*); 19 (rs7412 in the *APOE* locus); and 20 (rs1737896). Regional plots show clear peaks at these loci with other credible set variants showing evidence of association (Supplementary Figs. 22–33 and Supplementary Table 10).

### *Q*_SNP_ heterogeneity

Evaluating whether the multivariate SNP associations are appropriately modeled through a multivariate framework^22^, 9 of the 52 lead SNPs generated *Q*_SNP_ *P* values < 9.62 × 10^−4^, the Bonferroni-adjusted threshold, suggesting these SNPs impact the input aging-related GWAS endpoint by pathways other than mvAge^22^. However, none of the 20 novel SNPs had *Q*_SNP_ *P* values < 9.62 × 10^−4^ (Supplementary Table 6).

### Transcriptomic imputation

**Extended Data Fig. 1:**
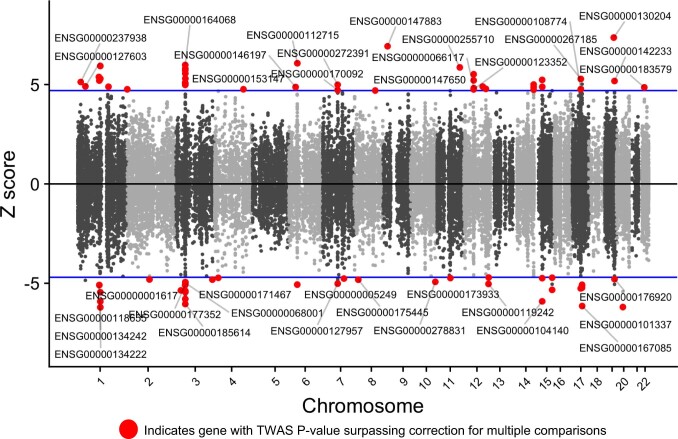
Gene-level Manhattan plot of transcriptome-wide association study. TWAS Z-scores (effect estimates of the imputed gene expression on *mvAge*) are plotted (*mvAge* N = 1,958,774). The expression quantitative trait loci (eQTL) data are derived from the GTEx Version 8 sparse canonical correlation analysis performed by Feng et al. 2021 (see References). The blue lines represent Z = ± 4.69, corresponding to the Bonferroni adjusted P-value threshold = 1.38 × 10^−6^ (0.05/36,149 sCCA features available for analysis). Red points and labels indicate genes (Ensembl gene IDs) surpassing the threshold. TWAS, transcriptome-wide association study; sCCA, sparse canonical correlation analysis. Source data

Next, we performed a TWAS using FUSION^23^ to identify gene-level associations with the mvAge genetic signature. We found 57 genes surpassing correction for multiple comparisons (Extended Data Fig. 1 and Supplementary Table 11). We took these genes forward for further testing, including colocalization^24^ and FOCUS fine mapping for TWAS^25^. Of the 57 TWAS-significant genes, 18 represented colocalized and potentially causal signals with mvAge. These ‘high-confidence’ gene-level associations included *CDKN2A*, *PTPN22*, *PHB1*, and *VEGFA*. TWAS *Z* scores for *CDKN2A* and *VEGFA* were both >0, indicating that predicted gene expression positively associated with mvAge, suggesting upregulation of the genes may be associated with increased mvAge. By contrast, with TWAS *Z* scores <0, results suggest downregulation of *PTPN22* and *PHB1* associated with increased mvAge.

### Exploratory two-factor analysis

Genomic SEM’s exploratory factor analysis (EFA) was used to guide the specification of a more nuanced two-factor model (Supplementary Table 3). Based on the EFA, we ran a follow-up confirmatory factor analysis. A two-factor solution provided good fit to the data (CFI = 0.996, SRMR = 0.035) (Supplementary Table 4), with factor loadings suggesting one latent factor comprised life-expectancy-related GWASs (parental lifespan, extreme longevity and PhenoAge EAA) and the other comprised the healthy-aging-related GWASs (healthspan and frailty). A strong genetic correlation between the two factors (*r*_g _= 0.76, *P* value = 2.4 × 10^−48^) suggests shared but distinct components of life expectancy/lifespan and healthy aging, both captured by mvAge.

### Pathway, cell-type and Mendelian-disease-gene enrichment

Multimarker analysis of genomic annotation (MAGMA)^26^ gene-based mapping found 164 genes (Supplementary Table 12) that we used to perform our gene-set analysis, which were enriched for gene ontology and REACTOME terms (Supplementary Table 13); many of the gene sets were related to lipids (that is, plasma lipoprotein assembly, triglyceride (TG) and very low-density lipoprotein clearance, TG metabolic processes, protein–lipid complex assembly and components of chylomicrons). Cell-type enrichment showed six cell types surpassing correction for multiple comparisons (Supplementary Table 14). The top two cell types were the lymphoid and granulocyte/monocyte progenitor cells. mvAge was enriched primarily in immune cells/immune cell progenitors, with 8 of 13 cell types with *P* values < 0.05 related to immune cells. Tests for enrichment of mvAge in Mendelian disease genes and associated pathways showed six Mendelian diseases, including primary ciliary dyskinesia (Supplementary Table 15), and 21 phenotypic abnormalities, including several gene sets related to respiratory system function (Supplementary Table 16).

### MR with modifiable risk factors and biomarkers

We used MR to assess possible causal relationships of 73 genetically predicted biomarkers and risk factors on mvAge (Supplementary Table 17). Twenty-five of 73 risk factors and biomarkers generated MR estimates surpassing the Bonferroni correction. MR estimates were consistent across complementary MR methods used as sensitivity analyses for the primary inverse variance weighted (IVW) estimate. In addition, MR Lap estimates were consistent with the IVW estimates, suggesting minimal bias due to sample overlap^27^. (Full results are described in Supplementary Results.)

### Metformin target genes impact mvAge

**Fig. 3:**
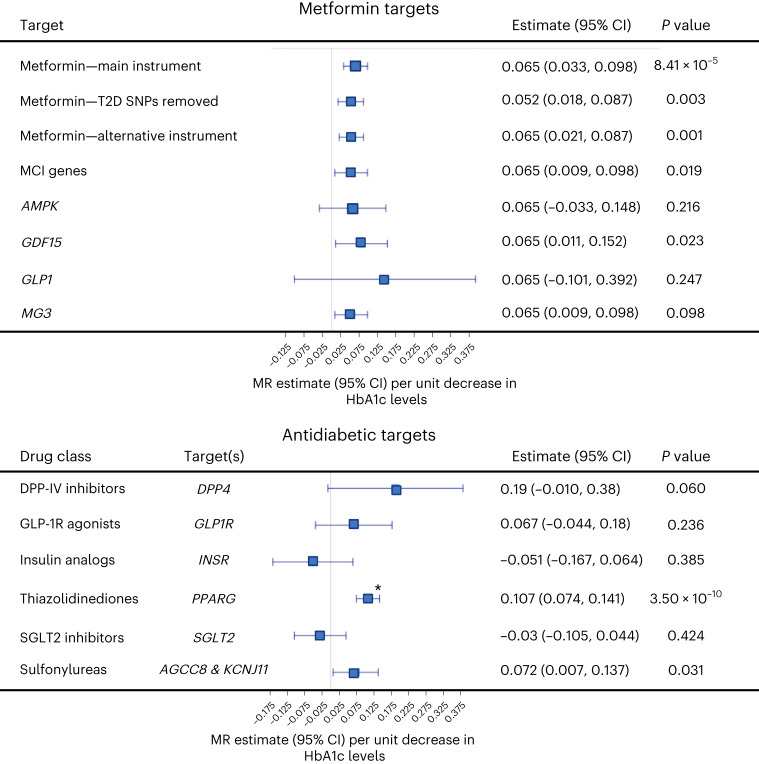
Drug-target MR results assessing proxying metformin and other antidiabetics targets with mvAge. Data presented are MR effect estimates (betas) for the IVW MR method (the primary MR method) and the corresponding 95% confidence intervals (CIs) aligned to proxy the pharmacological effect of metformin and antidiabetic genes (HbA1c GWAS *n* = 344,182) (1 s.d. lowering of HbA1c levels) on mvAge (*n* = 1,958,774). The vertical line in the center of the forest plots is 0, corresponding to no change in the IVW estimate of the drug targets on mvAge. Full results are presented in Supplementary Tables 15 and 16. Metformin results plotted show the MR estimates for the primary metformin instrument (top row), which comprised variants within genes for five metformin targets (*AMPK*, *GSD15*, *MCI*, *MG3* and *GLP1*), for the estimates of the alternative metformin instruments used as sensitivity analyses, and the metformin targets separated into individual instruments. See Methods and Supplementary Methods sections for additional details. For the analyses of the antidiabetic classes not including the metformin targets, the * indicates that the thiazolidinedione MR estimate surpasses the Bonferroni-adjusted *P-*value threshold = 0.002, corrected for the 25 antidiabetic, lipid-modulating and antihypertensive drug targets compared. *P* values are derived from two-sided Wald tests. Gene names for the nearest mapped genes are italicized. Source data

Results are oriented to mimic pharmacological modulation of the drug target, namely, a lowering of HbA1c (s.d. decrease (mmol mol^−1^)) (Fig. 3 and Supplementary Table 18). (Supporting its validity as a genetic proxy for metformin, the primary instrument was associated with reduced risk for T2D (odds ratio = 0.40, *P* value = 2.5 × 10^−4^).) Lowering HbA1c via the metformin target genes linked beneficially with mvAge, which beneficial relationship remained after removing SNPs nominally associated with T2D from the primary instrument as well as in analyses using a second instrument from a recent MR study evaluating the impact of metformin on dementia^28^. Analyses of the univariate aging-related input GWASs suggested beneficial relationships of metformin target genes with healthspan, epigenetic aging and longevity. Finally, MR distinguishing individual metformin gene targets showed relationships of the mitochondrial complex 1 and the GDF15 targets with mvAge.

### Genetic impact of targets for cardiometabolic drug classes

**Fig. 4:**
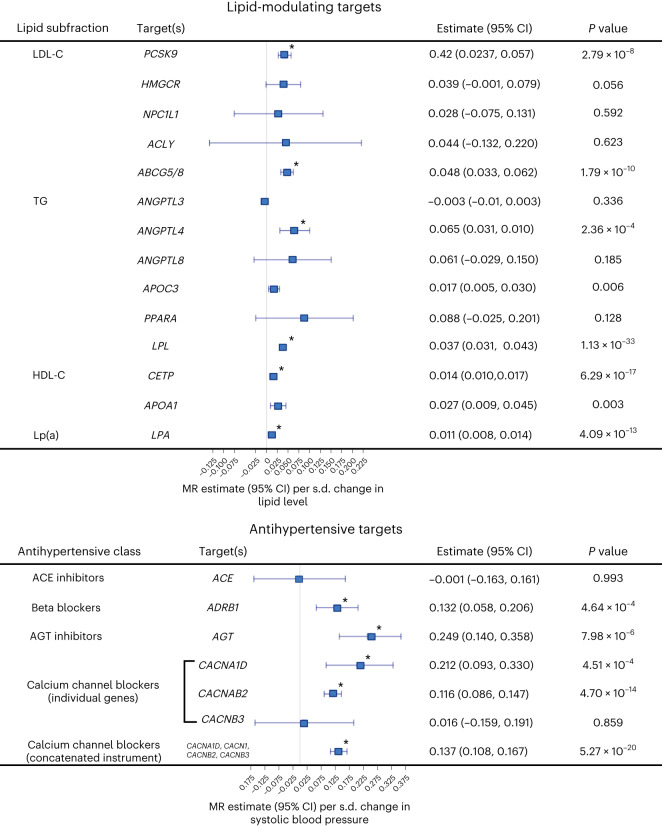
Drug-target MR results assessing the impact of lipid-modulating and antihypertensive gene targets on mvAge. Data presented are MR effect estimates (betas) for the IVW MR method (the primary MR method) and the corresponding 95% CIs aligned to proxy the pharmacological effect of modulated lipid levels (1 s.d. lower LDL-C (*n* = 440,546), 1 s.d. lower TG (*n* = 441,016), and 1 s.d. higher HDL-C (*n* = 403,943)) and SBP (*n* = 436,419) (antihypertensive gene targets (1 s.d. lower SBP) on mvAge (*n* = 1,958,774)). The vertical line in the center of the forest plots is 0, corresponding to no change in the IVW estimate of the drug targets on mvAge. Full results are presented in Supplementary Table 17. * indicates a *P* value surpassing the Bonferroni-adjusted *P-*value threshold = 0.002, corrected for the 25 antidiabetic, lipid-modulating and antihypertensive drug targets compared. *P* values are derived from two-sided Wald tests. Gene names for the nearest mapped genes are italicized. Lp(a), lipoprotein a. Source data

Given the roles of HbA1c, circulating lipid levels, and SBP in our polygenic MR, we performed drug-target MR evaluating the potential of genetically proxied antidiabetics (proxying lower HbA1c levels) and blood-pressure-lowering therapies on mvAge. Results are oriented to mimic the pharmacological modulation of the drug target, antidiabetics/HbA1c lowering, PCSK9 inhibition/low-density lipoprotein cholesterol (LDL-C) lowering (per s.d., mmol l^−1^), LPL enhancement/TG lowering (per s.d., mmol l^−1^), increase in high-density lipoprotein cholesterol (HDL-C) (per s.d., mmol l^−^^1^), lowering of SBP (per s.d., mmHg) (Fig. 4 and Supplementary Tables 19 and 20). Among the antidiabetic targets, we observed beneficial relationships with mvAge for thiazolidinediones. MR estimates for sulfonylureas were also protective but less precise (*P* value < 0.05). Among LDL-C-lowering targets, PCSK9 and ABCG5/8 were each related beneficially to mvAge; HMGCR had a similar but less precise estimate. Among TG-lowering targets, ANGPTL4 inhibition and LPL enhancement were related beneficially to mvAge; as was increasing HDL-C via CETP inhibition. The protective estimates of PCSK9 inhibition, ABCG5/8 inhibition, LPL enhancement, CETP inhibition and LPA inhibition on mvAge each replicated in genetic instruments derived from independent GWAS from the Global Lipid Genetics Consortium (GLGC). (While several MR estimates showed evidence of heterogeneity, all were robust to removal of pleiotropic variants.) Similarly, we observed differences in the associations among the different classes of antihypertensive targets; for example, among genes in the renin–angiotensin–aldosterone pathway, genetically proxied angiotensinogen (AGT) inhibition was more strongly related to mvAge than genetically proxied angiotensin-converting-enzyme (ACE) inhibition.

### *Cis*-instrument MR prioritizes potential mvAge targets

**Fig. 5:**
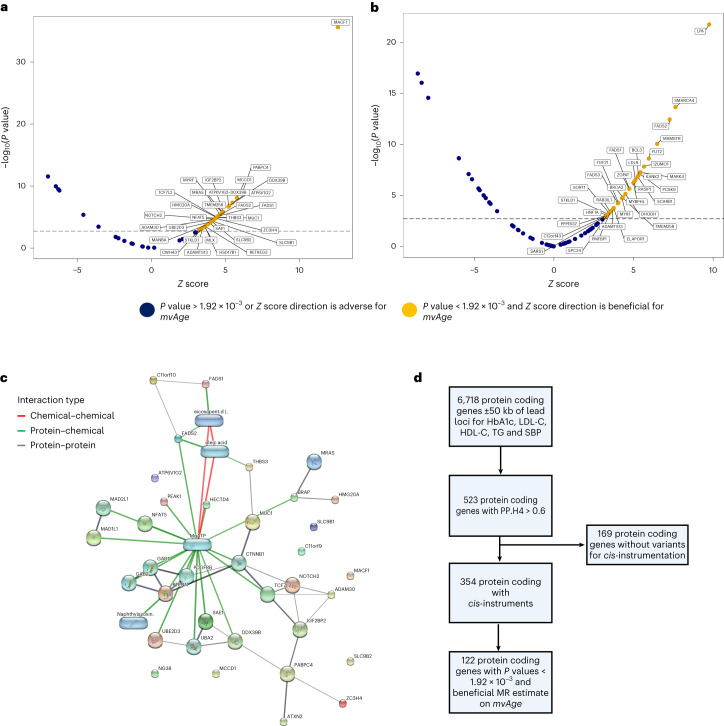
*Cis*-instrument MR results assessing the impact of protein-coding genes on mvAge through their associations with HbA1c and LDL-C. **a**, Volcano plot of the *Z* scores (versus the negative log_10_(*P* value)) of the MR estimates (beta/se) for the inverse variance weighted MR method aligned to proxy the pharmacological effect of lowered HbA1c levels. **b**, Volcano plot of the *Z* scores (versus the negative log_10_(*P* value)) of the MR estimates (beta/se) for the inverse variance weighted MR method aligned to proxy the pharmacological effect of lowered LDL-C levels. Dotted lines indicate the Bonferroni-corrected *P*-value threshold (1.92 × 10^−3^). Labeled genes are those with beneficial estimates on mvAge that surpass the Bonferroni-corrected *P-*value threshold and align with lowered HbA1c and lower LDL-C. **c**, The STITCH protein–protein and protein–chemical interactions for the 30 protein-coding genes in HbA1c. Stronger associations are annotated with thicker lines. Protein–protein interactions are represented by gray lines, protein–chemical interactions are represented by green lines, and chemical–chemical interactions are represented by red lines. **d**, Flowchart outlining the *cis*-instrument analysis pipeline (see Supplementary Methods for more details). *P* values are derived from two-sided Wald tests. Source data

**Extended Data Fig. 2:**
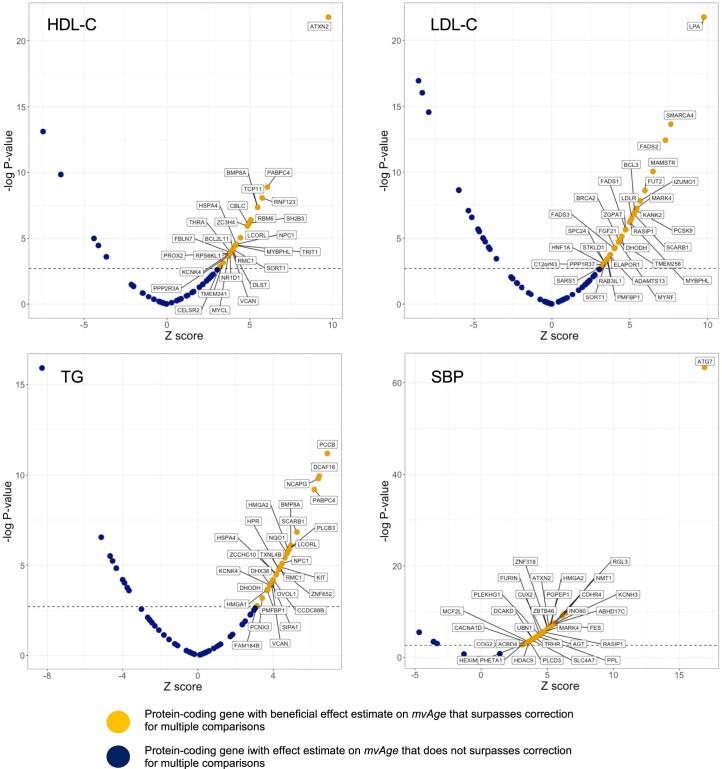
Cis-instrument Mendelian randomization assessing the impact of protein coding genes on *mvAge* through their associations with HDL-C, LDL-C, triglycerides, and SBP. Presented is a volcano plot of the Z scores (versus the negative log_10_(P-value)) of the MR estimates (beta/se) for the inverse variance weighted (IVW) MR method aligned to proxy the pharmacological effect of increased HDL-C (N = 403,943), lowered LDL-C (N = 440,546), lowered TG (N = 441,016), or lowered SBP (N = 436,419) on *mvAge* (N = 1,958,774). The dotted line indicates the Bonferroni corrected P-value threshold (1.92 × 10^−3^). Labeled genes are those with beneficial estimates on *mvAge* that surpass Bonferroni corrected P-value threshold and align with increased HDL-C, lowered LDL-C, lowered TG, or lowered SBP. HDL-C, high-density lipoprotein cholesterol; LDL-C, low-density lipoprotein cholesterol; TG, triglycerides; SBP, systolic blood pressure; MR, Mendelian randomization. Source data

**Extended Data Fig. 3:**
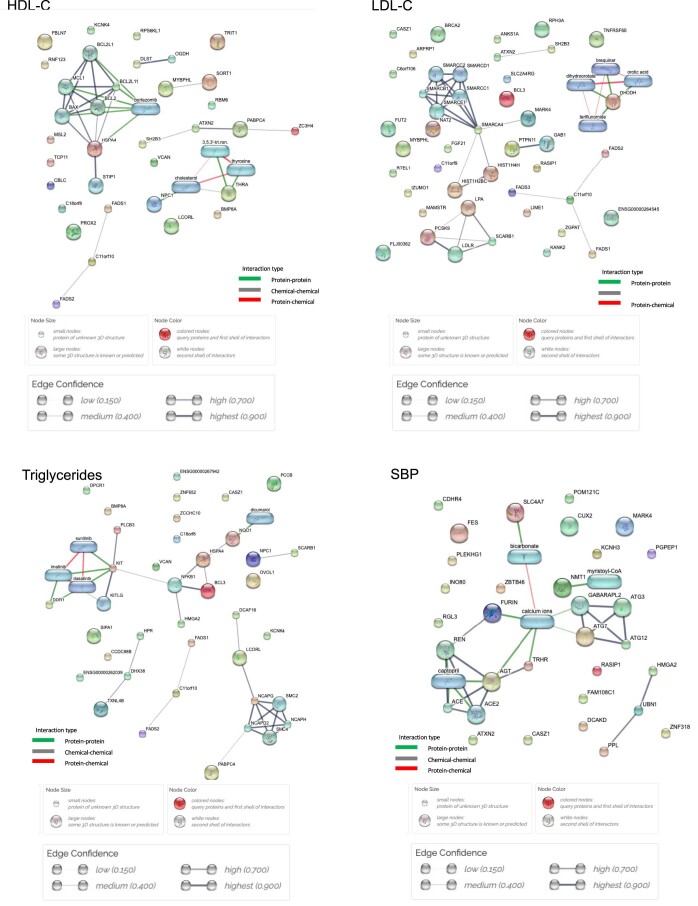
Protein-protein and protein-chemical interaction for protein coding genes located near the lead SNPs of the HDL-C, LDL-C, triglycerides, and SBP GWASs associated with *mvAge* in the cis-instrument Mendelian randomization. Analyses were performed using STITCH **(**http://stitch.embl.de/**)**. Results plotted are STITCH PPI interaction scores. The combined scores are computed by calculating the probabilities from the STITCH database sources of evidence and correcting by the probabilities of randomly observing interactions between the proteins and chemicals (see ref. ^99^ in the Reference list for additional information). Stronger associations are annotated with thicker lines. Protein-protein interactions are represented by grey lines, protein-chemical interactions are represented by green lines, and chemical-chemical interactions are represented by red lines. Per STITCH guidelines, ‘high confidence’ interactions are considered those with combined scores ≥ 0.90. HDL-C, high-density lipoprotein cholesterol; LDL-C, low-density lipoprotein cholesterol; TG, triglycerides; SBP, systolic blood pressure; MR, Mendelian randomization. Source data

**Extended Data Fig. 4:**
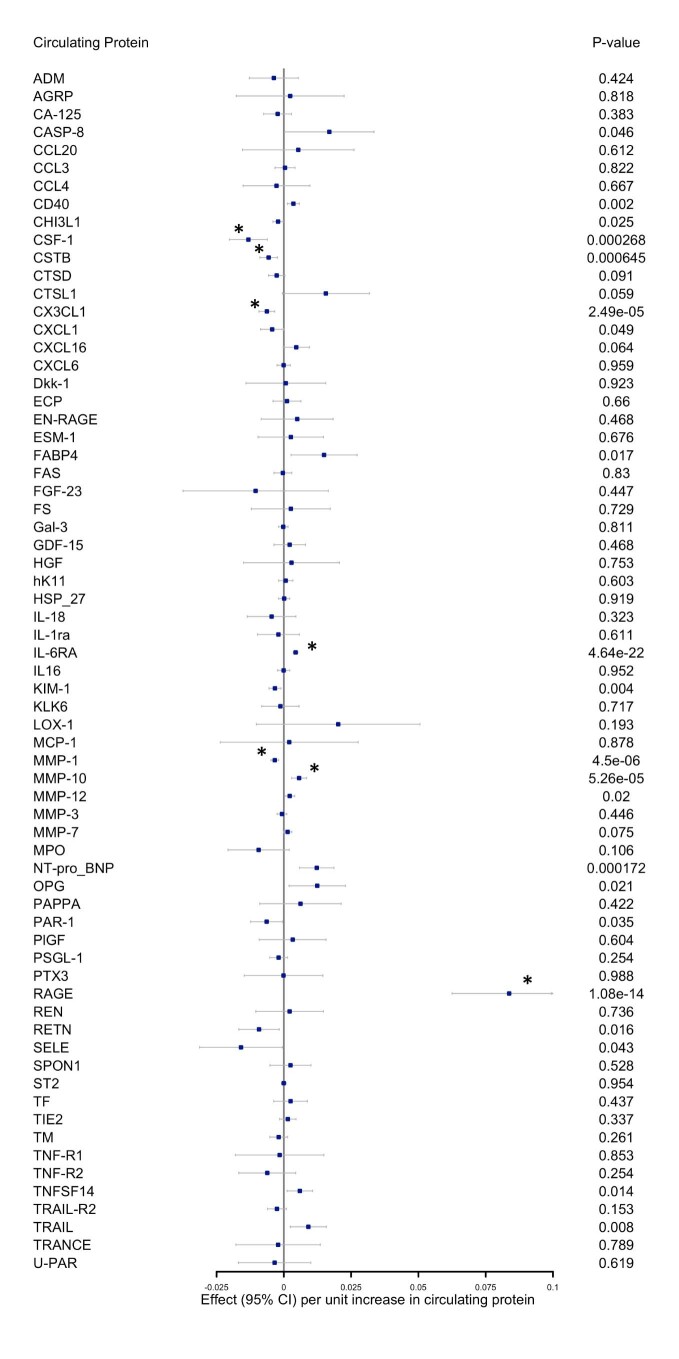
Cis-instrument Mendelian randomization analysis of 68 circulating proteins. Data presented are MR effect estimates (betas) for the inverse variance weighted (IVW) MR method and corresponding 95% confidence intervals (CIs). The impact of 68 circulating proteins on *mvAge* (N = 1,958,774) was analyzed using protein quantitative trait loci (pQTL) data derived from 30,391 participants of European ancestry in the SCALLOP Consortia dataset (http://www.olink-improve.com/). We used pQTLs associated with the respective plasma protein at P-value < 5 × 10^−8^ within or near the cis-acting locus of the target gene boundary, that is, 100 kilobases on either side of the respective gene boundary. Extracted SNPs were clumped at the LD R^2^ ≤ 0.2 threshold (250 kb) using the 1000 Genomes Phase 3 European reference population and MR IVW (random-effects analysis when there were more than three variants) and MR Egger implemented accounting for correlation between the instrument variants. MR, Mendelian randomization. Source data

Leveraging biomarker data and *cis*-instrument MR^29^, we performed a screen of several thousand protein-coding genes located near the genomic loci of the biomarkers, that is, HbA1c, HDL-C, LDL-C, TG and SBP, corresponding to the physiological responses to glucose-lowering, lipid-modulation and antihypertensive therapies. In the first stage, 523 of 6,718 genes across the 5 biomarkers demonstrated evidence of colocalization (PP.H4 > 0.6) (Supplementary Tables 21–25). We were able to *cis*-instrument and analyze 354 of the 523 genes on mvAge. Across the biomarkers, 158 genes (121 unique genes) evinced genetic relationships with mvAge, 122 with beneficial MR estimates directionally consistent with the conventional physiological response to pharmacological modulation of the biomarkers, that is, lowered HbA1c, LDL-C, triglycerides and SBP, and increased HDL-C (Supplementary Tables 26–32). Twenty-five genes located near HbA1c evinced beneficial relationships with mvAge, including *FADS1*, previously linked with glucose intolerance^30^, and also 23 near LDL-C, including replication of the *PCSK9* results, and *FGF21*, an important regulator of several metabolic pathways^31^ (Fig. 5). Twenty-three genes near HDL-C, 26 near TGs and 25 near SBP similarly showed beneficial relationships with mvAge (Extended Data Fig. 2). Several genes (for example, *ATXN2*) were related to mvAge in more than one biomarker. Thirty-two are considered ‘druggable,’^29^ and we observed drug–gene interactions, including an interaction of *FADS2* with oleic acid (Supplementary Tables 33 and 34 and Extended Data Fig. 3). Regarding replication, 36 of the 41 genes available for instrumentation in independent biomarker data replicated at *P* value < 0.05 and had directionally consistent MR estimates with the primary MR analyses (Supplementary Table 32). *Cis*-instrument/drug-target MR results of the 68 circulating proteins derived from approximately 30,000 participants in the SCALLOP OLINK data^32^ are presented in Supplementary Results, Supplementary Tables 35 and 36 and Extended Data Fig. 4.

## Discussion

Using new GWAS methods leveraging genetic correlations among correlated univariate aging-related traits, we performed a multivariate GWAS with a resultant effective sample size of 1.9 million participants and identified 20 variants not previously associated with aging, including rs268, an exonic SNP in the *LPL* locus, and rs2863761, an intronic variant not previously associated with any GWAS in the GWAS catalog. However, because no other age-related variant is in LD with rs2863761, replication will be necessary to test the robustness of this association. Next, we used fine mapping to prioritize several strongly associated variants (such as rs268), and transcriptomic imputation followed by gene-level colocalization and transcriptomic imputation-based fine mapping to identify high-confidence genes associated with mvAge, including several with evidence of involvement in aging processes (*VEGFA*^33^ and *PHB1* (ref. ^34^)).

The genetic signature of mvAge was enriched for gene sets linked with aging-related areas, including neural functioning, growth and development, and lipid metabolism. In line with the lipid-related enrichment, we used the MR framework to both identify adverse causal roles for lipid levels in aging and show that genetically modeled modulation of lipid-lowering gene targets, such as *PCSK9*, *ANGPTL4* and *LPL*, have beneficial relationships with healthy aging, suggesting potential therapeutic targets for future investigation. The association of rs268 in the *LPL* locus with mvAge, its inclusion in a 95% credible set, and the MR evidence for a role of circulating triglycerides—using both the polygenic, genome-wide instrument and instrument variants within the *LPL* locus—indicate a potential pathway through which rs268 may impact healthy aging. The results of our drug-target MR analyses proxying pharmacological lipid-lowering and antihypertensives support the hypothesis that therapeutic management of circulating lipids and blood pressure impact healthy aging in the general population not selected for cardiovascular diseases^35,36^. Overall, the gene-set enrichment and MR findings between several mvAge loci and cardiovascular health are in line with cardiovascular disease being the leading global cause of death^37^. They also likely reflect the composition cohorts underlying the input GWAS studies. For example, healthspan was defined as the incidence of the eight most common diseases in the study sample^9^, and thus is dependent on UK Biobank (UKB) selection protocols. The UKB cohort is comprised of adults between 40 and 69 years, and is enriched in cardiovascular disease and cancer, but has relatively few cases of Alzheimer’s disease^38^. This composition may explain, in part, the strong associations observed with cardiovascular health, and also suggests that other key aging signals may be missed. Therefore, future multitrait studies with different cohort composition are warranted to further our understanding of aging.

Our additional exploratory and confirmatory factor analyses aiming to further examine the relationship of the genetics of life expectancy (lifespan and longevity) and healthspan suggested that in a two-factor model, lifespan and longevity load on one factor while healthspan, frailty and EAA load on the other factor. The two factors are correlated, suggesting that there may be shared yet distinct components of life expectancy/lifespan and healthspan/frailty/epigenetic aging captured by mvAge. Because one of the major goals currently in geroscience is reducing the life expectancy–healthspan gap^39^, our findings suggest that analyzing mvAge and related shared factors in future studies, in additional populations, will improve our understanding of the genetics linking life expectancy and healthspan.

We focused downstream analyses on identifying modifiable risk factors that may facilitate public health intervention and prevention strategies, as well as extensive drug-target MR to investigate the impact of existing therapies and also identify targets for future work focused on healthy aging. There remain substantial challenges to running randomized controlled trials testing aging-related therapeutics (that is, long study duration, large sample sizes, patient selection)^40^. Especially relevant to the development of anti-aging therapeutics will be the continued investigation of the relationship between aging and age-related diseases^40^. Many diseases are due, in part, to age-related biological dysregulation^41^, and studies incorporating aspects of both aging and age-related diseases may identify drug targets and facilitate development of therapeutics that both improve healthy aging and reduce disease burden.

To the point, we found several of the novel variants not previously linked directly with aging-specific GWASs were indeed associated with important aging factors and processes, for example, rs78438918 associated with cognition, rs114298671 associated with BMI, and rs6062322 associated with blood pressure and antihypertensive medication use (Table 1). Hundreds of variants have been identified in GWASs of BMI^42^, cognition^43^, blood pressure^44^, etc., and while these are important aging factors and processes, not all of the identified variants may be directly linked with healthy aging. Because the mvAge signature represents the shared genetics of the five specific aging phenotypes, finding that a subset of variants previously implicated in aging factors and processes comprise, in part, the mvAge signature highlights potential pathways linking the aging factors and processes with healthy aging, and suggesting potentially important loci for future characterization, especially in studies linking these factors/processes with healthy aging.

Our metformin targets analysis complements previous studies showing that metformin is beneficial for healthy aging^45^. Given the early stages of the ongoing clinical studies (MILES (Metformin in Longevity Study) and TAME (Targeting Aging with Metformin)^18,46^) investigating the aging benefits of metformin, it will be several years before the studies will be concluded. Our results constitute preliminary genetic evidence and provide triangulating evidence strengthening inference for metformin’s role in aging. Preliminary analysis of the MILES data indicates that metformin induces transcriptional changes related to reduced aging^47^, and we showed that metformin had a beneficial impact on slowing epigenetic aging, together suggesting another biological mechanism that corresponds with clinical trial data from the first human study designed to reverse biological hallmarks of aging, including EAA in a population of healthy middle-aged men^48^. We found that the metformin instrument may be driven by its mitochondrial-related targets, MCI, MG3 and GDF15. Mitochondrial function is impaired in disease states and aging^49^ and it has been suggested that metformin may regulate mitochondrial functioning by mitophagy and removing damaged mitochondria^47^, which could improve aging. GDF15 has become an important target in the aging field with previous studies linking it with all-cause mortality^50^; showing that among 1,301 proteins, it was the most strongly associated with age^51^; and finding high expression among frail older individuals compared to healthy controls^52^. GDF15 is a key molecule in the human stress response^53^, and recent work found that patients with primary mitochondrial oxidative phosphorylation defects demonstrate increased resting energy expenditure, elevated stress responses (including elevated GDF15 levels) and accelerated biological aging^54^. Given the impact of glucose homeostasis on energy^55^, our results showing that HbA1c lowering via metformin’s mitochondrial targets further support evidence linking mitochondrial function and energy expenditure with accelerated aging. We note that our metformin targets were derived from data proxying their anti-hyperglycemic effects, and while it has been suggested that the main anti-aging role of metformin is mediated via its action on glucose metabolism^45^, metformin and its targets impact many pathways, including ones that have yet to be elucidated^45^. Therefore, these results should not be interpreted as complete proxies of metformin use or its mechanisms via other pathways.

The PCSK9 and other lipid-lowering findings extend work showing that life expectancy in familial hypercholesterolemic patients is shortened by 20–30 years relative to the general population^56^. While a recent meta-analysis of 38 randomized controlled trials did not find an impact of PCSK9 inhibition on all-cause mortality among study participants selected for cardiovascular diseases^57^, the studies included in the meta-analysis were potentially not long enough in duration to detect a role of PCSK9 inhibition on aging (~36.4 weeks)^57^. Ultimately, causal inference requires triangulating study designs^58^, highlighting the need for additional studies investigating the relationship of *PCSK9* expression, PCSK9 inhibition, and aging. Similarly, our blood pressure and antihypertensive target findings align with and extend previous work showing that intensive blood pressure reduction in older adults with hypertension extends life expectancy, highlighting the importance of blood pressure control to prolong patient health and well-being^59^. Our results showing that *ADRBI*—the beta-blockers target—beneficially impacts aging extend prior genetics-based analyses finding *ADRB1* beneficial in human longevity^60^.

In addition to validating important loci (for example, the *ATXN2* in mvAge findings aligns with previous genetics work implicating *ATXN2* in human longevity^6^ and showing that therapeutic modulation of *ATXN2* increases lifespan in mice^61^), our analyses extending the drug-target/*cis*-instrument MR framework using GWAS data for biomarkers to investigate possible protein-coding genes that may influence mvAge via their downstream biomarker (that is, HbA1c, circulating lipids, SBP) also identified several notable targets. For example, *FADS1* and *FADS2* are important genes in the biosynthesis of unsaturated fatty acids^62^, and protein–chemical interaction analysis showed *FADS2* has interactions with oleic acid, the main fatty acid in olive oil, a major Mediterranean diet component^63^, linked with increased lifespan and reduced age-related diseases^63^. We also found that reduced LDL-C levels by variants within the *FGF21* locus increased mvAge. *FGF21* encodes fibroblast growth factor 21, a metabolic hormone important for regulation of systems related to energy homeostasis, including lipids^31^, and increased *FGF21* expression extends lifespan in mice^64^. Interestingly, early studies of FGF21 analogs in individuals with T2D found they alleviated dyslipidemia but did not impact glycemic control^31^, and a new long-acting therapeutic compound, LLF580, was found to lower circulating LDL-C and reduce hepatic fat content in a phase 1 clinical trial of 64 obese adults (clinical trial identifier, NCT03466203) (ref. ^65^). LLF580 also showed minimal side-effects for the 12-week study with the exception of increased reporting of mild-to-moderate gastrointestinal distress^65^. Our *FGF21* results provide triangulating support for a role of *FGF21* in cardiometabolic diseases^66^ and longevity^64^, and also highlight the potential value of the recently developed extension of the drug-target MR paradigm to identifying and prioritizing novel targets for future study^67^.

The exploratory MR based on protein quantitative trait loci (pQTL) should also motivate future research into therapies to improve healthy aging because most approved pharmacotherapies target proteins^19^. We identified and replicated in independent pQTL datasets three circulating proteins, CSF-1, MMP-1 and IL-6RA. Increased circulating levels of CSF-1 and MMP-1 adversely impacted mvAge. In the Supplementary Discussion we highlight reported relationships of CSF-1, MMP-1 and IL-6RA, biomarkers and risk factors with a range of physical health diseases that further support their role in healthy aging.

The drug-target MR has limitations, including its inability to mimic certain mechanisms of action for the therapeutics. For example, the genetic estimates for PCSK9 inhibition derived from circulating LDL-C levels may not fully approximate the impact of specific drug classes, such as inclisiran, a novel small interfering RNA inhibitor of hepatic PCSK9 expression with a liver-specific mechanism of action^68^. Similarly, our antidiabetic and antihypertensive instruments may not capture pathways beyond the HbA1c and SBP biomarkers. For example, Liu et al.^69^ also showed that verapamil promoted autophagy and increased levels of calcineurin activity in *C. elegans*^69^, offering additional life-extending mechanisms through which calcium channel blockers (CCBs) may impact healthy aging; however, our SBP-derived CCB instrument precludes direct investigation of these non-blood-pressure-mediated pathways. Our formulation of the metformin targets instrument is a novel application developed in recent drug-target MR work^28^ and we underscore that the estimates reflect the metformin target-specific effects. Our metformin targets findings should be viewed in the context of triangulating study designs finding beneficial a relationship between metformin and aging. We also emphasize the results do not suggest these therapeutic targets be viewed as panaceas for improved aging and should not be considered replacements related to healthy lifestyle choices that are important for healthy aging^70^. More broadly, the MR analyses may be subject to collider bias^71^, which occurs when a third, collider variable caused by both the exposure and outcome variables in an MR distorts the true underlying association^72^. Here, potential colliders include chronic diseases or their causal risk factors that may impact analyses with outcomes related to exceptional longevity. For example, while we found that metformin target genes reduced epigenetic aging and increased exceptional longevity, it has been hypothesized that targeting frailty or therapeutics aimed at chronic diseases will not increase the limit of human lifespan^73^, suggesting that these drug target findings may be primarily driven by their impact on their indicated disease(s), which, while not directly extending the limit of human lifespan, may still have important implications for improving healthy aging, including reducing the life expectancy–healthspan gap.

We note additional study limitations. First, genomic SEM provides a composite phenotype representing the joint genetic structure of broad liability across complex traits. Therefore, resulting multivariate GWASs, including mvAge, do not have conventional units^14^. This has implications for our MR analyses; however, because MR estimates reflect lifelong estimates, clinical interpretation of numerical estimates is challenging in all MR studies^17,19^. (Further discussion regarding the contextualizing of mvAge estimates, especially as it relates to MR, with a focus on the metformin target estimates is presented in the Supplementary Discussion). Genomic SEM, like most GWAS studies, assumes an additive model for genetic variants^14^, which may not capture the impact of recessive variants that may have large adverse effects on aging^3,74^. Correlation among genes used as input for our gene-set enrichment analysis may increase the potential for type 1 errors^75^; however, at least for the lipid-related pathways, concurrent MR evidence linking both circulating lipids and lipid-lowering therapeutic gene targets strengthens the evidence for involvement of lipids in healthy aging. mvAge and downstream analyses were limited to participants of European ancestry. Therefore, we underscore the limited generalizability of study findings to populations of other ethnicities/ancestries, highlighting the need for follow-up studies validating these findings in other populations when the GWAS data becomes available. Finally, limitations may derive from the univariate GWAS data (input for the multivariate GWAS analysis); for example, the parental lifespan GWAS may reflect the common causes of death in the UK from several decades ago, which have changed over time^76^, and may not fully capture current demographic characteristics.

## Conclusions

We leveraged recently developed multivariate GWAS methods to elucidate the genetic underpinnings of the broad predisposition of healthy aging. The identified loci reflecting this underlying aging-related trait align with the current shift in geroscience toward a systems-level focus aimed at improving healthy aging and slowing aging processes^70^. Bioannotation and MR characterized putatively causal genes, cell types, biomarkers and modifiable risk factors. Drug-target MR of approved and proposed antidiabetic, lipid-modulating and antihypertensive targets highlighted important repurposing opportunities, while our extended *cis*-instrument MR screen of protein-coding genes finding more than 120 candidates will inform the prioritization of therapies for healthy aging.

## Methods

### Data sources

The univariate input GWAS data from participants of European ancestry comprising our multivariate GWAS on human aging was derived from five GWASs encompassing related aspects of human longevity, including healthspan^9^, parental lifespan^1^, exceptional longevity^8^, EAA^10^ and frailty^11^. All input GWASs have existing ethical permissions from their respective institutional review boards and include participant informed consent with rigorous quality control.

The healthspan GWAS endpoint (*n* = 300,477, 54.2% female, in the UKB) was defined as the incidence of the eight most common diseases in the study sample^9^, and the study employed Cox–Gompertz survival models with clinical events in seven disease categories (that is, cancer, myocardial infarction, chronic obstructive plumonary disease, diabetes, stroke and dementia) to determine length of healthspan. Participants having one or more of these events were considered to have completed healthspans; 84,949 participants experienced an event, completing their healthspans^9^. Our frailty data was derived from a meta-analysis of participants in the UKB (*n* = 164,610, 51.3% female, between the ages of 60 and 70) and TwinGene (*n* = 10,616, 52.5% female) cohorts^11^. The UKB frailty index was based upon an accumulation-of-deficits model^77^ using 49 self-reported UKB variables from a range of physical and mental health endpoints, symptoms, disabilities and diagnosed diseases^11^. The frailty index in the TwinGene cohort was also constructed using self-reported questionnaire data (44 deficits)^11^.

We used summary statistics from a recent parental lifespan GWAS representing 512,047 and 500,196 maternal and paternal lifespans^9^. Across cohorts, Cox survival models for mothers and fathers had been fitted and Martingale residuals of corresponding survival models were regressed against subject gene dosages to generate the GWAS. We used summary statistics from Deelen et al. assessing the genetic underpinnings of exceptional old age using 11,262 unrelated participants reaching ≥90th survival percentile and performing a GWAS comparing this extreme longevity group with 25,483 participants whose age at death was ≤60th survival percentile (*n* = 36,745, 58.0% female)^8^. Survival percentiles were based upon country-specific cohort life tables (for example, the 90th survival percentile for the United States 1920 birth cohort is 89 years of age for men and 95 years of age for women and the 60th percentile is 75 and 83, respectively)^8^.

Our EAA GWAS data came from a meta-analysis in 29 cohorts (*n* = 36,112, 58.8% female) of four separate epigenetic clocks^10^. After using cross-trait LD score regression^20^ to test the genetic correlation among each epigenetic clock and the other longevity-related univariate GWAS included in the study, we used the second-generation epigenetic clock PhenoAge, which demonstrated genetic correlations with extreme longevity, healthspan, parental lifespan and frailty, and strong genomic characterization (Supplementary Table 1). We reversed the frailty and PhenoAge coding to generate positive correlations with the other aging-related traits.

### Sample overlap

At least 571,260 unique genomes are represented in our analysis (Supplementary Table 1), accounting for maximum potential overlap between the study cohorts contributing to the five aging GWAS study cohorts included in the multivariate GWAS analysis, as well as the potential overlap of UKB and other non-UKB UK cohorts, and the overlap of genomes underlying the parental lifespans cohorts.

### Genomic SEM

We used genomic SEM implemented in the GenomicSEM R package v.0.0.5 to perform the multivariate GWAS analysis of healthspan, exceptional longevity, parental lifespan, frailty and PhenoAge, investigating a broad genetic liability underlying these aging-related traits. Genomic SEM is a recently developed multivariate method enabling investigation of multiple potential multivariate models of the underlying architecture of the traits of interest^14^. (Full technical details of genomic SEM methods are described in Supplementary Methods.) Genomic SEM is not biased by sample overlap, that is, UKB participants in multiple input GWASs, or imbalanced sample size^14^. Genomic SEM also facilitates identification of variants only influencing some but not all of the complex traits, and which therefore do not represent a broad cross-trait liability^14^.

Genomic SEM proceeds in two stages. Stage 1 estimates the empirical genetic covariance matrix and corresponding sampling covariance matrix. We prepared the aging-related GWAS summary statistics for stage 1 and used the multivariate extension of cross-trait LD score regression^14,20^ to generate the empirical genetic covariance matrix between the five traits as input for the SEM common factor model^14^ (Supplementary Table 3). Stage 2 specifies an SEM that minimizes the hypothesized covariance matrix and the empirical covariance matrix calculated in stage 1 (ref. ^14^). Here, our primary study aim was to identify a genetic signature underlying the five aging-related traits; we therefore tested a one-factor model. Model fit was assessed using the SRMR, model *χ*^2^, the Akaike information criterion and the CFI (Supplementary Table 4)^78^.

Preparing the multivariate summary statistics for multivariate GWAS, we used the recommended default parameters implementing LD score regression, including removing SNPs with MAF >0.01 (linkage disequilibrium score regression inflates standard errors of estimates with low MAF) and information scores <0.9, and filtering SNPs to HapMap3, using the 1000 Genomes Phase 3 EUR panel (Supplementary Table 5). The summary statistics are restricted to HapMap3 SNPs only for estimating genetic covariance and sampling covariance matrix in LD score regression. We use all autosomal SNPs from the five input aging-related GWASs passing recommended default quality control filters for the multivariate GWAS analysis, filtering to the 1000 Genomes Phase 3 EUR panel, removing SNPs with MAF <0.01 (prone to error due to fewer samples within the genotype cluster and LD score regression standard errors for these SNPs tend to be high), SNPs with effect values estimated to be exactly equal zero (so as to avoid compromising matrix inversion necessary for genomic SEM), SNPs not matched with the reference panel, and SNPs with mismatched alleles. After quality control, 6,793,898 SNPs common to all input GWASs remain in the multivariate summary statistics taken forward to run the multivariate GWAS. Applying the appropriate common factor SEM specification, the individual autosomal SNP associations are incorporated into the genetic and associated sample covariance matrices to generate the multivariate genome-wide analysis (mvAge) of the shared covariance across the five input aging-related GWASs^14^.

### *Q*_SNP_ heterogeneity

To evaluate whether the mvAge SNP associations are appropriately modeled within a multivariate SEM framework, *Q*_SNP_ heterogeneity statistics are calculated^14^. The null hypothesis of the *Q*_SNP_ test is that the SNP associations on the single-phenotype GWASs are statistically mediated by mvAge^14^. Therefore, significant *Q*_SNP_ tests in mvAge would suggest that the SNP impacts the single-phenotype GWASs by pathways other than the shared genetics of aging modeled by mvAge^14^. We used a Bonferroni-corrected *P*-value threshold of 9.62 × 10^−4^, correcting for 52 lead SNPs, to evaluate *Q*_SNP_ heterogeneity.

### Exploratory two-factor model analysis

Increases in life expectancy have outpaced improvements in healthspan resulting in an approximately nine-year life expectancy–healthspan gap^79^. This relationship between life expectancy and healthspan has important implications for public health intervention/prevention strategies and drug discovery and repurposing endeavors^39^. Given that our input aging-related GWAS data encompass aspects of life expectancy (parental lifespan and extreme longevity), biological aging (EAA) and healthy aging (healthspan and frailty), as sensitivity analyses, we constructed a two-factor genomic SEM model assessing the relationship between healthspan and longevity, and investigated whether our shared aging factor—mvAge—encompasses aspects of life expectancy and healthy aging.

### Defining genomic loci and determining novel variants

We used ‘functional mapping and annotation of genetic associations’ implemented in functional mapping and annotation of genetic associations (FUMA)^80^ v.1.3.5e to identify genomic loci, and lead/sentinel SNPs in LD (*R*^2^ < 0.1) associated with mvAge at genome-wide significance (*P* value < 5 × 10^−8^). A locus was defined by lead SNPs within a 250 kb range and all SNPs in high LD (*R*^2^ > 0.6) with at least one independent SNP. First, we extracted the summary statistics for these lead mvAge SNPs from the input univariate GWASs to assess the strength of their associations. We also compared lead SNPs and loci with the original univariate GWAS and defined loci to be novel if they were >1 Mb from loci identified in the univariate GWAS data. To determine whether any of the 52 lead SNPs in mvAge showed evidence of pleiotropic associations, we also performed a look-up of published GWAS-significant associations (*P* value < 5 × 10^−8^) in the GWAS Catalog^81^.

### Fine mapping and transcriptomic imputation

We used SuSIE^82,83^ and FINEMAP^84^ implemented in the R package, echolocatoR^85^ v.2.0.3 to identify the most plausible causal variants associated with mvAge. We used a 250 kb window around the lead SNP in the 38 genomic loci and a probability threshold of 0.95 to define credible sets of potentially causal variants. echolocatoR defines a ‘consensus SNP’ as a variant included in both SuSIE and FINEMAP^85^, calculates an average posterior probability and determines an average credibility set (set to 1 when the mean SNP-wise posterior probability across SuSIE and FINEMAP exceed 0.95 and 0 otherwise^85^). Next, we performed a TWAS to prioritize genes associated with mvAge. We used the TWAS FUSION method^86^ and used TWAS weights for 37,920 precomputed expression quantitative trait loci features (that is, gene/tissue pairs) from GTEx v.8 (ref. ^86^). Our mvAge had sufficient variants to analyze 36,149 of the 37,920 features. We took forward TWAS genes associated with mvAge surpassing Bonferroni correction for multiple comparisons (*P* value < 1.38 × 10^−6^) for additional analysis, including colocalization^24^ and fine mapping using the FOCUS method designed for TWAS studies^25^. We prioritized ‘high-confidence’ mvAge genes identified by FUSION based upon additional evidence for colocalization and fine mapping. Following previous work^87^, we considered TWAS-significant genes associated with mvAge that also colocalized (PP.H4 >0.6) and are likely to be causal (FOCUS posterior inclusion probability >0.5). See Supplementary Methods for additional information about fine mapping and transcriptomic imputation.

### Gene-set and disease ontology enrichment

We used MAGMA^26^ with data from GTEx (v.8) to perform gene-based and gene-set analyses and investigated the potential relationships of mvAge with Mendelian disease genes and associated pathways with MendelVar^88^. See Supplementary Methods for further details.

### Cell-type enrichment

To identify etiological cell types associated with mvAge, we integrated single-cell RNA sequencing (scRNA-seq) data using cell-type expression-specific integration for complex traits (CELLECT)^89^. We used scRNA-seq data from Tabula Muris^90^, a database containing transcriptomic data from 100,000 cells and 20 organs and tissues of *Mus musculus*. We prepared the Tabula Muris scRNA-seq data using CELLEX^89^, calculating expression specificity likelihood scores for each gene following normalization and preprocessing^89^. Using CELLECT’s default settings, we performed the cell-type enrichment with MAGMA. In CELLECT, MAGMA measures the extent to which genetic associations with a phenotype increase as a function of gene expression specificity for a given cell type^89^. We categorized our cell types following the nomenclature used in the original Tabula Muris study^90^ and used a false discovery rate (FDR) threshold of 0.05.

### Mendelian randomization

All MR analyses have been reported in accordance with the STROBE-MR guidelines^91^ (Supplementary Checklist). We implemented MR using MendelianRandomization R package v.0.7.0 and TwoSampleMR package v.0.5.6.

### Polygenic MR

**Extended Data Fig. 5:**
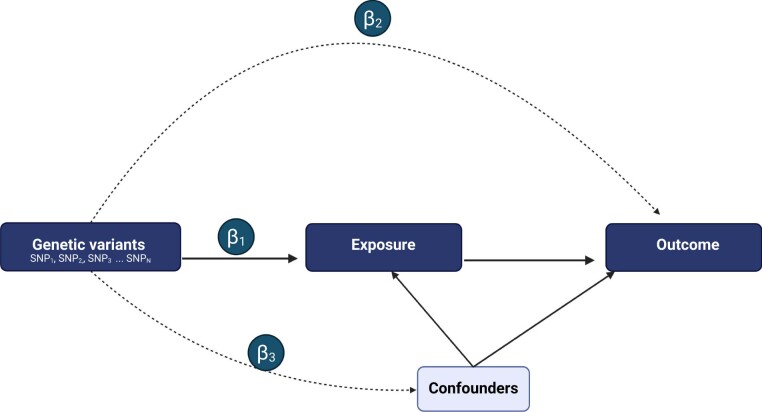
Mendelian randomization model overview (directed acyclic graph). B_2_ is the genetic association of interest, estimated by B_2_ = B_1_/ B_3._ B_1_ and B_3_ represent the estimated MR association of the genetic variants on the exposure and the outcomes. MR assumes that the genetic variants comprising the instrument for the exposure only impact the outcome of interest via the exposure and not directly, or via confounders (dotted lines). MR, Mendelian randomization.

Extended Data Fig. 5 presents a graphical overview of the analyses. To investigate whether mvAge was causally influenced by lifestyle factors and circulating biomarkers, we performed MR with 73 risk factors and biomarkers derived from GWAS with participants of European ancestry (Supplementary Table 37). Because the focus of our downstream analyses is the identification of targets for intervention, prevention and therapeutic strategies for improved aging, we selected modifiable risk factors that would inform public health initiatives^92^. Reliable causal biomarkers to identify the consequences of aging also are needed^93^. Therefore, we curated a range of biomarkers (for example, lipids, blood pressure, markers of inflammation). Given mvAge and many of the exposures included in the MR analyses are derived from the UKB, sample overlap may introduce bias^94^. We thus applied the MR Lap method, which accounts for sample overlap (even when the exact overlap percentage is unknown) and also assesses weak instrument bias and winner’s curse^27^, as an additional sensitivity test for these MR analyses. We used a Bonferroni-corrected threshold *P* value = 6.85 × 10^−4^ adjusting for 73 comparisons. See Supplementary Methods for more detail regarding motivation, instrumentation and MR assumptions.

### Drug-target MR of metformin gene targets

Given the polygenic MR results showing an adverse relationship of HbA1c and mvAge, we investigated whether HbA1c lowering via metformin target genes may improve mvAge. In line with recent work by Zheng et al.^28^ proxying the impact of metformin target genes on Alzheimer’s disease risk, we first identified five primary metformin targets (AMPK, MCI, MG3, GSD15 and GLP1) from the literature^95–97^. We used the ChEMBL database^98^ to identify genes related to the mechanism of action for the five metformin target genes (Supplementary Table 38). We extracted variants within 100 kb of the gene boundaries (*cis*-instrumentation) from the GWAS of circulating HbA1c levels used for the polygenic MR (UKB participants of European ancestry, *n* = 361,194) (ref. ^99^). We clumped extracted SNPs at the LD *R*^2^ < 0.2 threshold (250 kb) using the 1000 Genomes Phase 3 EUR reference population^100^, and calculated *F* statistics to evaluate instrument strength (Supplementary Table 39). We then performed MR IVW (random-effects analysis performed when there were more than three variants) and MR Egger analyses accounting for the correlation between our instrument variants (the requisite correlation matrices for the analyses were generated using the 1000 Genomes Project EUR population as reference^100^) to increase statistical precision by including additional, partially independent variants in the drug-target instruments^19,67^. To facilitate interpretation of HbA1c-lowering mechanisms of metformin, we scaled MR estimates to correspond to a lowering effect in the HbA1c GWAS data. We performed additional sensitivity analyses. First, we tested each metformin target separately. Second, we removed variants associated with T2D at nominal statistical significance (*P* value < 0.05), evaluating whether T2D variants are driving the observed metformin–mvAge relationship. Third, we performed an additional MR, clumping metformin targets at LD *R*^2^ of 0.001. Fourth, we used a second metformin instrument using variants comprising the Zheng et al. variants. Finally, given the reported impact of metformin on slowing epigenetic aging^48^, we analyzed the PhenoAge GWAS and other univariate GWAS data to determine whether there was evidence of a univariate GWAS signal.

### Drug-target MR of cardiometabolic drug classes

**Extended Data Fig. 6:**
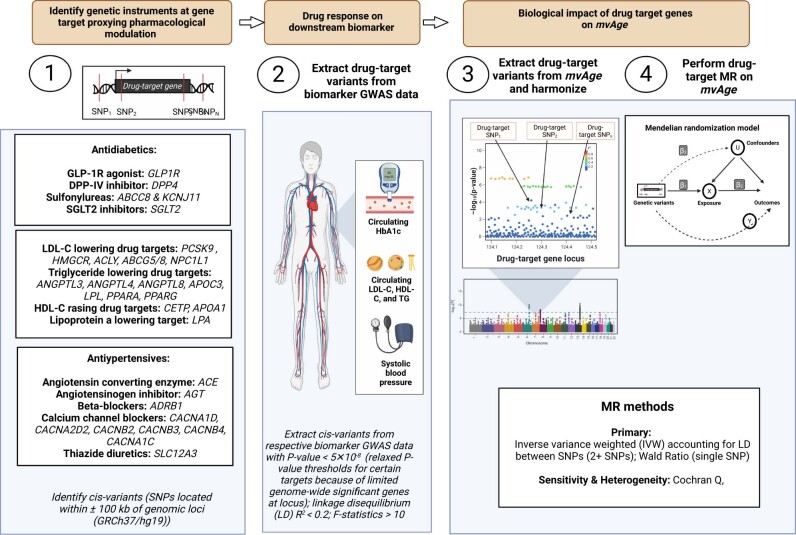
Drug-target Mendelian randomization analysis overview of anti-diabetics, lipid-modulating targets, and antihypertensives. Diagram depicts flow diagram and details of the drug-target MR analyses of the cardiometabolic targets on *mvAge* (N = 1,958,774). Prior to step 1, colocalization analysis was employed to prioritize protein-coding targets for the screen. See **Methods** and Supplementary Methods for details on selection and identification of the individual targets in the three broad drug classes (anti-diabetics, lipid-modulating targets, and antihypertensive). In step 2, cis-instrumentation was performed using genome-wide association study (GWAS) of biomarkers that are the primary indications of pharmacological modulation of these targets. For antidiabetics, GWAS data of HbA1c (N = 344,182) was used; for lipid-modulating targets, several lipid subfractions including LDL-C (N = 440,546), triglycerides (N = 441,016), and HDL-C (N = 403,943) were used; and for antihypertensives, GWAS data of SBP (N = 436,419). Independent variants (LD *R*^*2*^ < 0.2) at P-values < 5 × 10^−8^) were extracted, and cis-instruments constructed for each target, which exposure variants were then extracted from the *mvAge* GWAS (outcome), harmonized, and then analyzed using multiple MR methods (steps 3 and 4). See **Methods** and Supplementary Methods for further details. MR, Mendelian randomization; LD, linkage disequilibrium. LDL-C, low-density lipoprotein cholesterol; HDL-C, high-density lipoprotein cholesterol; TG, triglycerides; SBP, systolic blood pressure.

Extended Data Fig. 6 presents a graphical overview of the analyses. Given the MR findings showing that elevated HbA1c, lipid levels and blood pressure demonstrate adverse relationships with mvAge, we performed drug-target MR using downstream biomarkers (that is, HbA1c, circulating lipids and SBP) proxying pharmacological modulation via therapeutics lowering HbA1c, lipids and blood pressure. Briefly, we proxied pharmacological modulation of these drug targets by extracting SNPs *cis*-acting loci (±100 kb of gene boundaries) associated with their respective biomarker, that is, the primary physiological response to pharmacological modulation of that target: antidiabetics targets were extracted from HbA1c data; *PCSK9, HMGCR*, *ACLY*, *ABCG5/8* and *NPC1LC* SNP effect estimates were extracted from LDL-C; *ANGPTL3*, *ANGPTL4*, *ANGPTL8*, *APOC3*, *PPARA* and *LPL* SNP effect estimates were extracted from TG; *APOA1* and *CETP* SNPs were extracted from HDL-C; *LPA* variants were extracted from Lp(a) GWAS; and variants in the antihypertensive targets were extracted from the SBP (Supplementary Tables 40–42 contain instrument information). Supplementary Methods provides more detail about selection criteria for drug-target genes.

### Drug-target MR methods

We clumped drug targets as described for the metformin analyses (LD *R*^2^ < 0.2 threshold (250 kb) using the 1000 Genomes Phase 3 EUR reference), and performed correlated MR as detailed above to increase statistical precision by including additional, partially independent variants in the drug-target instruments^19,67^. To facilitate interpretation of these HbA1c-lowering, lipid-modulating and SBP-lowering therapeutic targets, we scaled MR estimates to correspond to the physiological impact in the respective downstream biomarkers. We used a Bonferroni-corrected threshold *P* value = 0.002, adjusting for 25 total drug targets tests. See Supplementary Methods for more details regarding drug-target MR.

### *Cis*-instrument MR screen of cardiometabolic genes

**Extended Data Fig. 7:**
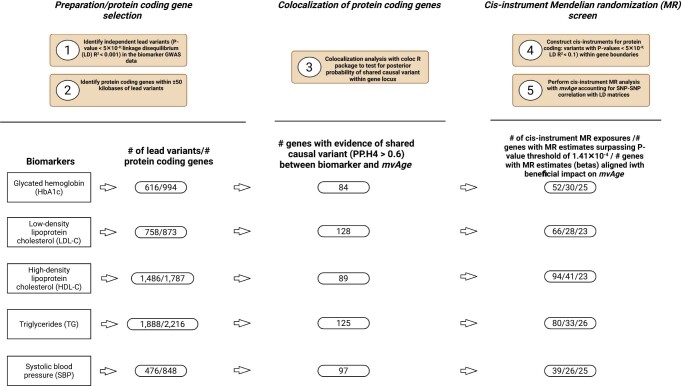
Analysis overview of cis-instrument Mendelian randomization screen of protein coding genes associated with either HbA1c, LDL-C, HDLC, triglycerides, or SBP. Results show number of protein-coding genes including in the stages of the colocalization and cis-instrument MR screens. Exposure genome-wide association study data for each of the biomarkers came from HbA1c (N = 344,182); several lipid subfractions including LDL-C (N = 440,546), triglycerides (N = 441,016), and HDL-C (N = 403,943); and SBP (N = 436,419). Outcome data was *mvAge* (N = 1,958,774). HbA1c, glycated hemoglobin; HDL-C, high-density lipoprotein cholesterol; LDL-C, low-density lipoprotein cholesterol; TG, triglycerides; SBP, systolic blood pressure; MR, Mendelian randomization.

Extended Data Fig. 7 presents a graphical overview. Given the strong MR evidence linking mvAge with both the polygenic measures of circulating lipids, HbA1c and SBP, corresponding drug-target MRs with the gene targets for metformin, and approved lipid-lowering and blood-pressure-lowering therapeutics, we performed *cis*-instrument/drug-target MR screens evaluating the causal impact of the lipid subfractions, HbA1c and SBP via the action of protein-coding genes located near the genomic loci of these biomarkers. Results of these analyses are targets that may impact mvAge through glycated hemoglobin, lipids or blood pressure. This approach has recently been developed, identifying and validating 30 gene targets exhibiting beneficial associations with lipids that may reduce coronary artery disease risk^67^. The aim of these analyses is to provide genetic evidence supporting targets that may be important to inform future mechanistic investigation for therapeutics that may improve healthy aging.

We used the same lipids, HbA1c and SBP GWAS data as used for the drug-target MR analyses described above. First, we extracted lead variants with *P* values < 5 × 10^−8^ (LD *R*^2^ < 0.1) associated with their respective GWAS biomarker (the same HbA1c, lipids and SBP GWAS data used for the drug-target MR analyses outlined above) and identified protein-coding genes located within 50 kb of the lead variants (Supplementary Tables 43–47). We then performed colocalization analyses to assess the posterior probability of a shared genetic signal between mvAge and biomarkers at the protein-coding gene locus^24^. We considered genes with posterior probabilities (PP.H4 >0.6) to have evidence of shared causal variants between mvAge and the respective biomarker at the gene. We took these protein-coding genes (523 across 5 biomarkers) forward to *cis*-instrumentation. We constructed *cis*-instruments for the protein coding using genetic variants (LD *R*^2^ < 0.2) associated with their respective biomarkers at conventional genome-wide statistical significance (*P* value < 5 × 10^−8^) within gene boundaries and SNP *F* statistics >10 (indicating strong instruments and evidence that MR estimates are unlikely to be subject to weak instrument bias); 354 of 523 protein-coding genes identified by colocalization had variants meeting these criteria. We performed MR accounting for correlation between instruments to increase estimate precision^19,67^, and used a Bonferroni-corrected threshold *P* value = 1.41 × 10^−4^, adjusting for the 354 genes tested. We were able to perform replication analyses using independent HbA1c (ref. ^101^) and lipids GWAS summary data^102^ for 41 of the 132 protein-coding genes identified by *cis*-instrument MR (an independent GWAS was not available for SBP, and thus we were not able to replicate these genes). Next, we assessed the potential therapeutic actionability of these protein-coding genes in several ways, including assessing druggability (Supplementary Table 48)^29^, protein–chemical interactions with the STITCH interaction database^103^ and drug–gene interactions with DGIdb^104^. See Supplementary Methods for more detail.

### Proxying circulating proteins

To complement the drug-target MR analyses described above leveraging biomarker data, we next explored the causal role of circulating proteins measured in 30,391 participants of European ancestry from the first results provided by the SCALLOP consortium^105^, which used the Olink platform to perform a pQTL mapping of plasma proteins to investigate additional possible anti-aging therapeutic opportunities that may be useful for future investigation. Clumping, harmonization and MR analysis proceeded as above, including several proteins instrumented by a single variant. We used the Wald Ratio method^106^ to obtain MR estimates (Supplementary Tables 49 and 50). See Supplementary Methods for more detail.

### Statistics and reproducibility

Methods describes the statistical methods used in this study to analyze the data. The statistical tests used in this study, except gene-set and cell-type enrichment, were two-sided. R v.4.0.2 was used for data processing and analysis unless otherwise specified. Predetermination of sample size, experiment randomization and blinding of investigators to experiments were not applicable for this type of study.

### Reporting summary

Further information on research design is available in the Nature Portfolio Reporting Summary linked to this article.

## Supplementary information

Supplementary Information Table of Contents, Supplementary Results, Discussion, Methods, STROBE Checklist, References, Figs. 1–33 and table captions.

Reporting Summary

Supplementary Table Supplementary Tables 1-50

## Data Availability

All analyses were based upon publicly available data. Summary-level statistics for the mvAge GWAS generated in this study are available at https://zenodo.org/record/7926323. Summary-level statistics for longevity are available at https://www.longevitygenomics.org/downloads; parental lifespan, https://datashare.ed.ac.uk/handle/10283/3209; healthspan, https://www.gwasarchive.org/; the frailty index, https://figshare.com/articles/dataset/Genome-Wide_Association_Study_of_the_Frailty_Index_-_Atkins_et_al_2019/9204998; and the epigenetic clocks, https://datashare.ed.ac.uk/handle/10283/3645. GTEx weights for FUSION analyses are available at https://gusevlab.org/projects/fusion/. Single-cell gene expression data from the Tabula Muris study are available at https://tabula-muris.ds.czbiohub.org/. Summary-level statistics used for Mendelian randomization analyses are accessible in the IEU Open GWAS Project at https://gwas.mrcieu.ac.uk/ using the IEU Open GWAS Project IDs (provided in Supplementary Table 37 accompanying the manuscript). Circulating protein levels from the SCALLOP Consortium are available at https://zenodo.org/record/2615265#.ZGEzyezMLN0. All other data supporting the findings of this study are available from the corresponding author upon reasonable request.

## Source data

Source Data Fig. 2 Excel file containing data underlying Fig. 2.
Source Data Fig. 3 Excel file containing data underlying Fig. 3.
Source Data Fig. 4 Excel file containing data underlying Fig. 4.
Source Data Fig. 5 Excel file containing data underlying Fig. 5.
Source Data Extended Data Fig. 1 Excel file containing data underlying Extended Data Fig. 1.
Source Data Extended Data Fig.2 Excel file containing data underlying Extended Data Fig. 2.
Source Data Extended Data Fig.3 Excel file containing data underlying Extended Data Fig. 3.
Source Data Extended Data Fig.4 Excel file containing data underlying Extended Data Fig. 4.

## Code availability

The software used in this study is publicly available and accessed without restriction. R package GenomicSEM v.0.0.5 is available at https://github.com/GenomicSEM/GenomicSEM. For implementing Mendelian randomization analyses, R package TwoSampleMR v.0.5.6 is available at https://mrcieu.github.io/TwoSampleMR/; R package MendelianRandomization v.0.7.0: https://cran.r-project.org/web/packages/MendelianRandomization/index.html; and R package MRlap v.0.0.3.0: https://github.com/n-mounier/MRlap. STITCH is available at http://stitch.embl.de/. R FUSION Pipeline v.1.4.2 for TWAS analysis is available at http://gusevlab.org/projects/fusion/. Python package FOCUS v.0.6.10 for FOCUS fine mapping for FUSION is available at http://github.com/bogdanlab/focus/. R package coloc v.5.1.0.1 for colocalization is available at https://cran.r-project.org/web/packages/coloc/index.html. Python package CELLECT v.1.3.0 for single-cell enrichment analyses is available at https://github.com/perslab/CELLECT. Python package CELLEX v.1.2.1 for single-cell processing is available at https://github.com/perslab/CELLEX. R package echolocatoR v.2.0.3 for fine mapping using SuSIE and FINEMAP is available at https://github.com/RajLabMSSM/echolocatoR. MendelVar is available at https://mendelvar.mrcieu.ac.uk/. FUMA and MAGMA v.1.4.0 are available at https://fuma.ctglab.nl/. R v.4.2.1 and Python v.3.8 were used to format data for analyses. Supplementary Figs. 1–21 were generated using FUMA v.1.4.0. Supplementary Figs. 22–33 were generated using R package echolocatoR v.2.0.3.
