# Supplementary material for: Multivariate genome-wide analysis of aging-related traits identifies novel loci and new drug targets for healthy aging
Source: Nat Aging. 2023 Aug 7;3(8):1020–35. doi: 10.1038/s43587-023-00455-5 (PMC10432278; doi:10.1038/s43587-023-00455-5)
Supplement: Supplementary file 1 — Table of Contents, Supplementary Results, Discussion, Methods, STROBE Checklist, References, Figs. 1–33 and table captions. [file 43587_2023_455_MOESM1_ESM.pdf]

nature aging

Article

https://doi.org/10.1038/s43587-023-00455-5

Multivariate genome-wide analysis of aging-related traits identifies novel loci and new drug targets for healthy aging

In the format provided by the authors and unedited

SUPPLEMENTARY INFORMATION

Table of Contents

DESCIPTION OF SUPPLEMENTARY TABLES ***3***

SUPPLEMENTARY RESULTS ***8***

Mendelian randomization of modifiable risk factors. **8**

Drug-target MR using pQTLs identifies CSF-1, MMP-1, and IL-6RA **8**

SUPPLEMENTARY DISCUSSION ***8***

mvAge **units in relation to metformin targets.** **8**

MR of lifestyle risk factors and biomarkers. **9**

pQTL-based cis-instrument MR highlights potential circulating proteins as mediators of aging. **10**

SUPPLEMENTARY METHODS ***10***

Genomic structural equation modeling (Genomic-SEM) background and overview. **10**

Structured covariance models and factor analysis. **10**

Assessing model fit. **13**

Confirmatory factor analysis. **13**

Effective sample size calculation. **13**

Heterogeneity testing (QSNP). **13**

Exploratory two-factor model analysis with Genomic-SEM to assess the genetic relationship between healthy aging and longevity. **14**

Fine-mapping. **14**

Gene prioritization using transcriptomic imputation. **15**

Gene set enrichment. **16**

Disease and Phenotype Ontology Enrichment. **16**

Mendelian randomization (MR) ***16***

Mendelian randomization assumptions. **16**

Polygenic Mendelian randomization inclusion rationale. **16**

Lifestyle risk factors. 17

Biomarkers: circulating lipids, glycemic markers, and blood pressure. 17

Biomarkers: immune cells and markers of inflammation status. 18

Biomarkers: markers of liver function. 18

Biomarkers: organ structure and function. 18

Biomarkers: markers of iron status. 18

Biomarkers: miscellaneous. 18

Polygenic Mendelian randomization methods. **18**

Sample independence.. 19
Drug-target MR of antidiabetics, lipid-modulating, and blood pressure-lowering gene targets 19
Antidiabetics gene instrument selection. 19
Lipid-modulating gene targets. 20
Antihypertensive drug targets... 20
Drug-target MR statistical methods. 20
Sample overlap. 21
Cis-instrument Mendelian randomization of protein-coding genes in biomarker data background 21
Selection of drug target genes and statistical analysis. 21
Intersection of genes with drug targets. 22
Cis-instrument Mendelian randomization proxying circulating proteins............................... 22
SUPPLEMENTARY CHECKLIST: STROBE-MR Reporting Guidelines. 23
1. TITLE and ABSTRACT 23
INTRODUCTION 23
2. Background 23
3. Objectives 23
METHODS.. 23
4. Study design and data sources 23
5. Assumptions 24
6. Statistical methods: main analysis 24
7. Assessment of assumptions 24
8. Sensitivity analyses 24
9. Software and pre-registration 24
RESULTS 24
10. Descriptive data 24
11. Main results 25
12. Assessment of assumptions 25
13. Sensitivity and additional analyses 25
DISCUSSION.. 25
14. Key results 25

15. Limitations 25
16. Interpretation 25
17. Generalizability 26
OTHER INFORMATION 26
18. Funding 26
19. Data and data sharing 26
20. Conflicts of Interest 26
SUPPLEMENTARY FIGURES 27
REFERENCES 60

DESCIPTION OF SUPPLEMENTARY TABLES

Supplementary Table 1
Aging-related phenotypes included in exploratory and final specifications of Genomic structural equation model (GSEM). Phenotypes explored, i.e., Frailty Index, Healthspan, Parental Lifespan, Extreme Longevity, Phenotypic Age Acceleration, Hannum Age, GrimAge, and Intrinsic Epigenetic Age Acceleration (IEAA). Linkage disequilibrium score regression (LDSC or LDSR) statistics presented. Cohort demographics described.

Supplementary Table 2
Linkage disequilibrium score regression (LDSC) estimates of heritability and genetic correlations of aging-related phenotypes. Estimated using LDSC function and LD Scores calculated by Bulik-Sullivan et al. including only HapMap3 SNPs with minor allele frequency (MAF) > 0.01; and standard errors estimated using a block jackknife over SNPs.

Supplementary Table 3
Exploratory factor analysis (EFA). Implementing multivariable LDSC, then exploratory factor analysis (EFA) informing subsequent confirmatory factor analysis.

Supplementary Table 4
Confirmatory factor analysis (CFA) for common factor and two factor models. Fitting both common factor and two factor models indicated by EFA, implemented in Genomic SEM.

Supplementary Table 5
Summary of quality control (QC) filtering applied to GWAS summary statistics prior to multivariate GSEM analysis. Removing inadmissible alleles; variants with effect values exactly 0 compromising matrix inversion necessary for Genomic SEM; variants with MAF < 0.01 prone to error as fewer samples within genotype cluster, with LD score standard errors that tend to high; multiallelics or duplicates; variants not matched to 1000 Genomes Phase 3 European reference panel.

Supplementary Table 6
Characterization and annotation of significant hits in multivariate aging GWAS (*mvAge*). Independent significant SNPs identified. Lead SNPs and genomic risk loci defined. Functional consequences on genes (ANNOVAR), CADD score, RegulomeDBscore, 15 chromatin state (127 tissue/cell types) annotated.

Supplementary Table 7
Lead multivariate aging GWAS (*mvAge*) SNPs annotated in the five input aging-related GWASs.

Supplementary Table 8
GWAS Catalog look-up of the multivariate aging GWAS lead SNPs and independent significant SNPs (performed November 22, 2022). Implemented using FUMA GWAS SNP2GENE.

Supplementary Table 9
GWAS Catalog look-up of the multivariate aging GWAS 20 novel lead SNPs and independent significant SNPs (performed November 22, 2022). Implemented using FUMA GWAS SNP2GENE.

Supplementary Table 10
SuSiE and FINEMAP fine-mapping of multivariate aging (*mvAge*) GWAS lead SNPs. Fine-mapping implemented to identify most plausible causal variants. SNPs contained within each 95% credible set for *mvAge* loci are listed, along with their inclusion probability and functional annotation.

Supplementary Table 11
Transcriptome-wide association study (TWAS). TWAS implemented to integrate GWAS and gene expression datasets to identify gene-trait associations and prioritize causal genes at GWAS loci.

Supplementary Table 12
Multi-marker analysis of genomic annotation (MAGMA) gene-based results. MAGMA implemented with data from GTEx (version 8) to perform gene-based and gene-set analyses. Mapping SNPs to 18,649 protein coding genes within 10 kb of lead SNPs accounting for LD between SNPs (using the1000G reference panel).

Supplementary Table 13
Gene-set enrichment analysis (GSEA) using MAGMA-derived genes. Gene-to-function (G2F) gene-set analyses implemented using genes identified with MAGMA to evaluate potential relationships between *mvAge* and mapped genes from MSigDB gene sets, including Reactome, and Gene Ontology (GO).

Supplementary Table 14
Cell-type enrichment analysis. To identify etiological cell types associated with *mvAge*, single-cell RNA-sequencing (scRNA-seq) from Tabula Muris was integrated using CELLECT (CELL-type Expression-specific integration for Complex Traits). In CELLECT, MAGMA measures extent to which genetic associations with a phenotype increase as a function of gene expression specificity for a given cell type, categorized following nomenclature used in original Tabula Muris study.

Supplementary Table 15
Mendelian disease enrichment. To investigate the potential relationships of *mvAge* with Mendelian disease genes, using lead SNPs as input to MendelVar, implementing INRICH using the "target" enrichment mode and default setting for the target gene set filter and minimum observed threshold. Gene sets from the Human Disease Ontology and Human Phenotype Ontology databases included.

Supplementary Table 16
Mendelian phenotype ontology pathway enrichment. To investigate the potential relationships of *mvAge* with Mendelian associated pathways, using lead SNPs as input to MendelVar, implementing INRICH using the "target" enrichment mode and default setting for the target gene set filter and minimum observed threshold. Gene sets from the Human Disease Ontology and Human Phenotype Ontology databases included.

Supplementary Table 17
Mendelian randomization investigating the causal role of biomarkers, lifestyle risk factors, and diseases on *mvAge*.

Supplementary Table 18
Drug-target Mendelian randomization (MR) of metformin target genes on *mvAge*.

Supplementary Table 19

Drug-target Mendelian randomization (MR) for gene targets proxying lipid-modulating and blood pressure-lowering therapies.

Supplementary Table 20
Drug-target Mendelian randomization (MR) for gene targets proxying lipid-modulating and blood pressure-lowering therapies.

Supplementary Table 21
Colocalization results for scan of protein coding genes near glycated hemoglobin (HbA1c) lead SNPs on *mvAge.*

Supplementary Table 22
Colocalization results for scan of protein coding genes near high-density lipoprotein cholesterol (HDL-C) lead SNPs on *mvAge.*

Supplementary Table 23
Colocalization results for scan of protein coding genes near low-density lipoprotein cholesterol (LDL-C) lead SNPs on *mvAge.*

Supplementary Table 24
Colocalization results for scan of protein coding genes near triglycerides (TG) lead SNPs on *mvAge.*

Supplementary Table 25
Colocalization results for scan of protein coding genes near systolic blood pressure (SBP) lead SNPs on *mvAge.*

Supplementary Table 26
Significant cis-instrument scan results of protein coding genes within 50 kilobases of biomarkers glycated hemoglobin (HbA1c), high-density lipoprotein cholesterol (HDL-C), low-density lipoprotein cholesterol (LDL-C), triglycerides (TG), and systolic blood pressure (SBP). Top hits from cis-instrumentation scan of protein coding genes located near HbA1c, HDL-C, LDL-C, TG, and SBP, surpassing Bonferroni adjusted P-value threshold (354 genes tested across all protein coding genes). Annotated for druggability.

Supplementary Table 27
Cis-instrument scan of protein coding genes near glycated hemoglobin (HbA1c) lead SNPs. Taking forward HbA1c protein coding genes with evidence of shared causal variant between *mvAge* and HbA1c within the gene locus to cis-instrumentation for MR analysis.

Supplementary Table 28
Cis-instrument scan of protein coding genes near high-density lipoprotein cholesterol (HDL-C) lead SNPs. Taking forward HDL-C protein coding genes with evidence of shared causal variant between *mvAge* and HDL-C within the gene locus to cis-instrumentation for MR analysis.

Supplementary Table 29
Cis-instrument scan of protein coding genes near low-density lipoprotein cholesterol (LDL-C) lead SNPs (genes with evidence of shared causal variant with *mvAge*). Taking forward LDL-C protein coding genes with evidence of shared causal variant between *mvAge* and LDL-C within the gene locus to cis-instrumentation for MR analysis.

Supplementary Table 30
Cis-instrument scan of protein coding genes near triglycerides (TG) lead SNPs (genes with evidence of shared causal variant with *mvAge*). Taking forward TG protein coding genes with evidence of shared causal variant between *mvAge* and TG within the gene locus to cis-instrumentation for MR analysis.

Supplementary Table 31

Cis-instrument scan of protein coding genes near systolic blood pressure (SBP) lead SNPs (genes with evidence of shared causal variant with *mvAge*). Taking forward SBP protein coding genes with evidence of shared causal variant between *mvAge* and SBP within the gene locus to cis-instrumentation for MR analysis.

Supplementary Table 32
Replication of biomarker cis-instrument analysis. Using summary statistics from independent GWASs for glycated hemoglobin (HbA1c), high-density lipoprotein cholesterol (HDL-C), low-density lipoprotein cholesterol (LDL-C), and triglycerides (TG), genome wide significant cis-instruments were available for only 41 of the 132 protein coding genes previously identified.

Supplementary Table 33
Cis-Mendelian randomization protein coding genes drug-gene interaction information from the Drug-Gene Interaction database (DGIdb). Searching list of protein coding genes against a compendium of drug-gene interactions and potentially 'druggable' genes to assess potential therapeutic actionability.

Supplementary Table 34
STITCH protein-protein and protein-chemical interactions for genes identified in cis-instrument Mendelian randomization (MR) associated with *mvAge*. Assessing potential therapeutic actionability of identified protein coding genes, interactions explored with STITCH ('search tool for interactions of chemicals') interaction database.

Supplementary Table 35
Results of pQTL-based cis-instrument Mendelian randomization (MR) of 68 circulating proteins from SCALLOP consortia participants. Exploring causal role of circulating proteins from first results released by SCALLOP consortium, which used Olink platform to perform a protein quantitative trait loci (pQTLs) mapping of plasma proteins, to investigate potential anti-aging therapeutic opportunities.

Supplementary Table 36
Replication results for 6 of 8 top circulating proteins identified by SCALLOP consortia. Out of 8 proteins associated with *mvAge* previously identified using data from the SCALLOP consortia, cis-instruments were available for 6 in independent pQTL-based analyses of circulating proteins to implement an exploratory scan with *mvAge.*

Supplementary Table 37
Polygenic Mendelian randomization (MR) risk factors and biomarkers. Description and sources of risk factors and biomarkers used in Mendelian randomization analysis exploring causal role on *mvAge,* including but not limited to lipids, liver function enzymes, inflammation markers, glycemic markers, endocrine function markers, kidney function markers, substance use, sleep, well-being, education, physical activities, and leisure and social activities.

Supplementary Table 38
Metformin target genes. Primary metformin targets (AMPK, MCI, MG3, GSD15, and GLP1) were identified from literature. ChEMBL database used to identify genes related to mechanism of action for the 5.

Supplementary Table 39
Drug-target Mendelian randomization (MR) instruments for metformin targets extracted from GWAS data of glycated hemoglobin (HbA1c). With 5 primary metformin targets (AMPK, MCI, MG3, GSD15, and GLP1) identified from literature, ChEMBL database used to identify genes related to the mechanism of action for the 5. Variants within 100 kb of gene boundaries then extracted from GWAS of circulating HbA1c.

Supplementary Table 40
Antidiabetics drug target gene instruments. Genes encoding the antidiabetic drug targets for 6 classes of antidiabetic medications with known drug targets (beyond metformin) potentially suitable for genetic instruments were identified through lookup in DrugBank and ChEMBL databases. Variants within 100 kb of gene boundaries then extracted from GWAS of circulating HbA1c.

Supplementary Table 41

Lipid-modulating drug target gene instruments. GWAS summary statistics for circulating lipid levels were used to construct genetic instruments proxying pharmacological modulation of 6 lipid modulating drug classes and respective identified target genes within each class i.e., PCKK9, HMGCR, NPC1L1, ANGPTL3, ANGPTL4, CETP, LPL, LPA, APOC3, APOA1, PPARA, ABCG5/8, ANGPTL8, and ACLY. Variants within 100 kb of respective gene boundaries were selected as proxies for lipid modulation via these gene targets.

Supplementary Table 42
Instruments for antihypertensive target genes.
GWAS summary statistics for systolic blood pressure levels were used to construct genetic instruments proxying pharmacological modulation of 5 classes of antihypertensives i.e., ACE inhibitors, beta blockers, AGT inhibitors, thiazide diuretic, calcium channel blockers, and gene targets within each class, identified through lookup in DrugBank and ChEMBL databases. Variants within 100 kb of respective gene boundaries were selected as proxies for pharmacologically lowering blood pressure.

Supplementary Table 43
Protein coding genes within 50 kilobases of glycated hemoglobin (HbA1c) lead SNPs. BioMart used to identify and curate protein-coding genes located within 50 kilobases of HbA1c lead variants identified previously in polygenic and drug-target MR, with resultant genes systematically taken forward.

Supplementary Table 44
Protein coding genes within 50 kilobases of high-density lipoprotein cholesterol (HDL-C) lead SNPs. BioMart used to identify and curate protein-coding genes located within 50 kilobases of HDL-C lead variants identified previously in polygenic and drug-target MR, with resultant genes systematically taken forward.

Supplementary Table 45
Protein coding genes within 50 kilobases of low-density lipoprotein cholesterol (LDL-C) lead SNPs. BioMart used to identify and curate protein-coding genes located within 50 kilobases of LDL-C lead variants identified previously in polygenic and drug-target MR, with resultant genes systematically taken forward.

Supplementary Table 46
Protein coding genes within 50 kilobases of triglycerides (TG) lead SNPs. BioMart used to identify and curate protein-coding genes located within 50 kilobases of TG lead variants identified previously in polygenic and drug-target MR, with resultant genes systematically taken forward.

Supplementary Table 47
Protein coding genes within 50 kilobases of systolic blood pressure (SBP) lead SNPs. BioMart used to identify and curate protein-coding genes located within 50 kilobases of SBP lead variants identified previously in polygenic and drug-target MR, with resultant genes systematically taken forward.

Supplementary Table 48
Druggable genes. To evaluate potential therapeutic actionability of top protein-coding genes, assessed druggable genes as defined by Finan et al. and Gaziano et al.

Supplementary Table 49
Instruments for 68 circulating proteins from the SCALLOP Consortia. Investigating potential anti-aging therapeutic opportunities of circulating proteins using first results from SCALLOP (Systematic and Combined AnaLysis of Olink Proteins) consortia, which employed Olink Proteomics platform to perform protein quantitative trait loci (pQTLs) mapping of plasma proteins. Selected pQTLs instruments associated with plasma protein at genome wide significance within cis-acting loci of target gene boundaries (±100 kilobases); 68 pQTLs retrieved for exploratory scan with *mvAge*.

Supplementary Table 50
Instruments for 6 of 8 circulating proteins from pQTL-based drug target MR scan from 68 circulating proteins among participants in the SCALLOP Consortium. Replicating findings of proteins surpassing the adjusted threshold using additional pQTL-based analysis of circulating proteins; out of 8 proteins associated with

mvAge identified in SCALLOP, 6 available in independent pQTL GWAS at genome wide significance within cis-acting loci of target gene boundaries(±100 kilobases) for exploratory scan with mvAge.

SUPPLEMENTARY RESULTS

Mendelian randomization of modifiable risk factors.

Among modifiable risk factors, we observed MR estimates for adverse associations related to smoking behaviors, e.g., cigarettes smoked per day (Beta=-0.038, P-value=3.1×10^{-10}). Conversely, we did not find evidence of an association with alcohol consumption (Beta=-0.001, P-value=0.941). In addition, several endpoints related to sleeping behavior also demonstrated associations, e.g., increased self-reported genetic liability for insomnia was adversely associated (Beta=-0.154, P-value=2.24×10^{-11}), while increased sleep duration was beneficially associated with *mvAge* (Beta=0.060, P-value=1.55×10^{-4}). Finally, we found that usual walking pace was associated with increased *mvAge* (Beta=0.258, P-value=9.52×10^{-24}). MR estimates for other measures of physical activity suggested beneficial relationships with *mvAge* (P-value < 0.05), including the genetic predisposition for increased vigorous physical activity (Beta=0.146, P-value=0.003). Finally, there was some genetic evidence for participation in leisure/social activities being beneficial for *mvAge* with self-reported participation in religious groups having a positive MR estimate on *mvAge* (Beta=0.258, P-value=1.93×10^{-4}). Full risk factor results are reported in **Supplementary Table 17**.

Among biomarkers, we found relationships primarily with HbA1c, circulating lipids, and blood pressure: higher levels of several lipid subfractions (per SD increase mmol/L) were linked with reduced *mvAge*, including, low-density lipoprotein cholesterol (LDL-C) (P-value=4.1×10^{-47}), triglycerides (P-value=5.1×10^{-11}), and lipoprotein a Lp(a) (P-value=6.9×10^{-12}). Increased systolic blood pressure (SBP) (per SD increase in mmHg) was adversely associated with *mvAge* (P-value=1.6×10^{-106}). We also observed adverse findings with liver function enzymes (per SD increase in quantile U/L). For example, higher levels of alanine aminotransferase (ALT), aspartate aminotransferase (AST), and gamma glutamyltransferase (GGT) were each linked with reduced *mvAge* (ALT P-value=1.6×10^{-11}; AST P-value=3.6×10^{-4}; GGT P-value=4.8×10^{-8}).

Drug-target MR using pQTLs identifies CSF-1, MMP-1, and IL-6RA.

Finally, we aimed to identify additional protein targets associated with *mvAge* that may help future studies into anti-aging therapeutics. Of the 68 cis-instrumented circulating proteins derived from approximately 30,000 participants,[1] we identified 8 proteins with effect estimates surpassing correction for multiple testing (**Extended Data Fig. 4, Supplementary Table 35**): Colony Stimulating Factor 1 (CSF-1) (Beta=-0.013, P-value=2.67×10^{-4}); cystatin B (CSTB) (Beta=-0.005, 95% CI, -0.00165, -0.002, P-value=$6.45\text{x}10^{-4}$); C-X3-C Motif Chemokine Ligand 1 (CXC3L1) (Beta=-0.006, P-value=2.49×10^{-5}); Interleukin 6 Receptor Subunit Alpha (IL6-RA) (Beta=0.004, P-value=4.64×10^{-22}); Matrix Metallopeptidase 1 (MMP-1) (Beta=-0.003, P-value=4.5×10^{-6}); Matrix Metallopeptidase 10 (MMP-10) (Beta=0.006, P-value=5.25×10^{-5}); Natiuretic Peptide Precursor B (NPPB) (Beta=0.0122, P-value=1.72×10^{-4}); and Advanced Glycosylation End-Product Specific Receptor (RAGE) (Beta=0.084, P-value=1.08×10^{-14}). For IL-6RA, it is of note that the corresponding effect estimate for IL-6 was null (P-value=0.952). Of the 6 circulating proteins we were able to cis-instrument in additional pQTL datasets, CSF-1, IL-6RA, and MMP-1 successfully replicated, demonstrating directionally consistent MR estimates with the estimates SCALLOP Consortia findings: CSF Beta=-0.011 (P-value=2.53×10^{-4}); IL-6RA Beta=0.003 (P-value=9.42×10^{-7}), and MMP-1 Beta=-0.003 (P-value=6.92×10^{-3}) (**Supplementary Table 36**).

SUPPLEMENTARY DISCUSSION

mvAge units in relation to metformin targets.

Causal inference requires triangulating study designs.[2] Our results generally align with preclinical findings studying metformin. For example, in *C. elegans*, metformin has

reported increases in average lifespan by up to 40% (no overall increase in lifespan was observed, however[3]). This estimate was concordant with results showing increased lifespan by up to 36% in fruit flies without corresponding changes in survival.[4] In middle-aged mice, a low dose metformin administration increased lifespan by about 5.8%. It also increased the healthspan of the middle aged mice (measured by increased physical fitness).[5]

As discussed in the main manuscript, in addition to *mvAge* being a composite representing the broad genetic liability of aging-related traits that include binary and continuous units, the MR estimates reflecting exposure over the lifetime are generally challenging to interpret clinically, especially as it relates to assessing shorter-term exposure (such as therapeutics).[6,7] However, we observed corresponding evidence of relationships between the metformin targets and the univariate age-related GWAS that have more units that are more easily interpreted. For the univariate GWAS analyses of the metformin target genes, we found that the metformin targets approximately doubled the likelihood of being in the 90th percentile (compared to the 60th percentile) in the exceptional longevity GWAS (**Supplementary Table 19**). It also increased healthspan (beta=0.36 in SD of healthspan years per 1 SD decrease in HbA1c [mmol/mol]), suggesting potentially clinically meaningful reductions in HbA1c via the target genes.

Notably, in the pre-clinical studies, there is also some evidence of toxicity of metformin at higher dosages, suggesting a dose-dependent effect,[8] which is also difficult to assess in MR designs because they assume a linear relationship between the exposure and outcome.[6] Further, while current human data on the impact of metformin on aging is limited, several observational studies have shown clinically meaningful, beneficial relationships with non-T2D diseases. For example, case-control studies showed metformin use reduces cancer among those with T2D.[9] It has been suggested that the metformin-cancer relationship is different than other antidiabetic therapeutics with one retrospective analysis finding that metformin use lowered incidence of cancer by approximately 37% over the 11 year study period.[10] A similar nationwide case-control study of 97,430 hepatocellular carcinoma patients and 19,860 matched controls found that each additional year of metformin use reduced the risk of a hepatocellular carcinoma diagnosis by about 7%,[11] suggesting a potential protective effect. However, these and other observational studies of metformin and cancer risk also highlight potential time-window and time-lagging biases that may inflate the estimates,[12] further highlighting the importance of genetics-based work.

In sum, while there are many animal studies finding benefits of metformin on aging and other disease across multiple model organisms, a growing body of literature finding, especially among T2D patients, metformin has clinically meaningful, beneficial relationships with non-T2D diseases, including cancer and cardiovascular disease, and ongoing clinical studies evaluating the impact of metformin on aging,[13,14] current human evidence is limited. Therefore, in addition to the need for and improved understanding of the biological pathways underlying the relationships of metformin and aging, future studies will be needed to further characterize the magnitude, and dose-dependent relationships of metformin and aging.

MR of lifestyle risk factors and biomarkers.

MR analyses of modifiable lifestyle risk factors and biomarkers, in addition to implicating several lipid subclasses, support and extend previous work linking poor liver function (as indicated by elevated liver function enzymes),[15] apolipoprotein B,[16] and smoking[17,18] with reduced longevity and educational attainment with increased longevity.[19,20] There may be several pathways through which their impact may be mediated. For example, the impact of smoking on aging likely acts through its role in lung cancer, cardiovascular diseases,[21] and while educational attainment has been linked with reduced risk for many chronic physical diseases,[22] recent genetics-based analysis has shown a relationship of educational attainment with suicide behaviors,[23] so the beneficial role of education may, in part, be mediated via a reduction in suicides. Notably, a longitudinal study following more than 400,000 adults over 24 years showed that elevated AST and ALT levels (≥ 40 IU/L) reduced life expectancy (including both liver-related and other causes of death, such as stroke, cancer, etc.) by approximately 10 years.[15] It has also recently been shown that PCSK9 inhibition with monoclonal antibodies attenuated ALT in rat models of alcohol-induced hepatic steatosis,[24] suggesting an additional mechanism through which PCSK9 may impact aging. Further, given the relationship of alcohol consumption and liver function,[25] these findings suggest that while we did not find MR evidence of alcohol consumption and longevity, it is plausible that if alcohol consumption adversely impacts liver function and liver function adversely impacts longevity, there may be an impact of alcohol consumption and longevity that is not captured by the genetics-based analysis. In addition, our apolipoprotein B findings further highlight an emerging role as an important lipoprotein subfraction in health and disease beyond cardiovascular endpoints[16] and support work suggesting that future

cardiovascular drug development would benefit from targeted reduction in the concentrations of atherogenic lipoproteins compared to the reduction in cholesterol or triglycerides carried by these lipoproteins,[26] which would then also have important implications for improving healthy aging.

pQTL-based cis-instrument MR highlights potential circulating proteins as mediators of aging.

Our exploratory pQTL-based MR using the largest available pQTL data[1] should aid future research into therapies to improve healthy aging because most approved pharmacotherapies target proteins.[7,27] We identified and replicated in independent pQTL datasets three circulating proteins, CSF-1, MMP-1, and IL-6RA, finding increased circulating levels of CSF-1 and MMP-1 adversely impacted aging. CSF-1 is a growth factor released by osteoblasts in bone marrow, contributing to the proliferation, differentiation, and survival of several immune cell types, including monocytes, macrophages, and bone marrow progenitor cells.[28] Abnormal CSF-1 signaling has been implicated in the development of atherosclerotic lesions,[29] and immunologic and hematologic abnormalities in chronic kidney failure patients.[30] There have been Phase 1 and 2 clinical trials for two monoclonal antibody CSF-1 inhibitors (PD-0360324 and MCS110) for several cancer types[31,32] and rheumatoid arthritis,[33] that have suggested the therapeutics to be well-tolerated with minimal adverse events and supported the additional clinical study of these CSF-1 inhibitor.[31-33] MMP-1 is one of several collagenases that degrade interstitial collagens (types 1,2, and 3) comprising extracellular matrices and play important roles in tissue remodeling.[34] Upregulated MMP-1 has been shown to be involved in several pathological processes, including angiogenesis, cirrhosis, arthritis, and metastasis.[35] Recent work has also implicated excess MMP-1 in the development of aortic aneurysm through degradation of structural proteins comprising the aortic wall.[36] First generation, non-specific MMP inhibitors largely failed in cancer clinical trials due to poor efficacy (including conflicting roles of MMPs in tumor progression and suppression);[37] however, recent progress in developing highly specific MMP inhibitors has re-invigorated investigation into the therapeutic application of MMP inhibitors[38] and our findings suggest follow-up studies into their role in aging may be warranted. Circulating IL-6RA levels were associated with healthier aging, an apparent protective role in line with previous MR work evaluating the impact of IL-6RA on coronary artery disease.[1] We did not find an association, however, with IL-6 (IL-6RA's ligand[39]), an unexpected result in light of the well-known role of IL-6 in pro-inflammatory conditions.[40] Further, IL-6 signaling plays an important role in the normal metabolic processes, neural development, and several other protective and regenerative functions.[39] IL-6 signaling proceeds via either cis or trans (which mediates the pro-inflammatory impact of IL-6) signaling cascades[39] and it has been found that selective blocking of the trans signaling cascade (while leaving cis signaling intact) improves patient outcomes in models of bone healing and sepsis,[39] which suggests a complex role of IL-6 and IL-6RA in health and disease.[39]

SUPPLEMENTARY METHODS

Genomic structural equation modeling (Genomic-SEM) background and overview.

Genomic structural equation modeling (Genomic-SEM) is a recently developed method that uses GWAS summary statistics of related complex traits to model their shared genetic architecture.[41] It is a two-stage application of structural equation modeling (SEM) (i.e., methods that examine the variance and covariance structure among groups of related variables) to GWAS studies.[42-44] Genomic-SEM has been shown to be robust to both differences in sample-sizes of the input GWAS and sample overlap of the input GWASs, enhancing applicability and facilitating the capability to improve statistical power from increased effective sample sizes.[41] Further, in contrast to other GWAS meta-analysis methods (e.g., MTAG[45]), we used Genomic-SEM to first model the joint genetic architecture of aging, jointly analyzing GWASs from five genetically correlated aging-related phenotypes; and then generate a GWAS, identifying individual SNP associations for this general latent aging factor. Below we provide the relevant principles and details of SEM in the context of Genomic-SEM based upon the original Genomic-SEM methods paper (Grotzinger et al. 2019),[41] using their mathematical notation throughout our summary.

Structured covariance models and factor analysis.

Genomic-SEM allows the user to specify the best model fit for the SEM. SEMs are apportioned into two equations – the measurement model and the structural model.[41] The measurement model is used during factor analysis (FA) and models the variance and covariance sets of the indicator/observed variables through their relationships with the latent factor(s) (FA models the variance

shared across the indicator/observed variables).[41] In the measurement model, denoted $\boldsymbol{y} = \boldsymbol{\Lambda\eta} + \boldsymbol{\mathcal{E}}$, k indicator (i.e., observed) phenotypes are modeled as a linear function of a smaller set of m latent variables. In the above equation, $\boldsymbol{y}$ is a $k \times 1$ vector of indicator/observed variables, $\boldsymbol{\mathcal{E}}$ is a $k \times 1$ vector of indicator/observed residuals, $\boldsymbol{\eta}$ is a $m \times 1$ vector of latent variables, and $\boldsymbol{\Lambda}$ is a $k \times m$ matrix of regression coefficients for the latent factors on the indicator/observed variables.[41] We used Genomic-SEM to model each indicator/observed aging-related GWAS as a function of one latent variable.[41]

In SEM, structural models relate the latent factors to each other by their directed regression coefficients, which may be represented by the equation, $\boldsymbol{\eta} = \mathrm{B}\boldsymbol{\eta} + \boldsymbol{\zeta}$.[41] In the structural model, B is an $m \times m$ matrix of the regression coefficients relating the latent variables to each other. $\boldsymbol{\zeta}$ is an $m \times 1$ matrix vector of the latent residuals. The model-implied covariance matrix of observed variables is $\sum(\theta) = \Lambda(I - B)^{-1}\,\Psi[(I - B)^{-1}]'\,\Lambda' + \Theta$ with I equal to a $k \times k$ identity matrix.[41] Full SEM models use sets of linear equations to represent the empirical matrix by parameters relating observed variables to the latent variables and the latent variables to each other.[41]

Genomic-SEM leverages the measurement and structural model framework to model the genetic covariances across indicator/observed GWAS phenotypes of interest. In stage 1, Genomic-SEM estimates the empirical genetic covariance matrix sampling covariance matrix using the LDSC package, modified to account for possible sample overlap.[41] In stage 2, a multivariate regression and covariance system between the observed phenotypes and the latent factor(s) (Genomic-SEM can model more than one latent factor) is specified; then the parameters (θ) that minimize the discrepancy of the fit function between the model's implied covariance matrix $(\sum \theta)$ and the corresponding empirical covariance matrix, "*S*", estimated.[41]

In stage 1, Genomic-SEM uses a multivariable version of cross-trait linkage disequilibrium score regression (LDSC)[46] to estimate the genetic covariance matrix:

$$S_{\mathrm{LDSC}} = \begin{bmatrix} h_1^2 & & & \\ \sigma_{\mathrm{g1,g2}} & h_2^2 & & \\ \vdots & & \ddots & \\ \sigma_{\mathrm{g1,g}k} & \sigma_{\mathrm{g2,g}k} & \cdots & h_k^2 \end{bmatrix}$$

(*k* is number of observed phenotypes – *k* = 5 in our study). The diagonal elements are heritabilities for the common genetic variants. The off-diagonal elements are the genetic covariances between the observed phenotypes. Unbiased estimates and standard errors are obtained by using the nonredundant elements in the S matrix to create an asymptotic sampling covariance matrix, "V_{SLDSC}", comprised of the LDSC regression estimates. V_{SLDSC} is symmetric (order k*); the diagonal elements and off-diagonal elements are the sampling variances and covariances, respectively.[41] The V_{SLDSC} matrix is written as:

$$V_{S_{\mathrm{LDSC}}} = \begin{bmatrix} \mathrm{s.e.}(h_1^2)^2 \\ \mathrm{cov}(h_1^2, \sigma_{g1,g2}) & \mathrm{s.e.}(\sigma_{g1,g2})^2 \\ \vdots & \vdots & \ddots \\ \mathrm{cov}(h_1^2, \sigma_{g1,gk}) & \mathrm{cov}(\sigma_{g1,g2}, \sigma_{g1,gk}) & & \mathrm{s.e.}(\sigma_{g1,gk})^2 \\ \vdots & \vdots & & \vdots & \ddots \\ \mathrm{cov}(h_1^2, h_j^2) & \mathrm{cov}(\sigma_{g1,g2}, h_j^2) & & \mathrm{cov}(\sigma_{g1,gk}, h_j^2) & & \mathrm{s.e.}(h_j^2)^2 \\ \vdots & \vdots & & \vdots \\ \mathrm{cov}(h_1^2, \sigma_{gj,gk}) & \mathrm{cov}(\sigma_{g1,g2}, \sigma_{gj,gk}) & & \mathrm{cov}(\sigma_{g1,gk}, \sigma_{gj,gk}) & & \mathrm{cov}(h_j^2, \sigma_{gj,gk}) \\ \mathrm{cov}(h_1^2, h_k^2) & \mathrm{cov}(\sigma_{g1,g2}, h_k^2) & & \mathrm{cov}(\sigma_{g1,gk}, h_k^2) & & \mathrm{cov}(h_j^2, h_k^2) \end{bmatrix}$$

Diagonal elements in V_{SLDSC} are estimated using a jackknife resampling procedure from the LDSC package[46] extended in the Genomic-SEM package.[41] Next the effects of the individual SNPs are incorporated: first, the initial input genetic covariance matrix is extended to model covariances between individuals SNPs and each observed phenotype by incorporating a vector of SNP-phenotype covariances, denoted "S_{SNP}" to S_{LDSC}:

$$S_{\mathrm{Full}} = \begin{bmatrix} \sigma^2_{\mathrm{SNP}} \\ \sigma_{\mathrm{SNP},g1} & h_1^2 \\ \sigma_{\mathrm{SNP},g2} & \sigma_{g1,g2} & h_2^2 \\ \sigma_{\mathrm{SNP},g3} & \sigma_{g1,g3} & \sigma_{g2,g3} & h_3^2 \\ \vdots & \vdots & & & \ddots \\ \sigma_{\mathrm{SNP},gk} & \sigma_{g1,gk} & \sigma_{g2,gk} & \sigma_{g3,gk} & \cdots & h_k^2 \end{bmatrix}$$

The sampling covariance matrix, "V_{SFULL}", that is associated with S_{FULL} is written:

$$V_{S_{\mathrm{Full}}} = \begin{bmatrix} V_{S_{\mathrm{SNP}}} \\ 0 & V_{S_{\mathrm{LDSC}}} \end{bmatrix}$$

V_{SFULL} has one block, V_{SLDSC}, which includes the SNP heritabilities (i.e., sampling variances and covariances of the latent genetic variances) and the genetic covariances estimated by the multivariable LDSC analysis. The other V_{SFULL} block includes the "V_{SSNP}" matrix comprised of the SNP effects-phenotype sampling covariance matrix. We use the 1000 Genomes Phase 3 European reference panel for SNP variance (considered fixed). The SNP sample variance and sampling covariance are then fixed to 0. Next, the sampling covariances of the SNP genotype covariances are constructed from cross-trait multivariate LDSC intercepts. The final V_{SFULL} block corresponds to the sampling covariance of the SNP genotype covariances with the genetic variances and genetic covariances,[41] which are fixed at 0: the SNP genotype covariance is expected/assumed to be independent of the test statistics of other LD blocks (other than the LD block where those SNPs are located). Finally, the sampling variance of the heritabilities and genetic correlations are derived from sampling variability in the test statistics from *all* of the LD blocks: their sampling covariances with individuals SNP is also expected to be zero.[41]

In stage 2, parameters of the user-specified SEM model are estimated with either weighted least squares (WLS) or maximum likelihood (ML) estimators using the S_{LDSC} matrix (from stage 1). WLS and ML estimators weigh the matrix information differently, but both estimators minimize the fit error between the model-implied and empirical

genetic covariances.[41] We use the WLS estimator as recommended and used in recent Genomic-SEM GWAS studies.[47,48] WLS optimizes the fit function by using the diagonal elements in the V_{SLDSC} matrix and adjusting standard errors of the estimates with the off-diagonal elements, indices for the correlations among the sampling errors of the summary statistics.[41] These features make Genomic-SEM unbiased and robust up to 100% sample overlap and also unbalanced GWAS sample sizes.

Assessing model fit.

We evaluated model fit using conventional SEM indices, including the model χ^2 statistics, the Akaike information criterion (AIC), comparative fit index (CFI), and the standardized root mean square residual (SRMR). Apart from the χ^2 statistic, each index uses its standard SEM interpretation.[41] Large sample sizes common in GWAS analysis may overpower the χ^2 test, increasing the likelihood of it being statistically significant.[41] As suggested by the developers of Genomic-SEM, we used the model χ^2 statistic as comparative measure of fit , not as a measure of statistical significance. Following guidelines in use,[49] we considered CFI and SRMR values of > 0.90 and < 0.08 as indicating good fit.[49]

Confirmatory factor analysis.

Confirmatory factor analysis (CFA) is used in SEM to evaluate the strength of the relationships between the latent general factors of aging and the indicator/observed variables,[41] and as well to evaluate the model fit.[41] CFA confirmed that our primary common factor *mvAge* latent factor was a good fit of the age-related GWAS data used as indicator/observed variables: the *mvAge* CFI was 0.968 and the SRMR was 0.069 (**Supplementary Table 4, Panel A**).

Effective sample size calculation.

The Genomic SEM method is robust to sample overlap,[41] making it applicable to our analyses given the overlap across cohorts included in the five aging-related GWASs included (UK Biobank cohorts were included in the parental lifespan, healthspan, and frailty GWASs, and other smaller cohorts were potentially included in more than one GWAS used in our analysis (**Supplementary Table 1,** note)). We estimated the effective sample sizes for each SNP included in the *mvAge* GWAS using steps prescribed previously.[47,48] Given the multivariate GWAS effect estimate for SNP *j,*

$$\beta_j = \frac{Z_j}{\sqrt{n_j \times 2 \times MAF_j(1 - MAF_j)}}$$

(where Z_j is the multivariate GWAS association statistic for SNP *j*, n_j is the effective sample size for SNP *j* that we aim to calculate, MAF_j is the minor allele frequency of SNP *j*; the SNP *j* variance (σ_j^2) is $2 \times MAF_j(1 - MAF_j)$), and rearranging,

$$n_j = \frac{(Z_j/\beta_j)^2}{\sigma_j^2}.$$

Effective sample size N_{eff} is taken to be approximately equal to the mean n_j for m SNPs meeting the MAF thresholds (restricted in our analyses, as recommended, to MAF between 10% and 40% because the effective calculations are inflated):

$$N_{eff} \approx \frac{1}{m}\sum_{MAF=a}^{b} n_j.$$

Heterogeneity testing (QSNP).

A strength of the Genomic-SEM pipeline is the ability to assess whether the multivariate GWAS SNP associations are explained best by a shared causal pathways (i.e., operating on the observed GWASs via the shared latent factor), or trait-specific pathways independent of the shared multivariate, latent GWAS.[41] SNP-level heterogeneity test statistics (Q_{SNP}) are estimated for each lead *mvAge* SNP (independent SNP associated with *mvAge* with P-value $< 5\times10^{-8}$). The Q_{SNP} is a χ^2-distributed statistic, the null hypothesis, namely, that the SNP effect is *completely* mediated by a common pathway (i.e., *mvAge* factor). The test is two-sided. A statistically significant Q_{SNP} suggests that the SNP effect is best explained by specific pathways that are independent of the shared *mvAge* factor.[41] Based upon previous multivariate GWASs generated using Genomic-SEM,[48,50] we evaluated Q_{SNP} heterogeneity using a Bonferroni adjusted P-value threshold of 9.62×10^{-4} (0.05/52 lead SNPs). Out of 52 lead SNPs, 9 were heterogeneous, suggesting that the majority of the *mvAge* lead SNPs

associations explained best by a shared causal pathway. Among the *mvAge* lead variants with statistically significant Q_{SNP} statistics (P-value < 9.62×10^{-4}), the exonic rs1572849 (nearest gene: *TOMM40*) had the strongest evidence that its association is explained by specific, independent pathways (P-value < 3.44×10^{-27}) (**Supplementary Table 6**). Interestingly, the two variants, rs405509 and rs7412 that are near APOE were among the variants with statistically significant Q_{SNP} values (**Supplementary Table 6**). The T allele for the exonic rs7412 variant, also known as Arg176Cys, is known to indicate the presence of the Apoε2.[51] Apoε2 is one of the three common isoforms ($\varepsilon2, \varepsilon3, \varepsilon4$) that are products of combinations of rs7412 and another common variant, rs429358.[51] Apoε4 is the isoform associated with increased Alzheimer's disease (AD) and AD pathogenesis.[52] By contrast, Apoε2 is known to be protective – it reduces the risk of late-onset AD compared to the other isoforms[53] – and is considered a potential AD drug target.[53] Given the strong relationships with AD, rs7412 is undoubtedly involved in age-related diseases, but the Q_{SNP} suggests that it is not involved in our more general age-related multivariate *mvAge* genetic signature, possibly because it is primarily involved in AD and related pathology.

Exploratory two-factor model analysis with Genomic-SEM to assess the genetic relationship between healthy aging and longevity.

The relationship between life expectancy and healthy aging has important implications for public health intervention/prevention strategies and drug discovery and repurposing endeavors.[54] For example, there have been increases in life expectancy in the last ~70 years (from 47 to 73 years),[54] which have resulted in a demographic increase in the population proportion of those >70 years of age with a coincident increase in the years of poor health and high morbidity experienced by many, especially near the end of life.[55] This life expectancy-healthy aging gap is currently approximately 9 years (i.e., a 9 year difference between life expectancy and the number of disease-free years lived),[55] and has prompted the World Health Organization to recently propose that there needs to be a shift in healthcare models focused on improving healthy aging to relieve the socioeconomic and public health burden related to a demographic shift towards older populations with increased numbers of individuals living with functional losses related to chronic diseases and ongoing health issues.[56,57]

Given our aging-related GWAS data encompass aspects of life expectancy (parental lifespans and extreme longevity), biological aging (epigenetic age acceleration), and healthy aging (healthspan and frailty), we sought to investigate whether we could assess the genetic relationships linking life expectancy/lifespan and healthy aging using Genomic-SEM and evaluate the extent to which our primary *mvAge* multivariate GWAS captures aspects encompassing life expectancy and healthy aging. We used Genomic-SEM's exploratory factor analysis (EFA) to guide our specification of the more nuanced model (**Supplementary Table 3**). Based on the EFA results, we ran a follow up CFA analysis (**Supplementary Table 4**). The two-factor solution provided good fit to the data (CFI=0.996, SRMR=0.035), with factor loadings suggesting one latent factor comprised of life expectancy-related GWASs (parental lifespan, extreme longevity, and epigenetic age acceleration) and the other latent factor comprised of the healthy aging-related GWASs (i.e., healthspan and frailty). A strong genetic correlation between these two factors supports our exploratory hypothesis of shared, but distinct, components of life expectancy/lifespan and healthy aging, and at the same time, indicates that our general shared factor – *mvAge* - captures both of these aspects of aging.

Fine-mapping.

We performed fine-mapping to identify the most plausible causal variants in the genomic loci associated with *mvAge*. We used two fine mapping methods – SuSIE[58,59] (applied to summary statistics[59]) and FINEMAP[60] – implemented in the R package, echolocatoR (version 2.0.3).[61] echolocatoR automates fine mapping of GWAS summary statistics and facilitates comparison of nominated putatively causal variants across fine-mapping methods, which is important as each fine mapping method with complementary strengths and weakenesses.[61] High linkage disequilibrium (LD) between genetic variants makes identifying true causal variants challenging[59] and SuSIE ("SUm of SIngle Effects") extends Bayesian variable selection in regression (BVSR) applied to fine-mapping by applying an iterative Bayesian stepwise selection (IBSS) process (in contrast with a traditional stepwise selection method) and producing credible sets of variants that quantify the uncertainty regarding the selection of a particular variant among the set of highly correlated variants.[58,59]

By contrast, FINEMAP leverages a Shotgun Stochastic Search (SSS) algorithm, which is based upon the Markov Chain Monte Carlo (MCMC) algorithms used in applications of Bayesian inference,[60] and for each locus with p SNPs (and a corresponding 2^p possible causal models),[62] efficiently evaluates neighboring models at each iteration

(compared to search strategies employed by CAVIAR and other early fine-mapping methods[62]). Because fine-mapping in summary statistics needs a high-quality correlation estimate ideally computed using the same genotype data as the effect estimates (i.e., SNP association statistics),[60] we used the same 1000 Genomes Phase 3 reference panel that was used to generate the *mvAge* summary data in Genomic-SEM. SuSIE and FINEMAP methods each generate a posterior probability that the variant is causal for *mvAge*. We used a 250 kilobase window ($\pm$250 kilobases around the lead SNP [SNP with smallest P-value in the locus]) and a stringent probability threshold of 0.95 to define credible sets of potentially causal variants. echolocatoR defines a "consensus SNP" as a variant included in both SuSIE and FINEMAP,[61] calculates an average posterior probability ("mean.pp"), and determines an average credibility set ("mean.cs"), which is set to 1 when the mean SNP-wise posterior probability across SuSIE and FINEMAP exceed 0.95 and 0 otherwise.[61]

Gene prioritization using transcriptomic imputation.

We next performed a transcriptome-wide association study (TWAS) to further investigate gene-level associations of the *mvAge* genetic signature using the TWAS FUSION method and followed the FUSION protocol default settings on autosomal chromosomes.[63] For our TWAS, we downloaded 37,920 pre-computed expression quantitative trait loci (eQTL) features from GTEx Version 8 from the FUSION website[63] that leverage cross-tissue sparse canonical correlation (sCCA) models that integrate genetic features across multiple tissues to increase TWAS power (especially when sample sizes for individual tissues used to derive the TWAS features are small), reduce testing burden, and when the disease-relevant tissue is not known.[63,64] We also used the 1000 Genomes Project Phase 3 European subpopulation for LD estimation (per FUSION protocol).[63] We excluded genes located within the major histocompatibility complex (MHC) (chromosome 6: given the complex LD structure of that region). After munging the *mvAge* summary statistics, there were 36,149 of the 37,920 sCCA features available in the *mvAge* data for analysis. The primary FUSION TWAS pipeline constitutes three steps: (1) identify gene expression features that are cis-heritable (i.e., variants associated with gene expression within or near the genomic locus); (2) construct a linear predictor for each cis-heritable gene (i.e., a SNP-based prediction weight of the gene feature); and (3) calculate both TWAS test-statistics incorporating these SNP-based prediction weights and summary-level GWAS Z-scores.[63] FUSION uses penalized several linear regression and Bayesian sparse linear mixed models (e.g., GBLUP, LASSO, Elastic Net, BLSMM) and computes an out-of-sample R^2 statistics to identify the best model via a cross-validation of each gene-GWAS model and we used Bonferroni corrected-statistical thresholds for determined by the number of genes tested across panels: $P < 1.383\times10^{-6}$ (0.05/36,149 sCCA features available for testing in *mvAge*) as a heuristic to facilitate our follow-up analysis on a plausible number of TWAS findings.

We took each of the TWAS genes associated with *mvAge* surpassing Bonferroni correction for multiple comparisons forward for additional analysis. Colocalization uses a Bayesian approach to estimate posterior probabilities (PP) for SNP associations between two outcome (here *mvAge* and the TWAS gene expression) at distinct loci are driven by a shared causal SNP,[65] which facilitates determination of whether the observed associations are driven by horizontal pleiotropy, i.e., a single SNP impacting both gene expression and *mvAge* (posterior probability PP.H4) or linkage disequilibrium (LD), i.e., 2 separate SNPs in LD impacting gene expression and GWAS signal separately (posterior probability PP.H3).[65] We evaluated colocalization between *mvAge*-associated gene (P-value $< 1.38\times10^{-6}$) using all variants within a $\pm$500 kb window surrounding the lead variant of the gene and performed colocalization using the coloc R package.[65] We categorized results using a PP.H4 threshold of > 0.6, which suggests evidence for a shared causal variant between eQTL and the GWAS signal at that genomic locus.[65]

We also applied FOCUS (Fine-mapping Of CaUsal gene setS), a fine-mapping method analogous to fine-mapping of GWAS results, using the same sCCA weights to identify potentially causal gene features within TWAS-significant regions.[66] For each genetic region containing a TWAS-significant association, FOCUS controls for potential pleiotropic SNP effects and calculates the posterior inclusion probability (PIP) of each feature by summing the posterior probabilities so as to define a set of features with a strong likelihood of containing a causal feature (i.e., a 95% credible set).[66] In FOCUS, individual feature PIP values > 0.5 are considered to suggest that the feature is more likely to be causal than any other regional feature.[66]

We prioritized "high-confidence" *mvAge* genes identified by FUSION based upon additional evidence for colocalization and fine-mapping. Along the lines of previous work,[67] we considered TWAS-significant genes associated with *mvAge* that also colocalized (PP.H4 > 0.6) and are likely to be causal (PIP > 0.5). For high-

confidence genes with associations across more than one sCCA panel, we filtered results for the gene/sCCA combination based upon variance explained by the gene/sCCA pair.

Gene set enrichment.

We used MAGMA[68] (Multi-marker Analysis of GenoMic Annotation) with data from GTEx (version 8) to perform gene-based and gene-set analyses MAGMA gene-based analyses included mapping SNPs to 18,649 protein coding genes within 10 kilobases of lead SNPs using several regression methods to account for LD between SNPs (using the1000 Genomes European (EUR) sample as the reference).[68] We used a Bonferroni-corrected threshold of P-value < 2.68×10^{-6} to account for multiple coding genes comparisons. We also performed FUMA GENE2FUNC ("gene to function") gene-set analyses using genes identified with MAGMA to evaluate potential relationships between *mvAge* and lists of mapped genes from MSigDB gene sets, i.e., Reactome, and Gene Ontology (GO)).[69] We used an FDR 5% threshold correction to account for multiple testing to identify gene-sets enriched with *mvAge*.

Disease and Phenotype Ontology Enrichment.

We investigated the potential relationships of *mvAge* with Mendelian disease genes and associated pathways with MendelVar.[70] We used our lead SNPs as input to MendelVar and performed analyses using intervals based on a ±0.5 Mb window around the lead SNPs with reference panels derived from the 1000 Genomes EUR population. Within MendelVar, we ran INRICH using the "target" enrichment mode and the default setting for the target gene set filter and minimum observed threshold, and included gene sets from the Human Disease Ontology (https://disease-ontology.org/)[71] and Human Phenotype Ontology (https://hpo.jax.org/app/)[72] databases. We again used an FDR 5% threshold correction to account for multiple testing to identify gene-sets enriched with *mvAge*.

Mendelian randomization (MR)

Mendelian randomization assumptions.

Mendelian randomization (MR) uses single nucleotide polymorphisms (SNPs) as instrumental variables to find associations between the genetic liability for an exposure trait and an outcome.[73-75] The main assumptions underlying MR are A) SNP instruments associate with the exposure trait of interest ("relevance assumption"); B) SNP instruments only influence the outcome trait through the exposure trait ("exclusion restriction assumption); and C) the frequency of SNP instruments is not influenced by factors that also impact the outcome trait ("exchangeability assumption")[76] (**Extended Data Fig. 5**). In the following sections, we describe the MR methods used in the study and how the methods are implemented to test these assumptions.

Polygenic Mendelian randomization inclusion rationale.

To investigate whether our multivariate GWAS was causally influenced by lifestyle factors and biomarkers, we performed Mendelian randomization (MR) with 73 risk factors and biomarkers derived from GWASs (European ancestry). These risk factors and circulating biomarkers were pre-selected based upon previous associations with healthy aging and longevity, the need to identify causal modifiable risk factors for aging that may inform public health initiatives, interventions, and prevention strategies,[77] and also the lack of reliable causal biomarkers to identify the consequences of aging.[78] We outline below our motivation for selecting these risk factors and biomarkers. We note that we did not assess the relationships of certain disease endpoints with *mvAge*, including cardiovascular disease and Alzheimer's disease, because the diseases are components of two of the input phenotypes, i.e., healthspan and frailty. Healthspan is calculated using that employed Cox-Gompertz survival models with clinical events in seven disease categories, including cancer, cardiovascular disease, diabetes, stroke, and dementia, and its length is defined as the number of years before a participant experiences one or more clinical events in these categories.[79] Similarly, the UK Biobank frailty index (FI) was based upon an accumulation of deficits model[80] using 49 self-reported UK Biobank variables from a range of physical and mental health endpoints, symptoms, disabilities and diagnosed diseases.[81] Therefore, assessing the causal relationships of specific diseases with *mvAge* when healthspan and frailty are themselves functions of various aspects of the diseases would violate the regression independence assumption. A full list and information for each of the of modifiable risk factors and biomarker GWASs is in **Supplementary Table 37**.

Lifestyle risk factors. Enabling older adults to remain independent and maintain sufficient quality of life is an significant ongoing challenge facing aging populations.[82] While lifestyle risk factors such as tobacco smoking, alcohol consumption, sleep, physical activity, and social support have been linked with adverse health outcomes (premature death, chronic disease, etc.),[83,84] studies investigating the long-term impact of these respective lifestyle risk factors is limited, and their causal roles are largely unknown. Further, because the focus of our downstream analyses is the identification of targets for intervention, prevention, and therapeutic strategies for improved aging, we focused our lifestyle risk factor MR analyses on exposures that commonly occur in populations and are also capable of being modified, either by public health initiative, or clinical practice, making them viable targets of various intervention and prevention strategies to improve healthy aging. Therefore, we included exposures related to alcohol consumption, tobacco smoking, sleep, physical activity, and educational attainment. Social interaction, and support networks have also been shown to be related to lifespan and healthy aging,[85-87] and we also assessed markers of isolation, loneliness, and the frequency of attending social activities, i.e., going to a social club, religious group, sports team, or adult education class on *mvAge*.

Biomarkers: circulating lipids, glycemic markers, and blood pressure. Identifying causal biomarkers for healthy aging may provide important information for assessing human aging processes and viable intervention targets for public health and pharmacological strategies;[88] however, biomarkers for aging identified in cross-sectional studies tend to be non-causative.[88] Longitudinal studies to identify causal biomarkers for aging will also be subject cohort selection bias because individuals who age faster (and also have correspondingly higher mortality rates) will be lost over time,[88] making the identification of causal biomarkers for aging particularly challenging.[88] As MR is an important analytic strategy for strengthening causal inference using observational data,[6] we undertook polygenic MR analyses using genetic instruments for a wide range of biomarkers to assess their causal role on *mvAge*.

As lipids have been shown to play important role in regulating aging processes, and a growing body of literature indicates that lipids regulate lifespan in several model organisms.[89] Beyond their roles in energy metabolism and the impact of lipid storage and peroxidation on aging,[89] recent work suggests that lipids are also crucial for several signaling roles in many aging-related processes, including neurodegenerative diseases.[89] In addition, pharmacological modulation of circulating lipid levels has been shown to increase life expectancy among adults aged 50-75 years.[90] Therefore, we included analyses evaluating the impact of several major lipid subfractions, low-density lipoprotein cholesterol (LDL-C), high-density lipoprotein cholesterol (HDL-C), triglycerides, apolipoprotein B, apolipoprotein A1, lipoprotein a Lp(a), and others, on *mvAge*.

Similarly, aging and chronic disease is commonly associated with poor glucoregulatory control and metabolic dysfunction is often observed in aging.[91] Also, many current aging-related therapies result in improved control of glucose homeostasis.[91] However, the causal role of metabolic dysfunction and aging remains to be fully elucidated.[92] Also, given its central role in type 2 diabetes and many other diseases, such as dementia and other neurodegenerative diseases, targeting control of glucose homeostasis may offer a viable target to decrease disease risk and increase healthspan and lifespan.[92] Therefore, we included glycemic markers such as glucose, insulin, and glycated hemoglobin (HbA1c) levels in our biomarker MR analysis.

High blood pressure has been shown reduce life expectancy in middle-aged adults,[93] and blood pressure-lowering via antihypertensive therapy has been shown to reduce frailty and the risk of death among older adults.[94] Similarly, results of the Systolic Blood Pressure Intervention Trial (SPRINT) randomized clinical trial support survival benefits (up to 3 years) of intensive control of blood pressure.[95]

While these biomarkers have established roles of all-cause mortality and survival benefits in patient populations, their associations with life expectancy and healthspan in the general population is less clear. Also, lipids, glycemic markers, and blood pressure represent common downstream biomarkers and physiological responses to pharmacological modulation of commonly prescribed cardiometabolic drug classes that are the subject of our drug-target MR studies assessing the impact on *mvAge* for major drug classes (e.g., PCSK9 inhibition, calcium channel blockers, etc.). Therefore, we also sought to establish the causal role of these biomarkers to establish further rationale for examining the drug targets.

Biomarkers: immune cells and markers of inflammation status. A growing body of literature has begun to document the inverse relationships between immune system functioning and lifespan.[96] It has been suggested that prolonged exposure to infectious agents throughout the life course may result in age-related decreases in immune system functioning – "immunosenescence"[96] and increased inflammation – that may result in increased susceptibility to chronic and infectious disease as individuals age.[96,97] We therefore aimed to clarify the causal roles of immune cell classes (e.g., monocyte, lymphocytes, etc.) and inflammatory markers such as C-reactive protein (CRP), which have been directly implicated in human longevity from observational data.[98]

Biomarkers: markers of liver function. Impaired hepatic function, as marked by alterations in liver function enzyme (LFE) levels in population-based and longitudinal studies, has been previously implicated in future mortality and reduced life expectancy.[15,99,100] Notably, the increased mortality rates extend beyond liver-related causes of death[15,99] (including causes of death such as suicide and injury[100]), suggesting a general role in healthspan and life expectancy that warrants investigation. Therefore, we included several LFEs – alanine aminotransferase (ALT), aspartate aminotransferase (AST), gamma-glutamyltransferase (GGT), and alkaline phosphatase (ALP) – in our biomarker MR analyses.

Biomarkers: organ structure and function. Transcriptomic and proteomic analyses have shown that gene expression and protein in different organs age at different rate, supporting the supposition that organ aging depends on the unique cellular properties and physiological function in the body and the burden of aging on organ structure and function is not equal throughout the body.[101] We therefore included recent GWAS image derived phenotypes constructed from magnetic resonance imaging (MRI) data measuring the organ volume, organ fat content, and organ iron content in approximately 38,000 UK Biobank participants.[102] We included the MRI-derived volumes of the lungs, liver, spleen, and kidneys. We also analyzed the visceral adipose tissue (VAT) and abdominal subcutaneous adipose tissue (ASAT) volumes, liver fat percentage, pancreas fat percentage, liver iron content, and pancreas iron content.[102]

Biomarkers: markers of iron status. Iron is essential for many biochemical processes and systems, including mitochondrial production of ATP, cytochromes, and oxygen transport via hemoglobin. Iron is also known to accumulate with aging and has been shown to be associated with shortened lifespans in many animal models,[103] and a previous longevity GWAS implicated iron metabolism as one of its key findings.[104] Therefore, we aimed to assess the role of biomarkers of iron status, including ferritin, transferrin, liver iron content, and pancreas iron content (discussed in the preceding paragraph).

Biomarkers: miscellaneous. We also extended our biomarker analyses to include several other important biomarkers, such as urinary sodium-potassium ratio, IGF-1, urea, etc., to identify potential causal markers for healthspan and life expectancy.

Polygenic Mendelian randomization methods. For each lifestyle and biomarker exposure, we created genetic instruments using SNPs associated with their respective exposure at conventional genome-wide significance (GWAS) $P < 5\times10^{-8}$ clumped at linkage disequilibrium (LD) $R^2 = 0.001$ (10,000 kb distance), using reference samples comprised of participants of European ancestry.[105] We calculated F-statistics for the instruments for each exposure, which statistics generally exceeded the conventional cutoff of 10, suggesting minimal bias from weak instruments.[106] We extracted SNPs corresponding to the exposure instruments from our multivariate aging GWAS, harmonized exposure and outcome SNPs, and then performed MR analysis: inverse variance weighted (IVW) MR comprises our main method with additional complementary, robust methods developed to estimate consistent causal effects under weaker assumptions than IVW MR (allowing us to assess evidence of causal effects and evaluate the sensitivity of the analyses to different patterns of violations of IV assumptions (MR Egger, weighted median, penalized weighted median, and weighted mode)).[107] We used the Steiger directionality test to evaluate the causal direction,[105] and the Cochran Q heterogeneity test[108] to assess heterogeneity in instrument effects, as heterogeneity may indicate violations of IV assumptions.[109,110,111] If we found evidence of heterogeneity, we used the MR LASSO method, which applies lasso-type penalization to the direct effects of the instruments using a post-lasso estimate, and then performing IVW MR using only those instruments identified as valid instruments (tuning parameter specified at default heterogeneity stopping rule).[111] We used a Bonferroni adjusted threshold P-value = 6.85×10^{-4} (0.05/73

lifestyle and biomarkers exposures). Analyses were carried out using TwoSampleMR, version 0.5.5,[107] and MendelianRandomization, version 0.5.0,[112] in the R environment, version 4.0.2. These MR analyses have been reported in accordance with the STROBE-MR guidelines[113] (**Supplementary Checklist**).

Sample independence.

Our *mvAge* GWAS and many of the biomarker and risk factor exposure GWASs are, at least in part, comprised of data from UK Biobank participants. Sample overlap between exposure and outcome datasets produce may bias IVW estimates in two-sample MR.[114] Further, sample overlap impacts two other sources of potential bias in MR IVW estimates – weak instrument bias and winner's curse (which may happen when the same sample used to select the instruments are used as the exposure dataset).[115] Simulation studies suggest that sample overlap bias is minimal when the MR instruments are strong (i.e., have large F-statistics [F-statistics >10][114]), and when overlapping samples come from large biobanks (i.e., the UK Biobank),[116] suggesting that our results are minimally affected by this source of bias. Nevertheless, we incorporate the MR Lap method, which was recently developed to account for sample overlap (even when the exact overlap percentage is unknown) and also assesses weak instrument bias and winner's curse,[115] as an additional sensitivity test for our polygenic MR analyses. Briefly, MRLap uses cross-trait LD-score regression to evaluate approximate sample overlap and provide a corrected IVW estimate.[115] Per the developer guidelines, we used the MRLap method as a sensitivity analysis and report the MRLap-corrected estimate if it was different than the IVW estimate.

Drug-target MR of antidiabetics, lipid-modulating, and blood pressure-lowering gene targets

See **Extended Data Fig. 6** for a graphical overview of these drug-target MR analyses. First, we obtained summary level data for HbA1c, circulating lipid levels (low-density lipoprotein cholesterol (LDL-C), high density lipoprotein cholesterol (HDL-C), triglycerides (TG), lipoprotein A Lp(a)) and SBP from UK Biobank participants of European ancestry[26,117] (N range: 361,194 to 441,016) that we used to construct our drug target genetic instruments proxying pharmacological modulation of the respective biomarker.

Antidiabetics gene instrument selection.

We performed a look up of antidiabetic medication classes and identified 6 classes of antidiabetic medications with known drug targets (beyond metformin) that may be suitable for genetic instrumentation.[118] These antidiabetic classes are: glucagon-like peptide-1 receptor (GLP-1R) analogs, insulin analogs, sulfonylureas, dipeptidyl peptidase 4 (DPP-IV) inhibitors, and sodium-glucose cotransporter-2 (SGLT2) inhibitors. Next, we used the DrugBank and ChEMBL databases[119,120] to identify the pharmacologically active targets for sulfonylureas (*ABCC8* and *KCNJ11*), insulin analogs (*INSR*), and thiazolidinediones (TZD) (*PPARG*). For every target, we evaluated whether there were suitable genetic variants located within the cis-acting loci of the target gene boundaries (100 kb on either side of gene boundaries) within the glycated hemoglobin (HbA1c) GWAS data derived from participants in the UK Biobank.[117] For our genetic instruments proxying GLP-1R analogs, sulfonylureas, SGLT2 inhibitors, and thiazolidinediones were extracted variants at conventional genome-wide statistical significance (P-value < 5×10^{-8}) that were within the cis-acting loci of the target gene boundaries. For DPP-IV inhibitors and INSR analogs, we used a relaxed P-value threshold (5×10^{-4}) because there were no variants at conventional genome-wide statistical significance in the in either the *INSR* or the *DPP4* locus. See **Supplementary Table 40** for GWAS association statistics for each of the SNPs comprising the antidiabetic target instruments.

Lipid-modulating gene targets. In addition to analyzing the genetic targets of approved lipid-modulating therapies, we performed a look up of potential/proposed lipid-modulating targets.[121] We proxied pharmacological modulation of these lipid drug targets by extracting SNPs that were associated with their respective biomarker that is the primary physiological response to pharmacological modulation of that target (LDL-C lowering therapies, TG lowering therapies, and HDL-C raising therapies). For every target we evaluated whether there were suitable genetic variants located within the cis-acting loci of the target gene boundaries (100 kb on either side of gene boundaries) for their respective lipid. Note that our initial look up of lipid-modulating therapies included the *DGAT1* and *DGAT2* genes (important in TG metabolism);[122] however, there were no variants within these genomic loci with suitable association statistics with the TG GWAS data. Therefore, *DGAT1* and *DGAT2* were excluded from further analysis. For 14 of the remaining 15 lipid-modulating drug targets, we extracted variants at conventional genome-wide statistical significance (P-value < 5×10^{-8}) that were within the cis-acting loci of the target gene boundaries ($\pm$100 kb of gene boundaries). We used a relaxed P-value threshold (P-value < 5×10^{-4}) for instrumenting *ACLY* in LDL-C data because there were no variants at conventional genome-wide statistical significance in the *ACLY* locus. See **Supplementary Table 41** for SNP association information for each of the lipid-modulation target instruments.

We aimed to replicate lipid-modulating results surpassing our drug target MR P-value threshold adjusted for multiple comparisons (P-value = 1.92×10^{-3} [0.05/26 total drug targets tested across antidiabetics, lipid-modulating therapies, and antihypertensives]) using additional instruments derived from independent GWAS data from the 2013 Global Lipids Genetics consortium (GLGC) meta-analyses of participants of European ancestry (N ≤ 172,082).[123] We were able to construct genetic instruments to perform replication analyses for the PCSK9, ABCG5/8, CETP, and LPL findings (there were no suitable variants within the *ANGPTL4* locus).

Antihypertensive drug targets. As with the above antidiabetic and lipid-modulating instrument selection processes, we first performed a look up of antihypertensive drug classes,[124] and we used the DrugBank and ChEMBL databases[119,120] to identify genes encoding the antihypertensive drug targets for 5 classes of antihypertensives: angiotensin converting enzyme (ACE) inhibitors, angiotensinogen (AGT) inhibitor, beta blockers, calcium channel blockers (CCBs), and thiazide diuretics (TDs).[124] We selected SNPs again within 100 kb of the gene boundaries as proxies for lowering systolic blood pressure (SBP) via these gene targets. Note that for our genetic instrument for CCBs, in addition to analyzing each gene target separately, we also concatenated the CCB instrument by combining several of the individual gene targets (i.e., *CACNA1D, CACNA2D2, CACNB2, CACNB3)* into a single CCB instrument, as has been done previously,[125] and is similar to the metformin instrumentation procedure (**Supplementary Table 5**). Also note that we used a relaxed P-value threshold (< 5×10^{-6}) because there were no SNPs with P-value < 5×10^{-8} in or near the loci encoding the targets for AGT inhibitors (*AGT*) there were no conventionally genome-wide significant variants associated with SBP at this locus. In addition, there were no sufficient variants within the *SLC12A3* locus, the target for TDs, and therefore, we did not analyze this target. See **Supplementary Table 42** for GWAS summary association information for each of the SNPs comprising the antihypertensive target instruments.

Drug-target MR statistical methods. First, we clumped extracted SNPs at LD R^2 < 0.2 threshold (250 kb window) using the 1000 Genomes Project EUR reference population,[126] and calculated F-statistics to evaluate instrument strength to assess the first MR assumption. We used the cutoff of F-statistic > 10 to as suggesting the resulting estimates would be subject to minimal bias from weak instruments, which is especially important for the drug-target instruments comprised of variants with relaxed P-value thresholds due to absent genome-wide significant variants at those loci in the respective biomarker GWAS data (i.e., DPP-IV inhibitors, ACLY, and several genes in the instrument for calcium channel blockers).[106] See **Supplementary Tables 40-42** for F-statistic and R^2 calculations for each of the instruments SNPs. After harmonization with *mvAge*, we performed correlated MR IVW (random-effects analysis performed when there were more than three variants) and MR Egger analyses accounting for the correlation between our instrument variants (the requisite correlation matrices for the analyses were generated using the 1000 Genomes Phase 3 EUR population as reference[126]). We used correlated MR IVW as the primary method so as to increase precision by including additional, partially independent variants in the drug-target instruments.[7,127] We also performed heterogeneity testing; where heterogeneity tests suggested evidence of heterogeneity with the MR estimates (i.e., $Q_{P\text{-value}} < 0.05$), we repeated the analyses with a more stringent LD R^2 threshold of 0.1 to remove potential heterogenous variants.

To facilitate interpretation of these classes of drugs, we oriented the MR effect estimates to reflect the direction of the expected physiological responses to the pharmacological modulation of the drug targets. For the antidiabetics, we oriented MR estimates to correspond to the glucose/HbA1c-lowering mechanisms of DPP-IV, GLP-1, and metformin, and we scaled MR estimates to correspond to a lowering effect in the HbA1c GWAS data (note that GLP-1 agonists lower HbA1c). For the LDL-C and TG lowering drug-targets, we oriented MR estimates to correspond to lower circulating LDL-C and TG levels (note that for LPL this is achieved by enhancing LPL activity). We oriented APOA1, and CETP inhibition estimates to correspond to an increase in circulating HDL-C levels. Finally, we oriented the antihypertensive estimates to correspond to a lowering in SBP. We used a Bonferroni adjusted P-value threshold = 0.002 (0.05/25 total drug targets tested across antidiabetics, lipid-modulating therapies, and antihypertensives) as a heuristic to guide follow-up analyses on a plausible number of findings.

Sample overlap.

For the drug-target MR analyses we used MR methods incorporating LD information by constructing SNP-SNP correlation matrices as part of the instrument.[112] MRLap cannot be applied to correlated MR methods; thus, we did not use MRLap with the drug-target MR. However, as discussed above, simulation studies suggest that sample overlap bias is minimal when the MR instruments are strong (i.e., F-statistics > 10[114]), and when overlapping samples come from large biobanks (i.e., the UK Biobank),[116] suggesting that our drug-target (and cis-instrument MR scan of protein coding genes in these biomarkers) results are minimally affected by this source of bias.

Cis-instrument Mendelian randomization of protein-coding genes in biomarker data background

In addition to recent proposals to integrate biomarker data for drug discovery for cardiometabolic diseases,[7,128] it has been suggested that integrating biomarker data will help identify the causal pathways linking variants with healthspan and life expectancy.[129] Therefore, after identifying strong genetic evidence in our polygenic MR analyses of biomarkers that lipids, glycemic markers, and blood pressure are important biomarkers for *mvAge*, and also in our drug-target MR analyses that their modulation via targets of both proposed and candidate therapies have beneficial effects on *mvAge,* we undertook a cis-instrument MR scan of protein coding genes associated with these biomarkers, with the aim of identifying potential therapeutic targets that will inform future investigation. **Extended Data Figs. 6-7** presents a graphical overview of these analyses.

Selection of drug target genes and statistical analysis.

We used the same lipids, glycated hemoglobin, and SBP GWAS data as used for the drug target MR analyses described above. First, we extracted lead variants with P-values < 5×10^{-8} (LD $R^2 < 0.1$) associated with their respective GWAS biomarker. Next, we used the biomaRt package,[130] to identify and curate protein-coding genes located within 50 kb of these lead variants (**Supplementary Tables 43-47**). Because the validity of genetic instruments in MR relies on three assumptions, we used a systematic selection approach to first prioritize protein-coding genes with valid MR instruments. After curating the list of protein coding genes located near the lead variants in each of the biomarker GWAS summary statistics, we performed colocalization analyses to assess the posterior probability of a shared genetic signal between *mvAge* and biomarkers at the protein-coding gene locus.[65] We identified 6,718 protein coding genes located within 50 kb of the lead SNPs in the biomarker GWAS. There was overlap in the protein coding genes identified across the biomarkers, and in total, we included 3,947 unique protein coding genes in the analyses. We used the *coloc* R package,[65] and included all cis variants within 50 kb of the gene boundaries and we performed colocalization analyses for 994 (HbA1c), 1,787 (HDL-C), 873 (LDL-C), 2,216 (TG), and 848 (SBP) protein coding genes (**Supplementary Tables 18-21**). We considered genes with posterior probabilities (PP.H4) > 0.6 as suggestive evidence of a shared causal variant between *mvAge* and the respective biomarker within the gene locus and we took these protein-coding genes forward to downstream drug-target MR analyses (**Supplementary Tables 22-25**). Across the 5 biomarkers, 523 genes surpassed the colocalization posterior probability (PP.H4) > 0.6: 84 for HbA1c, 89 for HDL-C, 128 for LDL-C, 125 for TG, and 97 for SBP. We took these genes forward for cis-instrumentation in preparation for MR analysis. We constructed cis-instruments for the drug targets using genetic variants (LD $R^2 <$ 0.2) associated with their respective biomarkers at conventional genome-wide statistical significance (P-value <

5×10^{-8}) located within the boundaries of the gene. We calculated F-statistics[106] for each cis-instrument proxying the protein-coding gene to assess the first MR assumption that the instrument is strongly associated with the exposure (here the protein-coding gene) and only included variants with F-statistics surpassing the conventional threshold of 10 that suggests minimal bias from weak instruments.[106] 354 of the 523 genes had conditionally independent variants within the gene loci meeting these criteria; we took these 354 genes forward to harmonization and cis-instrument MR with *mvAge*. As with the previous drug-target MR analyses, for cis-instruments with ≥2 variants, we used MR IVW (random-effects analysis performed when there were more than three variants), MR Maximum Likelihood, and MR Egger analyses accounting for the correlation between the variants. For cis-instruments comprised of a single variant, we used the Wald ratio method. We aligned effect estimates with the established direction of therapeutic targets for the respective biomarker (e.g., Hba1c lowering, LDL-C lowering, HDL-C raising, and SBP lowering). We accounted for multiple comparisons with a Bonferroni-corrected threshold P-value = 1.41×10^{-4} (0.05/354 genes tested) as a heuristic to follow-up on a plausible number of findings. We aimed to replicate genes surpassing correction for multiple comparisons using independent biomarker data. For the HDL-C, LDL-C, and TG, we used the 2013 Global Lipids Genetics consortium (GLGC) meta-analyses of participants of European ancestry (N ≤ 172,082),[123] and for HbA1c, we used the recent meta-analysis of individuals of European ancestry without type 2 diabetes performed by the MAGIC consortia (N=200,662).[131] We were unable to obtain an independent GWAS for SBP and, therefore, did not perform replication of the SBP findings. Further, we were unable to identify any cis variants associated with HbA1c at conventional genome-wide significance within the gene boundaries. Therefore, we extended the genomic coordinate window to include variants within 50 kb of the gene boundaries, which is still a stringent threshold for cis-instrument MR (e.g., drug-target MR studies commonly define cis loci as any variants within approximately ±100 kb of the gene locus[7,128,132]). We were able to cis-instrument 41 genes across the 4 biomarkers (out of 132 genes for HbA1c, HDL-C, LDL-C, and triglycerides surpassing Bonferroni corrected threshold P-value = 1.41×10^{-4} (0.05/354 genes tested) for replication using the same instrument selection protocol as outlined above.

Intersection of genes with drug targets.

We aimed to evaluate the potential therapeutic actionability of our top protein-coding genes. First, we used a downloaded the list of druggable genes defined by Finan et al., who created a three tier system assessing potential druggability for genes:[133] Tier 1 genes include targets of both approved small compounds and clinical-phase drug candidates; Tier 2 genes include genes with annotated bioactivity for drug-like small molecules, and Tiers 3A-3B include genes encoding secreted or extracellular proteins, and genes are members of important druggable gene families, such as G-protein coupled receptors (GCPRs), nuclear hormone receptors, ion channels, kinases, and phosphodiesterases.[133] **Supplementary Table 48** lists the 4,676 compiled druggable genes (the original 4,479 genes identified by Finan et al.[133] supplemented by 192 additional druggable genes identified by Gaziano et al.[134]). We next queried additional protein interaction databases to further annotate the protein coding genes and identify possible actionable therapeutics. First, we downloaded the STITCH interaction database (protein/chemical links in humans)[135] and assessed each protein/chemical connections. In our analyses, we used the STITCH guidelines and considered connections with a "combined score" of ≥ 0.90 as high confidence connections.[135] We also downloaded the latest interactions data from DGIdb[136] ("interactions.tsv", "genes.tsv", "drugs.tsv" from February 2022) and tested whether the cis-instrument MR genes were targets for therapeutics.

Cis-instrument Mendelian randomization proxying circulating proteins

In addition to performing an cis-instrument MR scan of protein coding genes using lipids, glycemic markers, and blood pressure GWAS data, we also explored the causal role of circulating proteins measured in 30,391 participants of European ancestry from the first results provided by the SCALLOP consortium,[137] which used the Olink platform to perform a protein quantitative trait loci (pQTLs) mapping of plasma proteins (https://www.olink.com/scallop/) to investigate potential anti-aging therapeutic opportunities that may be useful for future investigation. We included pQTLs that were associated with the plasma protein at P-value < 5×10^{-8} and within the cis-acting loci of the target gene boundaries (100 kb either side of gene boundaries), which left 68 pQTLs for the exploratory scan with our multivariate GWAS (**Supplementary Table 49**). We calculated F-statistics for the instruments for each exposure, which statistics generally exceeded the conventional cutoff of 10, suggesting minimal bias from weak instruments,[106] and as above, we clumped extracted SNPs at the LD $R^2 \leq 0.2$ threshold (250 kb distance) using the

1000 Genomes Phase 3 European reference population,[126] and performed correlated MR IVW (random-effects analysis performed when there were more than three variants, fixed-effects otherwise) and MR Egger accounting for the correlation between the instrument variants. We used a Bonferroni adjusted threshold P-value = 7.35×10^{-4} (0.05/68 proteins compared). We aimed to replicate the findings of the proteins surpassing the adjusted threshold using additional pQTL-based analysis of circulating proteins.[138,139] Of the 8 proteins associated with *mvAge* identified in the SCALLOP data, we were able to cis-instrument 6 of the top proteins: Colony Stimulating Factor 1 (CSF-1), Cystatin B (CSB), C-X3-C Motif Chemokine Ligand 1 (CX3CL1), Interleukin-6 Receptor Subunit Alpha (IL-6RA), Matrix Metallopeptidase 1 (MMP-1), and Matrix Metallopeptidase 10 (MMP-10) (**Supplementary Table 50**). Clumping, harmonization, and MR analysis proceeded as above. However, the instrument for CX3CL1 contained only one SNP, and therefore, we used the Wald Ratio method[107] to obtain the effect estimate. All drug-target MR analyses were performed using the MendelianRandomization R package,[112] and R version 4.0.2. These drug-target MR analyses have been reported in accordance with the STROBE-MR guidelines[113] (**Supplementary Checklist**).

SUPPLEMENTARY CHECKLIST: STROBE-MR Reporting Guidelines.

1. TITLE and ABSTRACT
Indicate Mendelian randomization as the study's design in the title and/or the abstract.
Not applicable for title (GWAS study). MR discussed in abstract.

INTRODUCTION
2. Background
Explain the scientific background and rationale for the reported study. Is causality between exposure and outcome plausible? Justify why MR is a helpful method to address the study question.
Addressed in the Introduction and Methods and Supplementary Methods

3. Objectives
State specific objectives clearly, including pre-specified causal hypotheses (if any).
Addressed in the Introduction and Methods.

METHODS
4. Study design and data sources
Present key elements of study design early in the paper. Consider including a table listing sources of data for all phases of the study. For each data source contributing to the analysis, describe the following:
a) Describe the study design and the underlying population from which it was drawn. Describe also the setting, locations, and relevant dates, including periods of recruitment, exposure, follow-up, and data collection, if available.
b) Give the eligibility criteria, and the sources and methods of selection of participants.
c) Explain how the analyzed sample size was arrived at.
d) Describe measurement, quality and selection of genetic variants.
e) For each exposure, outcome and other relevant variables, describe methods of assessment and, in the case of diseases, the diagnostic criteria used.
f) Provide details of ethics committee approval and participant informed consent, if relevant.
Addressed in the Methods and Supplementary Methods.

5. Assumptions
Explicitly state assumptions for the main analysis (e.g. relevance, exclusion, independence, homogeneity) as well assumptions for any additional or sensitivity analysis.
Addressed in the Supplementary Methods.

6. Statistical methods: main analysis
Describe statistical methods and statistics used.
a) Describe how quantitative variables were handled in the analyses (i.e., scale, units, model).
b) Describe the process for identifying genetic variants and weights to be included in the analyses (i.e, independence and model). Consider a flow diagram.
c) Describe the MR estimator, e.g. two-stage least squares, Wald ratio, and related statistics. Detail the included covariates and, in case of two-sample MR, whether the same covariate set was used for adjustment in the two samples.
d) Explain how missing data were addressed.
e) If applicable, say how multiple testing was dealt with.
Addressed in the Methods and Supplementary Methods.

7. Assessment of assumptions
Describe any methods used to assess the assumptions or justify their validity.
Addressed in the Methods and Supplementary Methods.

8. Sensitivity analyses
Describe any sensitivity analyses or additional analyses performed.
Addressed in the Methods and Supplementary Methods.

9. Software and pre-registration
a) Name statistical software and package(s), including version and settings used.
Addressed in the Methods.

b) State whether the study protocol and details were pre-registered (as well as when and where).
Addressed in the Methods.

RESULTS
10. Descriptive data
a) Report the numbers of individuals at each stage of included studies and reasons for exclusion. Consider use of a flow-diagram.
b) Report summary statistics for phenotypic exposure(s), outcome(s) and other relevant variables (e.g. means, standard deviations, proportions).
c) If the data sources include meta-analyses of previous studies, provide the number of studies, their reported ancestry, if available, and assessments of heterogeneity across these studies. Consider using a supplementary table for each data source.
d) For two-sample Mendelian randomization:
i. Provide information on the similarity of the genetic variant-exposure associations between the exposure and outcome samples.

ii. Provide information on extent of sample overlap between the exposure and outcome data sources.
Addressed in the Methods, Results, Supplementary Results, Supplementary Tables.

11. Main results
a) Report the associations between genetic variant and exposure, and between genetic variant and outcome, preferably on an interpretable scale (e.g. comparing 25th and 75th percentile of allele count or genetic risk score, if individual-level data available).
b) Report causal effect estimate between exposure and outcome, and the measures of uncertainty from the MR analysis. Use an intuitive scale, such as odds ratio, or relative risk, per standard deviation difference.
c) If relevant, consider translating estimates of relative risk into absolute risk for a meaningful time-period.
d) Consider any plots to visualize results (e.g. forest plot, scatterplot of associations between genetic variants and outcome versus between genetic variants and exposure).
Addressed in the Results, Supplementary Results, and Supplementary Tables.

12. Assessment of assumptions
a) Assess the validity of the assumptions.
b) Report any additional statistics (e.g., assessments of heterogeneity, such as I2, Q statistic).
Addressed in the Results, Supplementary Tables, Discussion and Supplementary Discussion.

13. Sensitivity and additional analyses
a) *Use sensitivity analyses to assess the robustness of the main results to violations of the assumptions.*
b) Report results from other sensitivity analyses (e.g., replication study with different dataset, analyses of subgroups, validation of instrument(s), simulations, etc.).
c) Report any assessment of direction of causality (e.g., bidirectional MR).
d) When relevant, report and compare with estimates from non-MR analyses.
e) Consider any additional plots to visualize results (e.g., leave-one-out analyses).
Addressed in the Results, Supplementary Results, and Supplementary Tables.

DISCUSSION
14. Key results
Summarize key results with reference to study objectives.
Addressed in the Discussion and Supplementary Discussion.

15. Limitations
Discuss limitations of the study, taking into account the validity of the MR assumptions, other sources of potential bias, and imprecision. Discuss both direction and magnitude of any potential bias, and any efforts to address them.
Addressed in the Discussion and Supplementary Discussion.

16. Interpretation
a) Give a cautious overall interpretation of results considering objectives and limitations. Compare with results from other relevant studies.

b) Discuss underlying biological mechanisms that could be modelled by using the genetic variants to assess the relationship between the exposure and the outcome.
c) Discuss whether the results have clinical or policy relevance, and whether interventions could have the same size effect.
Addressed in the Discussion and Supplementary Discussion.

17. Generalizability
Discuss the generalizability of the study results (a) to other populations (i.e. external validity), (b) across other exposure periods/timings, and (c) across other levels of exposure.
Addressed in the Discussion and Supplementary Discussion.

OTHER INFORMATION
18. Funding
Give the source of funding and the role of the funders for the present study and, if applicable, for the original study or studies on which the present article is based.
Addressed in the Funding.

19. Data and data sharing
Present data used to perform all analyses or report where and how the data can be accessed. State whether statistical code is publicly accessible and if so, where.
Addressed in the Methods.

20. Conflicts of Interest
All authors should declare all potential conflicts of interest.
Addressed in the Conflicts of interest.

SUPPLEMENTARY FIGURES

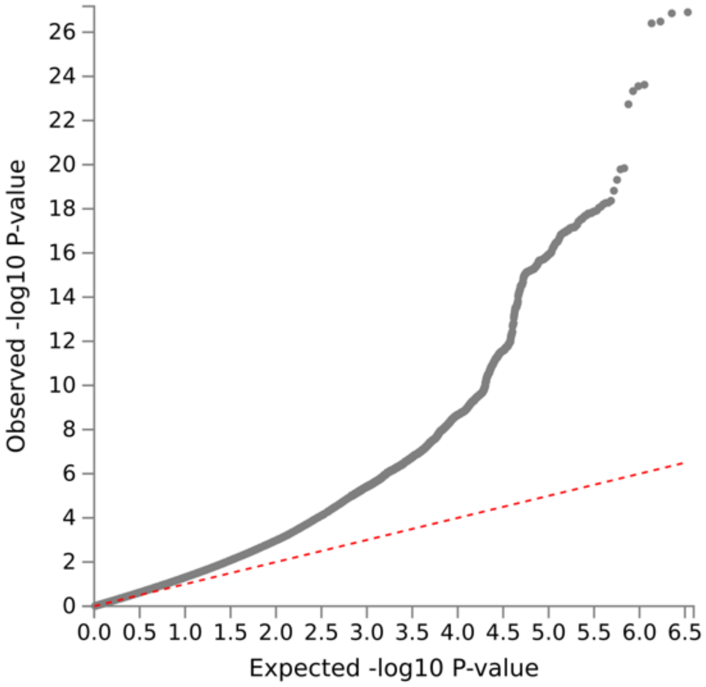

Supplementary Fig. 1. Multivariate GWAS Quantile-Quantile (Q-Q) plot.
Plot represents a comparison of the *mvAge* multivariate genome-wide association study (GWAS) (N=1,958,774) P-values to those expected for null distribution (depicted by the red line). See the **Results** discussion for additional information.

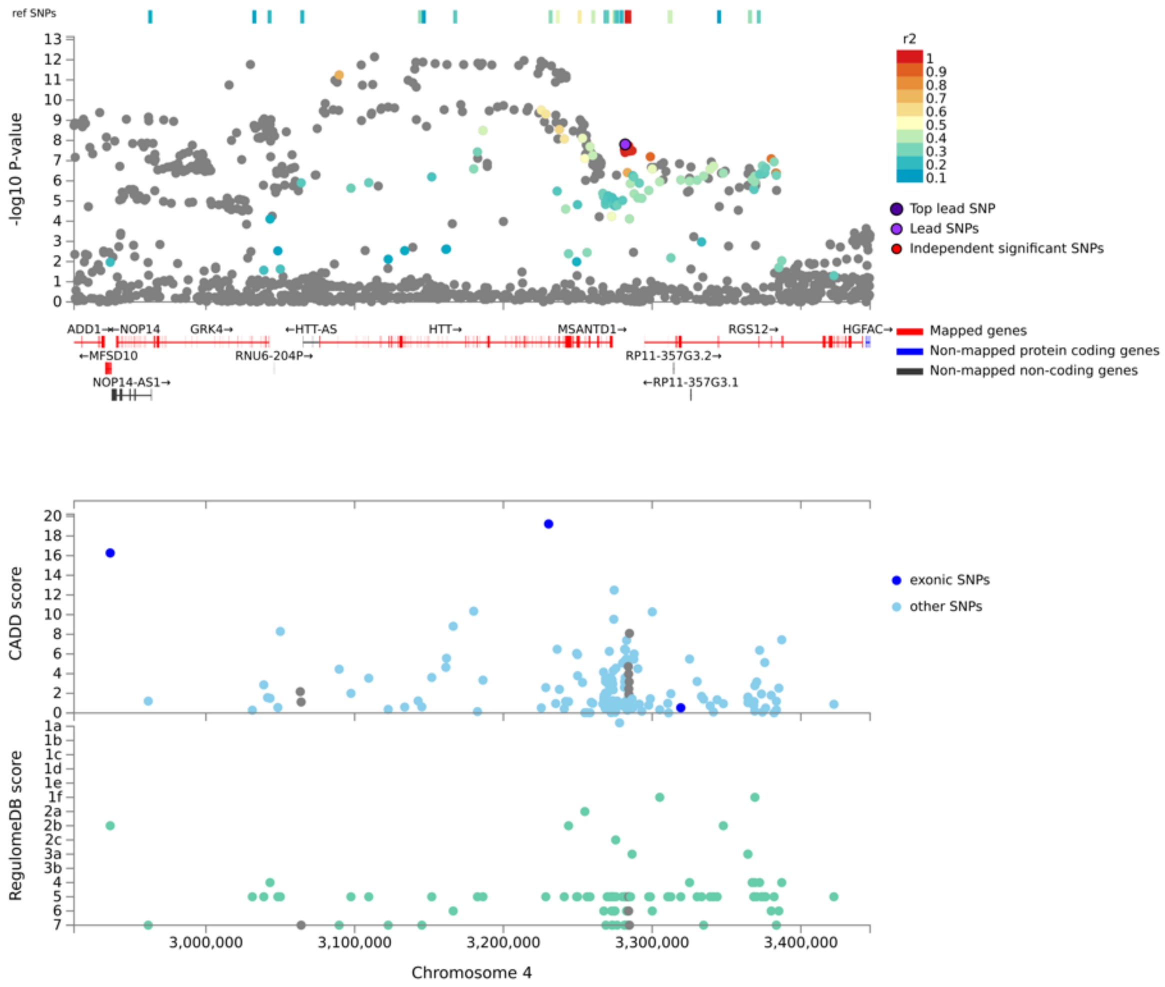

Supplementary Fig. 2. Regional association plot for SNP rs114298671.
Regional plot of the locus around the lead variant rs114298671 identified in the *mvAge* multivariate genome-wide association study (GWAS) (N=1,958,774). **Top** Top panel presents the -log10(P-values) results of two-sided Wald tests for each variant on *mvAge* and linkage disequilibrium (LD) R^2 information for the variants in the locus (variants are colored by LD R^2) and the genes prioritized by FUMA are highlighted in red on the track below. **Bottom** The bottom panel presents the CADD (combined annotation dependent depletion) scores and RegulomeDB scores (top and bottom tracks, respectively).

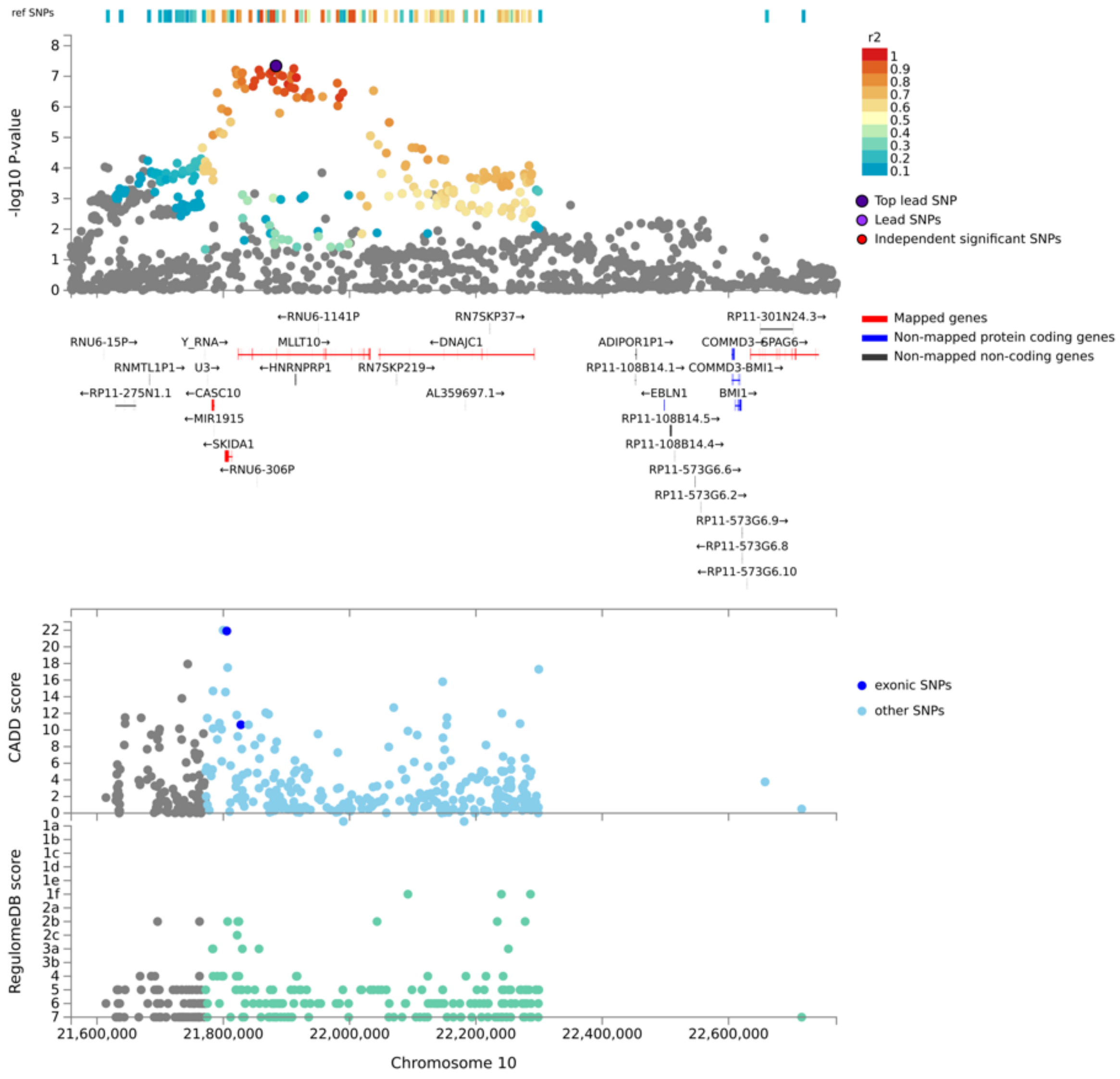

Supplementary Fig. 3. Regional association plot for SNP rs12769128.
Regional plot of the locus around the lead variant **rs12769128** identified in the *mvAge* multivariate genome-wide association study (GWAS) (N=1,958,774). **Top** The top panel presents the -log10(P-values) results of two-sided Wald tests for each variant on *mvAge* and linkage disequilibrium (LD) R^2 information for the variants in the locus (variants are colored by LD R^2) and the genes prioritized by FUMA are highlighted in red on the track below. **Bottom** The bottom panel presents the CADD (combined annotation dependent depletion) scores and RegulomeDB scores (top and bottom tracks, respectively).

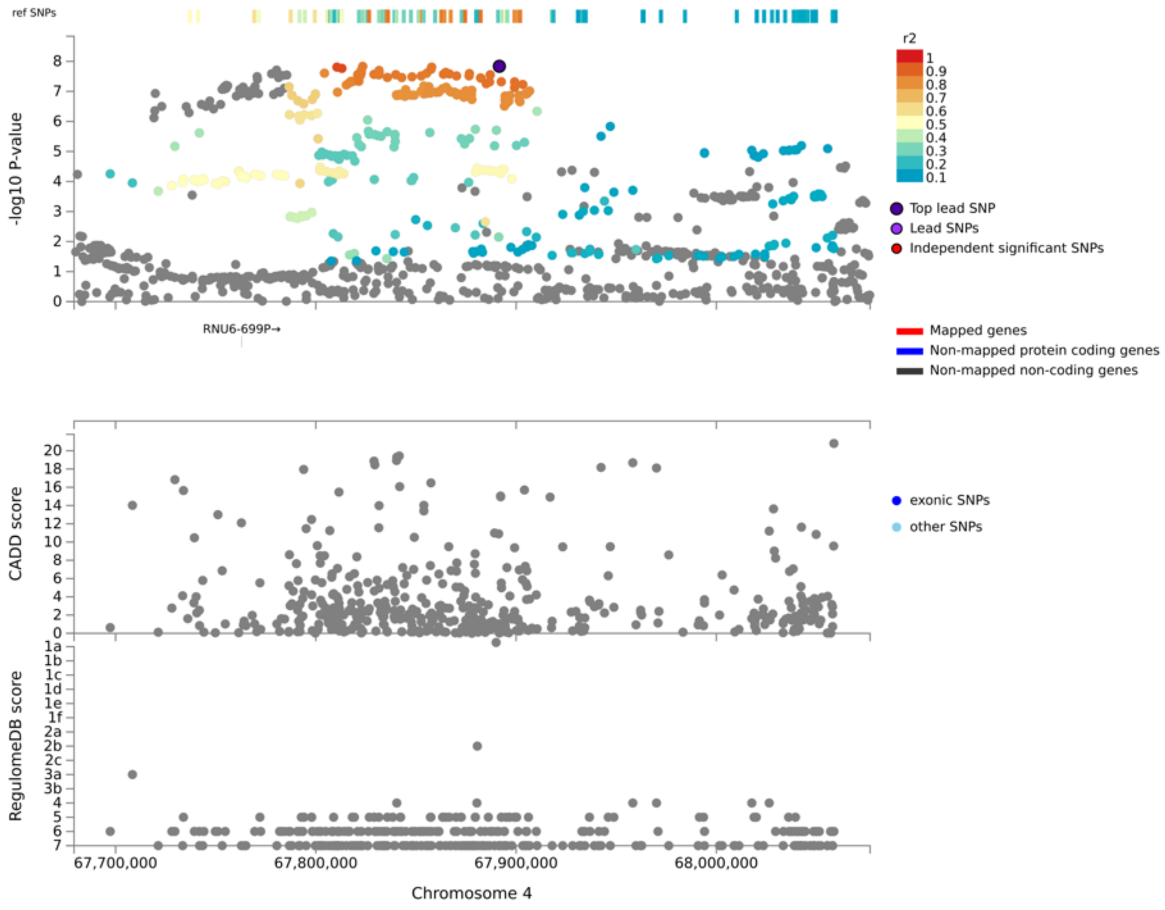

Supplementary Fig. 4. Regional association plot for SNP rs13141210.
Regional plot of the locus around the lead variant rs13141210 identified in the *mvAge* multivariate genome-wide association study (GWAS) (N=1,958,774). **Top** The top panel presents the -log10(P-values) results of two-sided Wald tests for each variant on *mvAge* and linkage disequilibrium (LD) R^2 information for the variants in the locus (variants are colored by LD R^2) and the genes prioritized by FUMA are highlighted in red on the track below. **Bottom** The bottom panel presents the CADD (combined annotation dependent depletion) scores and RegulomeDB scores (top and bottom tracks, respectively).

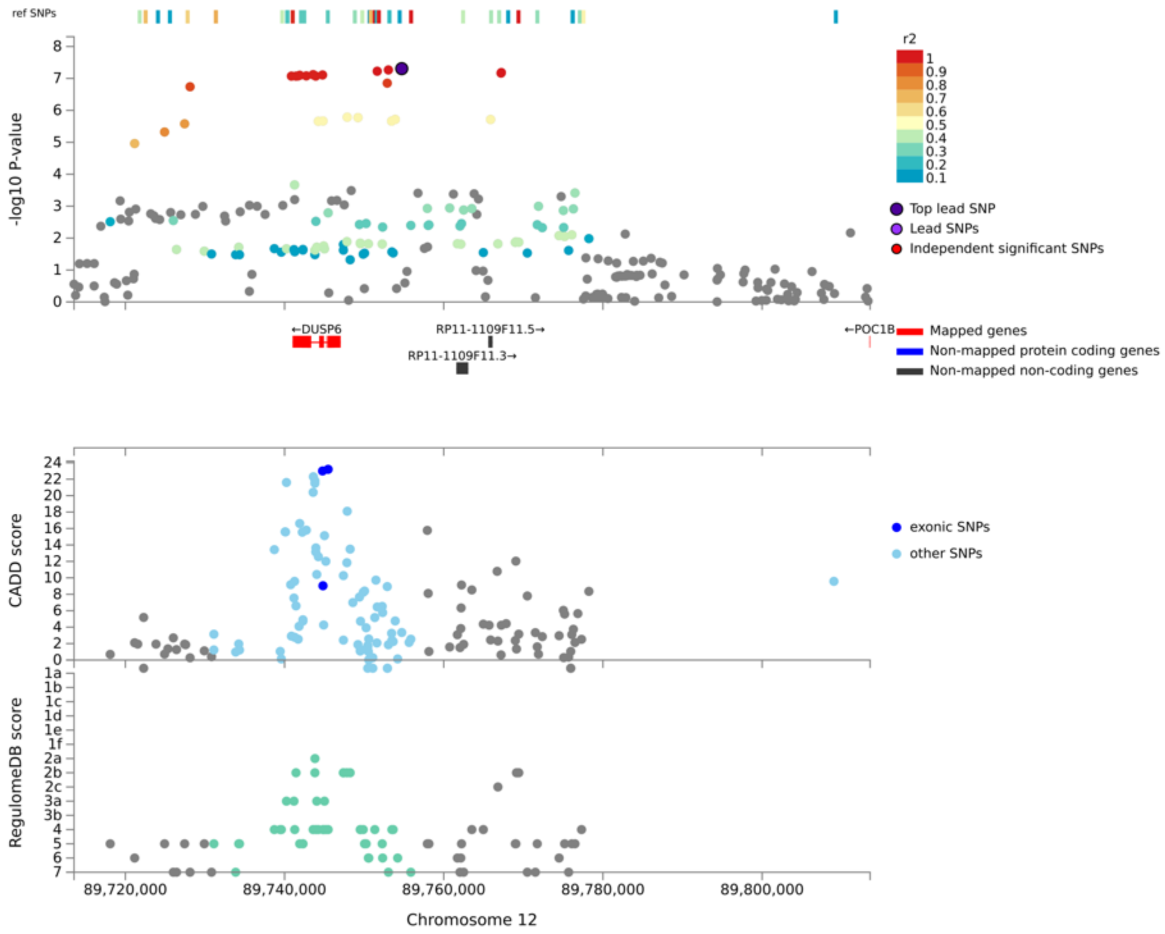

Supplementary Fig. 5. Regional association plot for SNP rs1689406.
Regional plot of the locus around the lead variant rs1689406 identified in the *mvAge* multivariate genome-wide association study (GWAS) (N=1,958,774). **Top** The top panel presents the -log10(P-values) results of two-sided Wald tests for each variant on *mvAge* and linkage disequilibrium (LD) R^2 information for the variants in the locus (variants are colored by LD R^2) and the genes prioritized by FUMA are highlighted in red on the track below. **Bottom** The bottom panel presents the CADD (combined annotation dependent depletion) scores and RegulomeDB scores (top and bottom tracks, respectively).

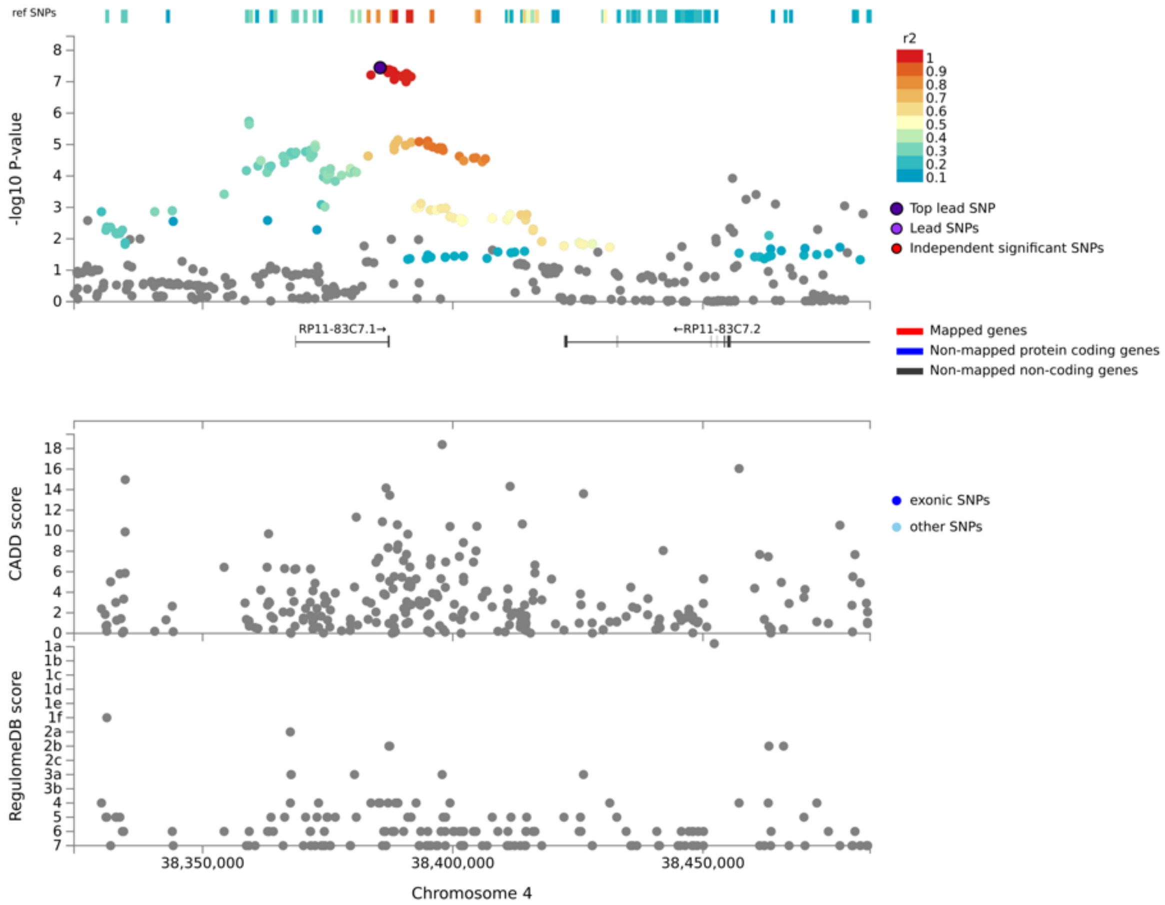

Supplementary Fig. 6. Regional association plot for SNP rs17499404.
Regional plot of the locus around the lead variant rs17499404 identified in the *mvAge* multivariate genome-wide association study (GWAS) (N=1,958,774). **Top** Top panel presents the -log10(P-values) results of two-sided Wald tests for each variant on *mvAge* and linkage disequilibrium (LD) R^2 information for the variants in the locus (variants are colored by LD R^2) and the genes prioritized by FUMA are highlighted in red on the track below. **Bottom** The bottom panel presents the CADD (combined annotation dependent depletion) scores and RegulomeDB scores (top and bottom tracks, respectively).

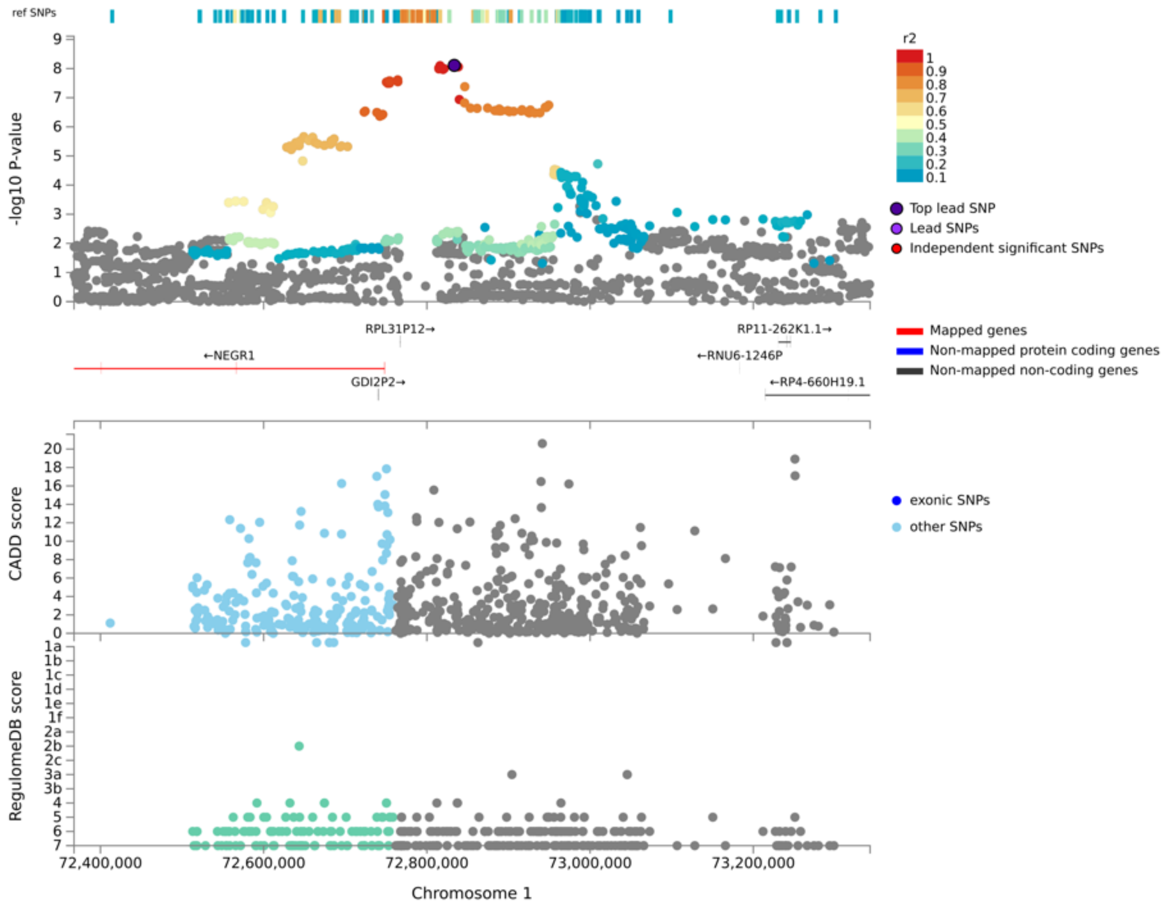

Supplementary Fig. 7. Regional association plot for SNP rs2613508.
Regional plot of the locus around the lead variant rs2613508 identified in the *mvAge* multivariate genome-wide association study (GWAS) (N=1,958,774). **Top** Top panel presents the -log$_{10}$(P-values) results of two-sided Wald tests for each variant on *mvAge* and linkage disequilibrium (LD) R^2 information for the variants in the locus (variants are colored by LD R^2) and the genes prioritized by FUMA are highlighted in red on the track below. **Bottom** The bottom panel presents the CADD (combined annotation dependent depletion) scores and RegulomeDB scores (top and bottom tracks, respectively).

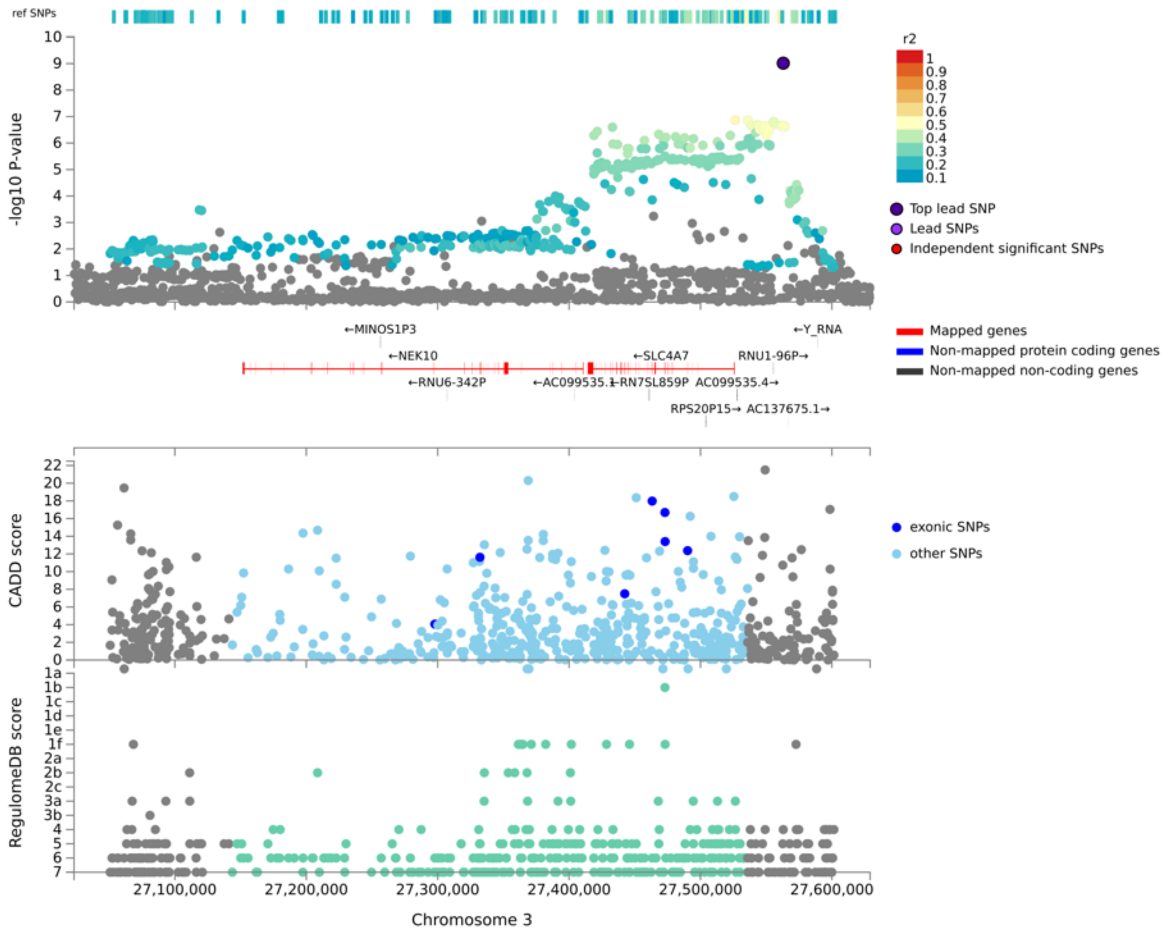

Supplementary Fig. 8. Regional association plot for SNP rs2643826.
Regional plot of the locus around the lead variant rs2643826 identified in the *mvAge* multivariate genome-wide association study (GWAS) (N=1,958,774). **Top** The top panel presents the -$\log_{10}$(P-values) results of two-sided Wald tests for each variant on *mvAge* and linkage disequilibrium (LD) R^2 information for the variants in the locus (variants are colored by LD R^2) and the genes prioritized by FUMA are highlighted in red on the track below. **Bottom** The bottom panel presents the CADD (combined annotation dependent depletion) scores and RegulomeDB scores (top and bottom tracks, respectively).

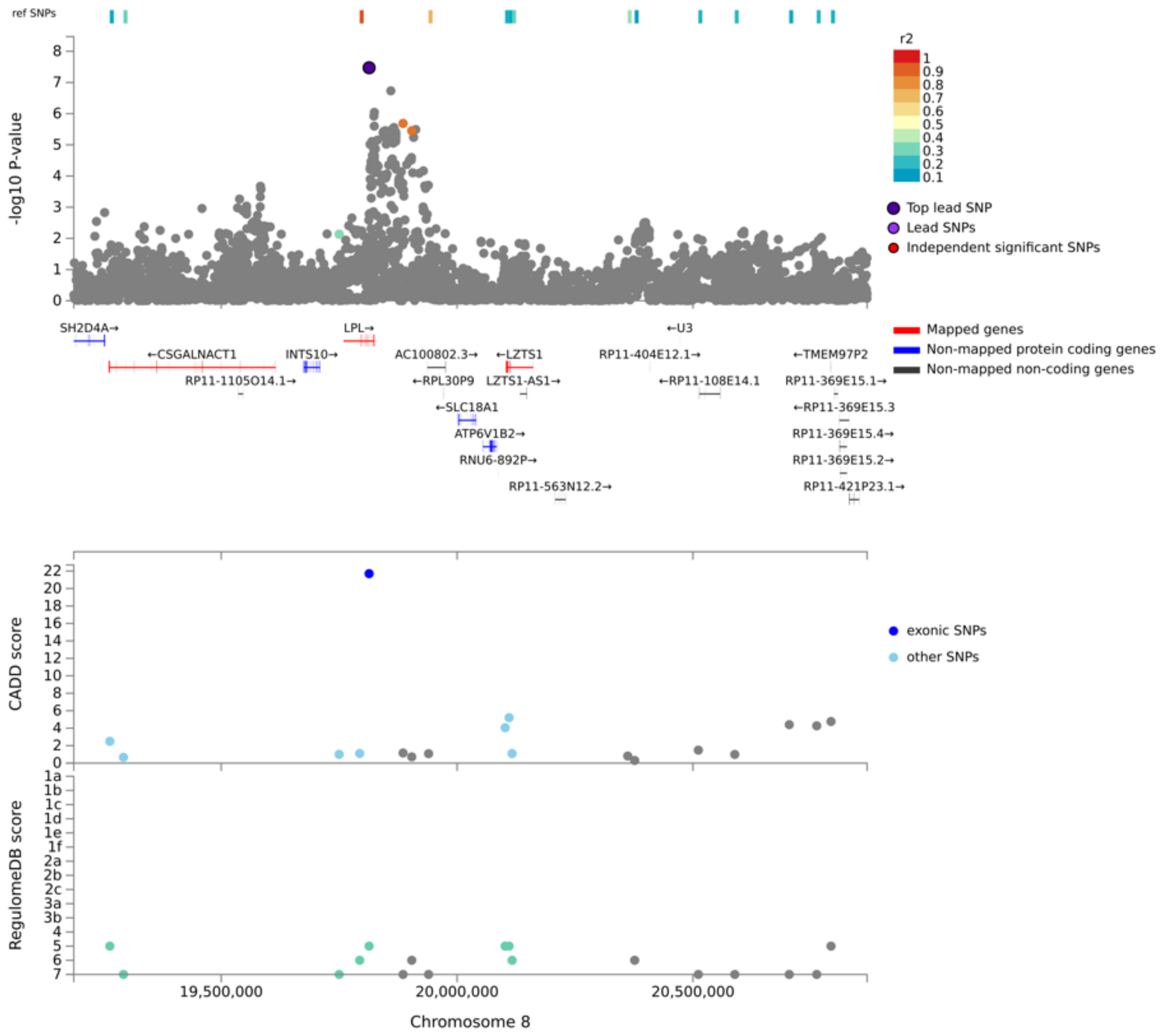

Supplementary Fig. 9. Regional association plot for SNP rs268.
Regional plot of the locus around the lead variant rs268 identified in the *mvAge* multivariate genome-wide association study (GWAS) (N=1,958,774). **Top** The top panel presents the -log10(P-values) results of two-sided Wald tests for each variant on *mvAge* and linkage disequilibrium (LD) R^2 information for the variants in the locus (variants are colored by LD R^2) and the genes prioritized by FUMA are highlighted in red on the track below. **Bottom** The bottom panel presents the CADD (combined annotation dependent depletion) scores and RegulomeDB scores (top and bottom tracks, respectively).

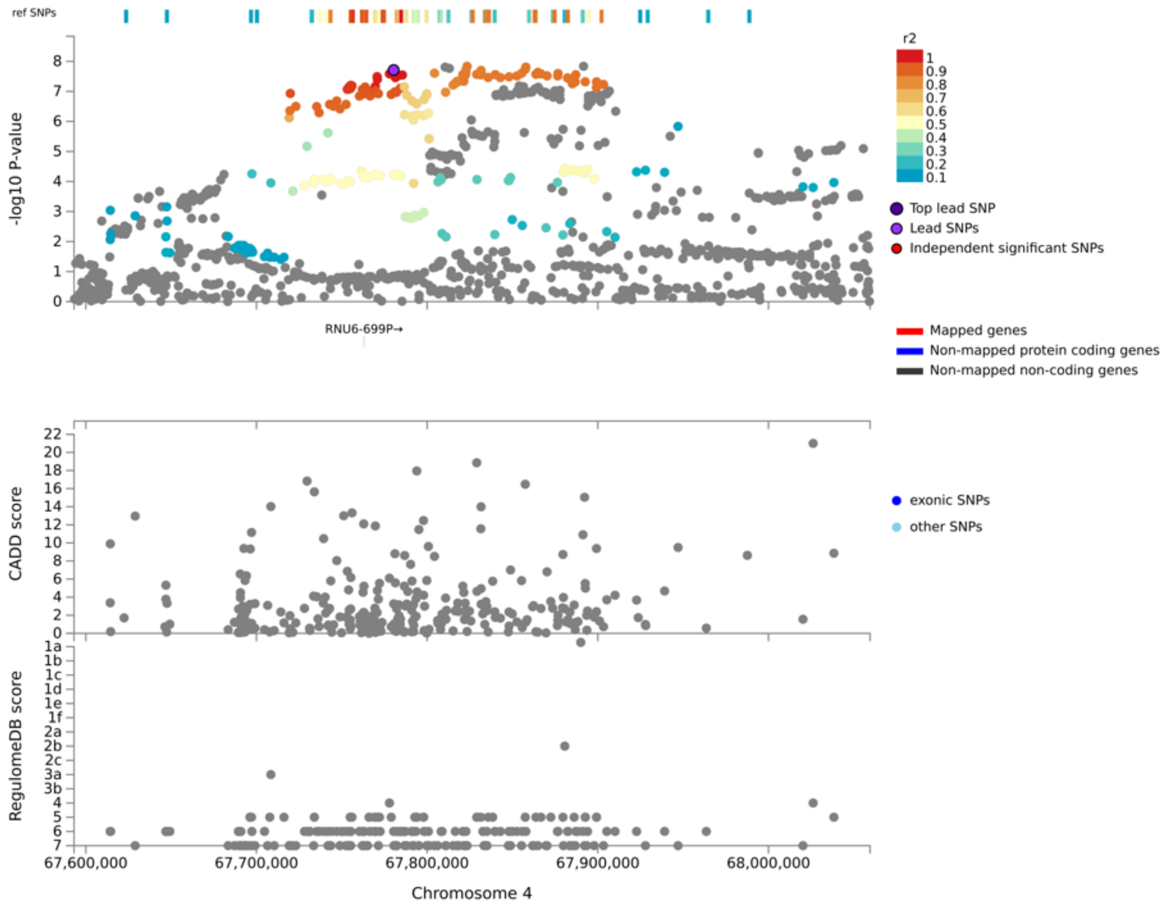

Supplementary Fig. 10. Regional association plot for SNP rs28637671.
Regional plot of the locus around the lead variant rs28637671 identified in the *mvAge* multivariate genome-wide association study (GWAS) (N=1,958,774). **Top** The top panel presents the -log$_{10}$(P-values) results of two-sided Wald tests for each variant on *mvAge* and linkage disequilibrium (LD) R^2 information for the variants in the locus (variants are colored by LD R^2) and the genes prioritized by FUMA are highlighted in red on the track below. **Bottom** The bottom panel presents the CADD (combined annotation dependent depletion) scores and RegulomeDB scores (top and bottom tracks, respectively).

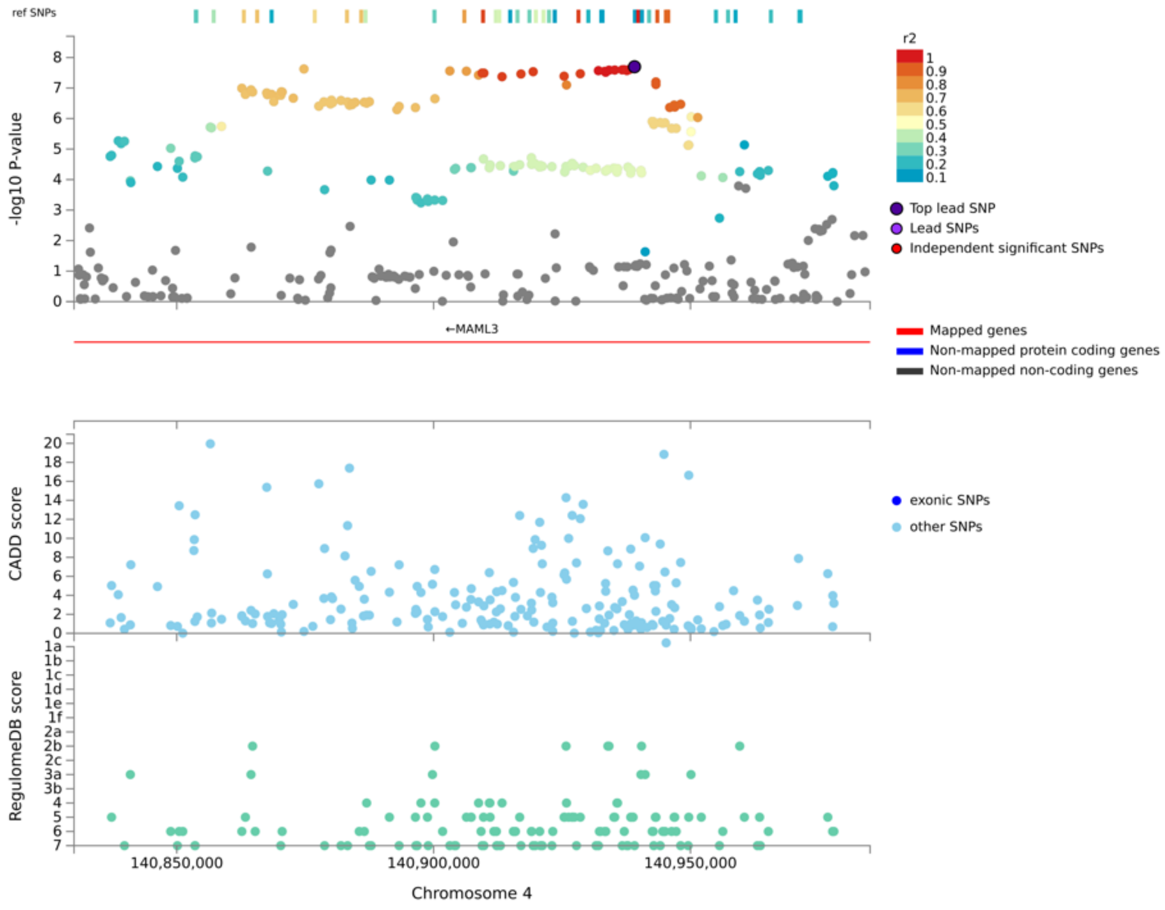

Supplementary Fig. 11. Regional association plot for SNP rs36072649.
Regional plot of the locus around the lead variant rs36072649 identified in the *mvAge* multivariate genome-wide association study (GWAS) (N=1,958,774). **Top** The top panel presents the -log10(P-values) results of two-sided Wald tests for each variant on *mvAge* and linkage disequilibrium (LD) R^2 information for the variants in the locus (variants are colored by LD R^2) and the genes prioritized by FUMA are highlighted in red on the track below. **Bottom** The bottom panel presents the CADD (combined annotation dependent depletion) scores and RegulomeDB scores (top and bottom tracks, respectively).

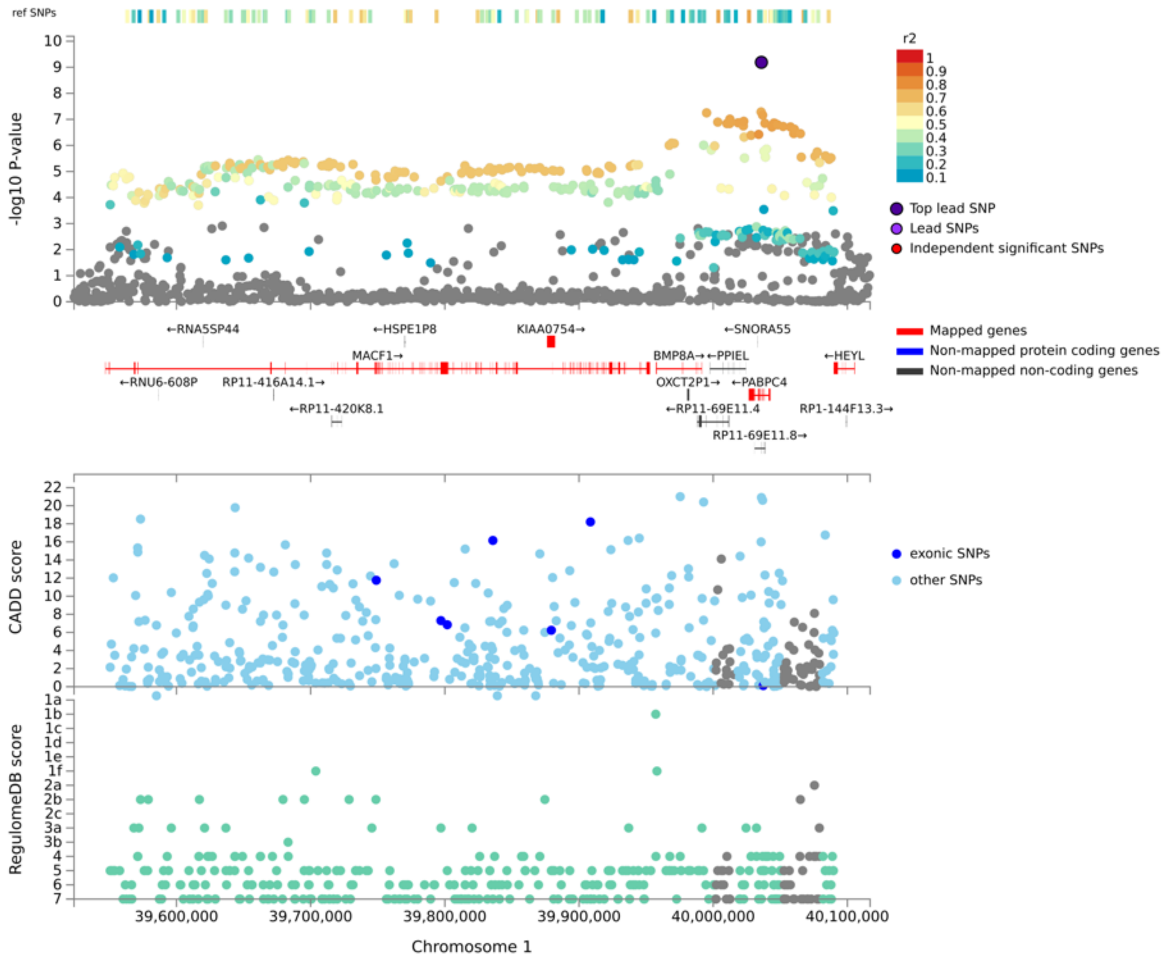

Supplementary Fig. 12. Regional association plot for SNP rs3768321.
Regional plot of the locus around the lead variant rs3768321 identified in the *mvAge* multivariate genome-wide association study (GWAS) (N=1,958,774). **Top** The top panel presents the -log10(P-values) results of two-sided Wald tests for each variant on *mvAge* and linkage disequilibrium (LD) R^2 information for the variants in the locus (variants are colored by LD r^2) and the genes prioritized by FUMA are highlighted in red on the track below. **Bottom** The bottom panel presents the CADD (combined annotation dependent depletion) scores and RegulomeDB scores (top and bottom tracks, respectively).

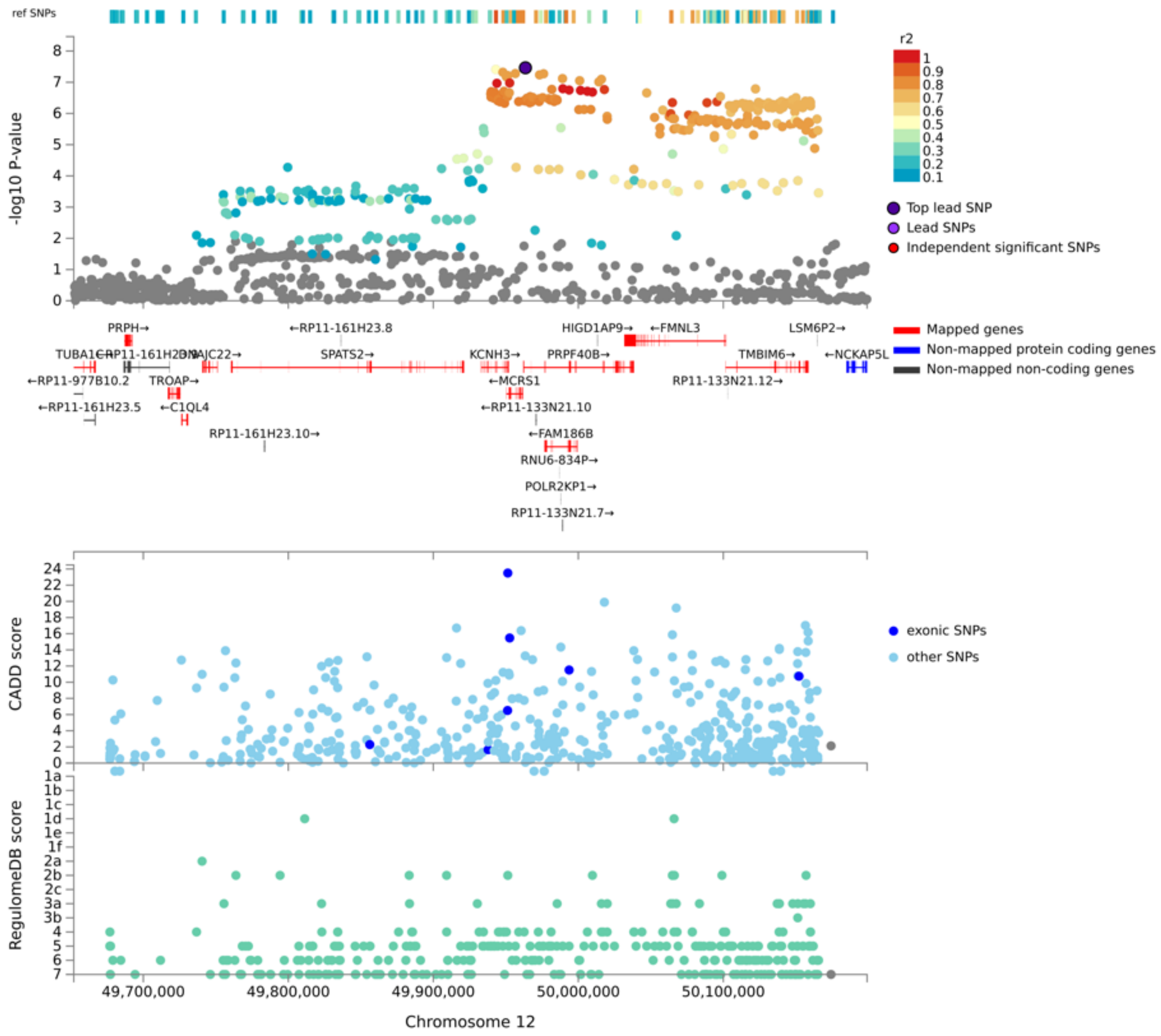

Supplementary Fig. 13. Regional association plot for SNP rs55686423.
Regional plot of the locus around the lead variant rs55686423 identified in the *mvAge* multivariate genome-wide association study (GWAS) (N=1,958,774). **Top**. The top panel presents the -log10(P-values) results of two-sided Wald tests for each variant on *mvAge* and linkage disequilibrium (LD) R^2 information for the variants in the locus (variants are colored by LD R^2) and the genes prioritized by FUMA are highlighted in red on the track below. **Bottom** The bottom panel presents the CADD (combined annotation dependent depletion) scores and RegulomeDB scores (top and bottom tracks, respectively).

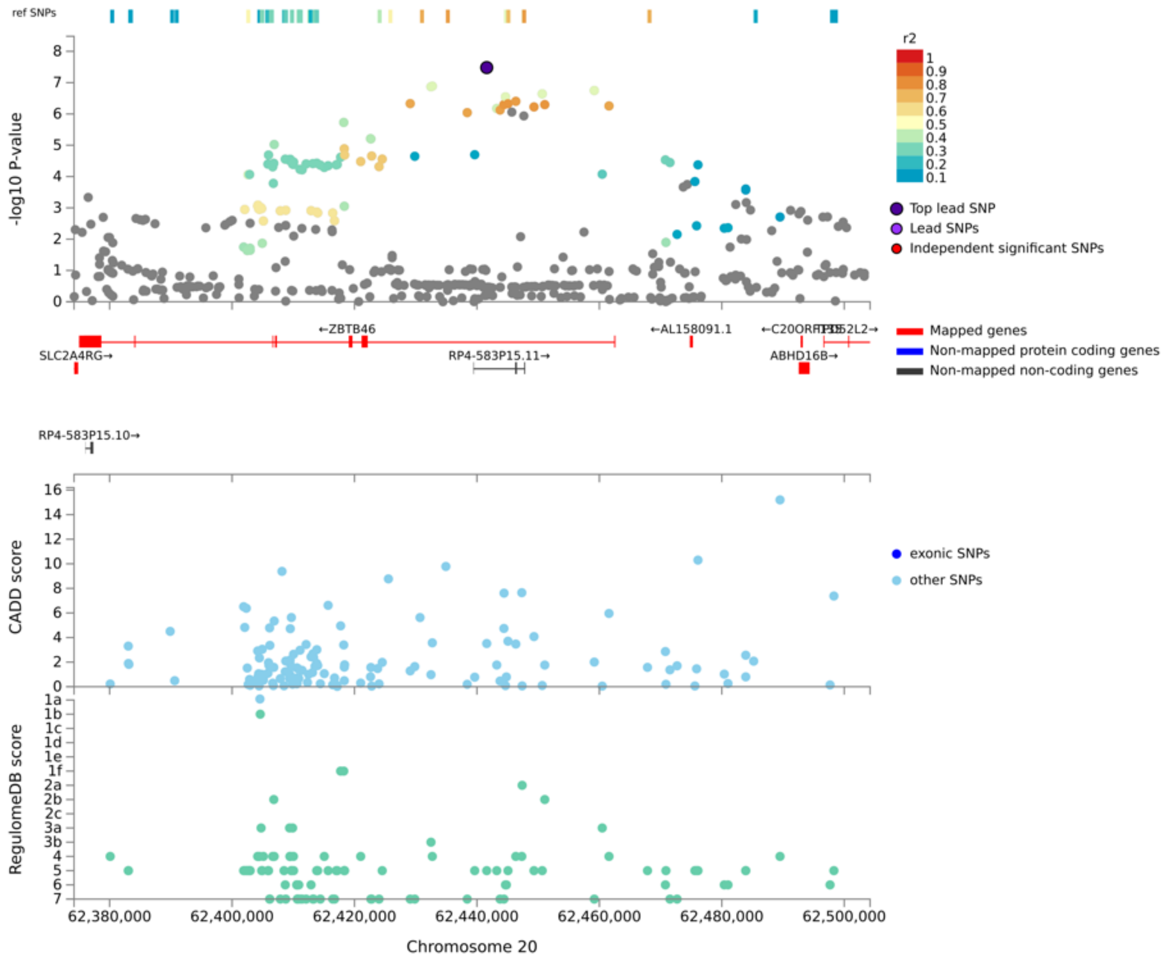

Supplementary Fig. 14. Regional association plot for SNP rs6062322.
Regional plot of the locus around the lead variant rs6062322 identified in the *mvAge* multivariate genome-wide association study (GWAS) (N=1,958,774). **Top** The top panel presents the -log10(P-values) results of two-sided Wald tests for each variant on *mvAge* and linkage disequilibrium (LD) R^2 information for the variants in the locus (variants are colored by LD R^2) and the genes prioritized by FUMA are highlighted in red on the track below. **Bottom** The bottom panel presents the CADD (combined annotation dependent depletion) scores and RegulomeDB scores (top and bottom tracks, respectively).

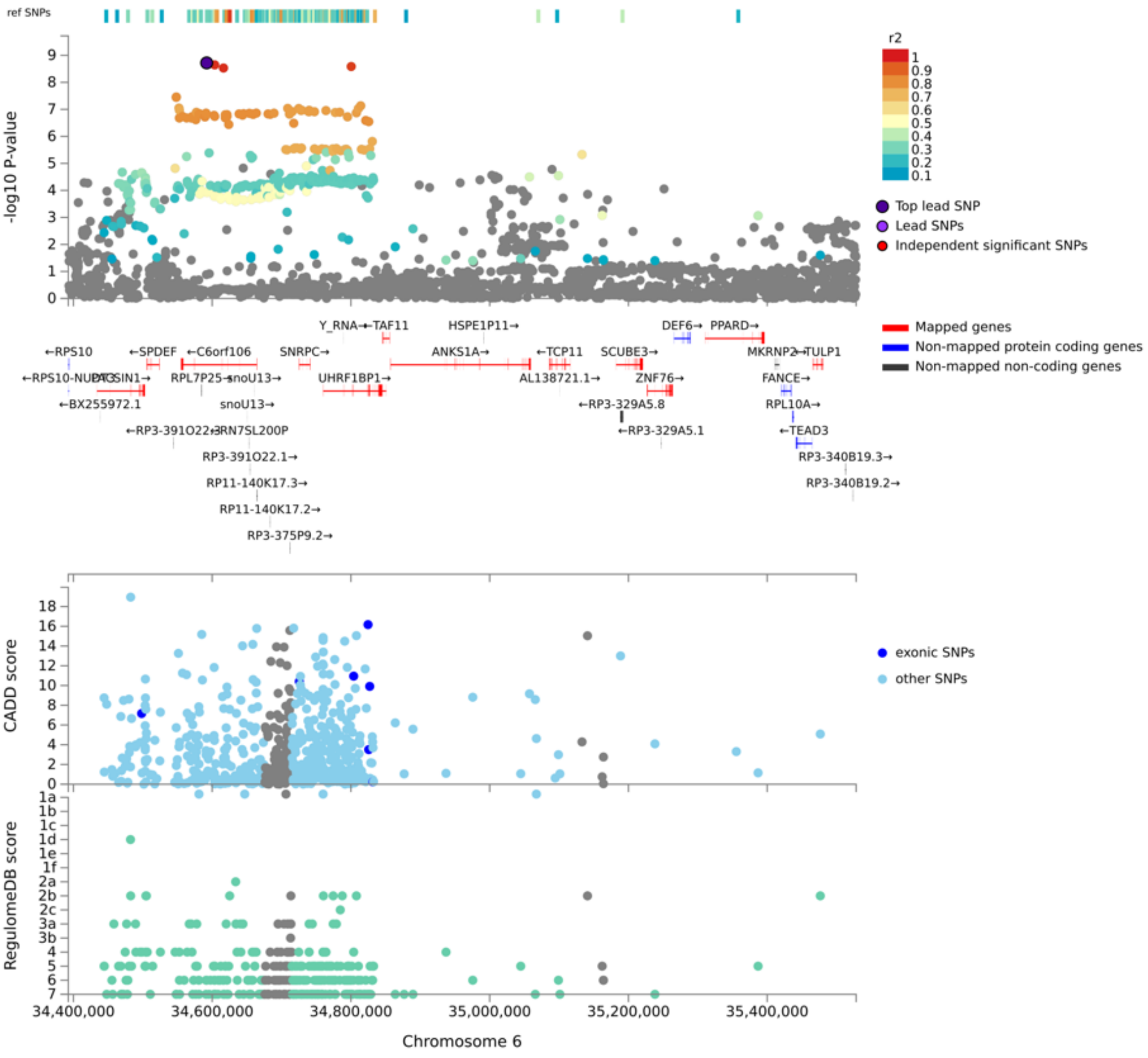

Supplementary Fig. 15. Regional association plot for SNP rs6907508.
Regional plot of the locus around the lead variant rs6907508 identified in the *mvAge* multivariate genome-wide association study (GWAS) (N=1,958,774). **Top** The top panel presents the -log10(P-values) results of two-sided Wald tests for each variant on *mvAge* and linkage disequilibrium (LD) R^2 information for the variants in the locus (variants are colored by LD R^2) and the genes prioritized by FUMA are highlighted in red on the track below. **Bottom** The bottom panel presents the CADD (combined annotation dependent depletion) scores and RegulomeDB scores (top and bottom tracks, respectively).

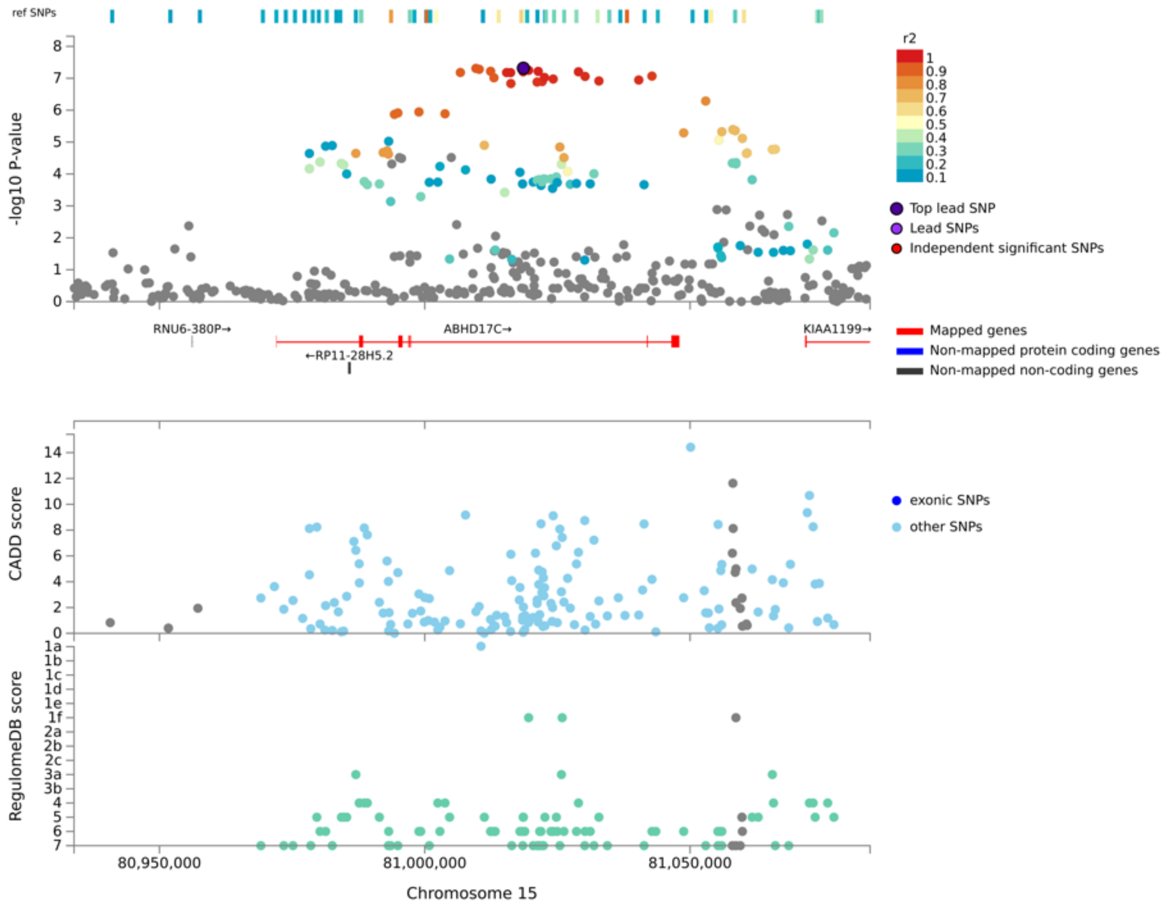

Supplementary Fig. 16. Regional association plot for SNP rs7174250.
Regional plot of the locus around the lead variant rs7174250 identified in the *mvAge* multivariate genome-wide association study (GWAS) (N=1,958,774). **Top** The top panel presents the -log10(P-values) results of two-sided Wald tests for each variant on *mvAge* and linkage disequilibrium (LD) R^2 information for the variants in the locus (variants are colored by LD R^2) and the genes prioritized by FUMA are highlighted in red on the track below. **Bottom** The bottom panel presents the CADD (combined annotation dependent depletion) scores and RegulomeDB scores (top and bottom tracks, respectively).

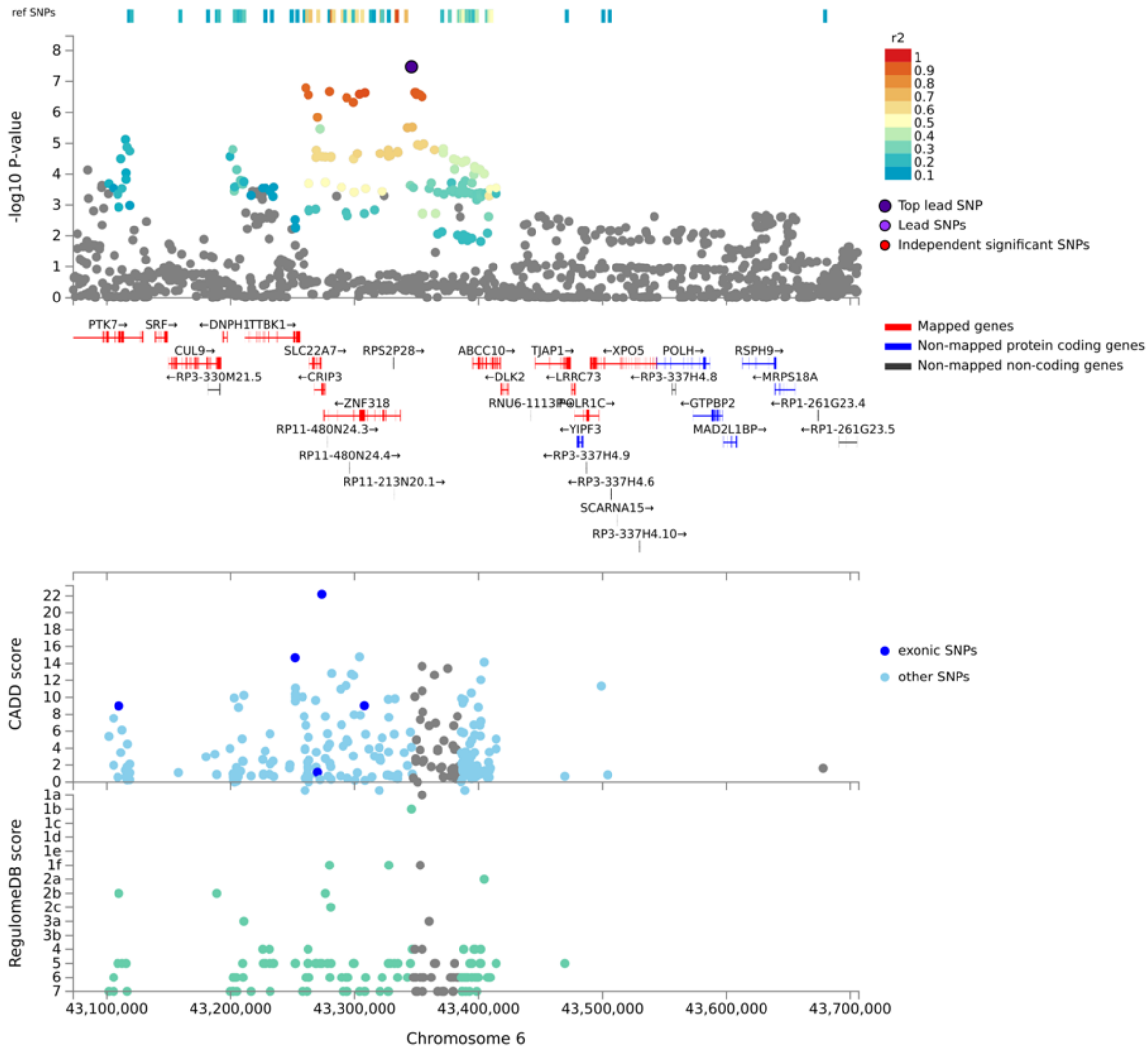

Supplementary Fig. 17. Regional association plot for SNP rs7742789.
Regional plot of the locus around the lead variant rs7742789 identified in the *mvAge* multivariate genome-wide association study (GWAS) (N=1,958,774). **Top** The top panel presents the -log10(P-values) results of two-sided Wald tests for each variant on *mvAge* and linkage disequilibrium (LD) R^2 information for the variants in the locus (variants are colored by LD R^2) and the genes prioritized by FUMA are highlighted in red on the track below. **Bottom** The bottom panel presents the CADD (combined annotation dependent depletion) scores and RegulomeDB scores (top and bottom tracks, respectively).

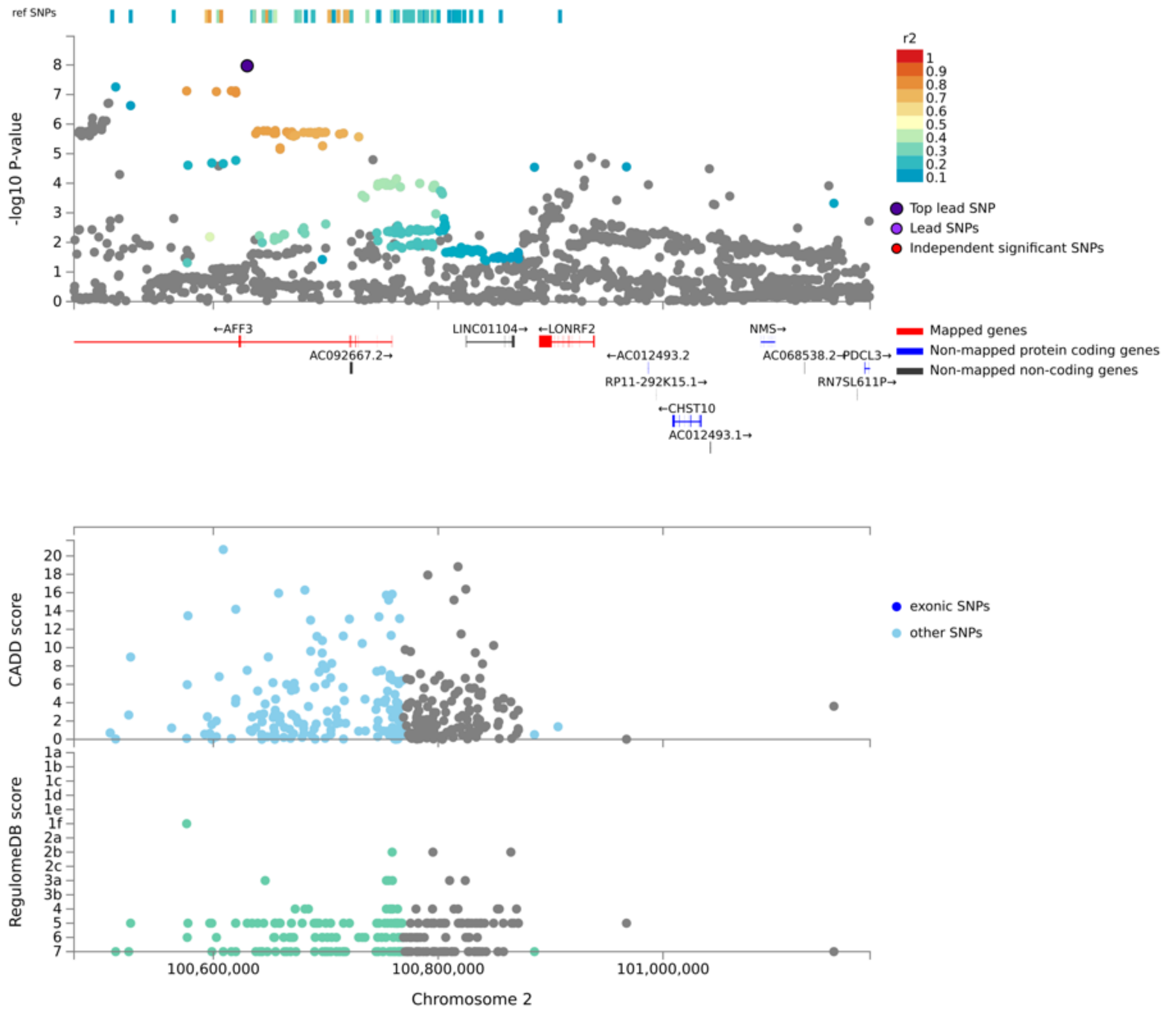

Supplementary Fig. 18. Regional association plot for SNP rs78438918.
Regional plot of the locus around the lead variant rs78438918 identified in the *mvAge* multivariate genome-wide association study (GWAS) (N=1,958,774). **Top** The top panel presents the -log10(P-values) results of two-sided Wald tests for each variant on *mvAge* and linkage disequilibrium (LD) R^2 information for the variants in the locus (variants are colored by LD R^2) and the genes prioritized by FUMA are highlighted in red on the track below. **Bottom** The bottom panel presents the CADD (combined annotation dependent depletion) scores and RegulomeDB scores (top and bottom tracks, respectively).

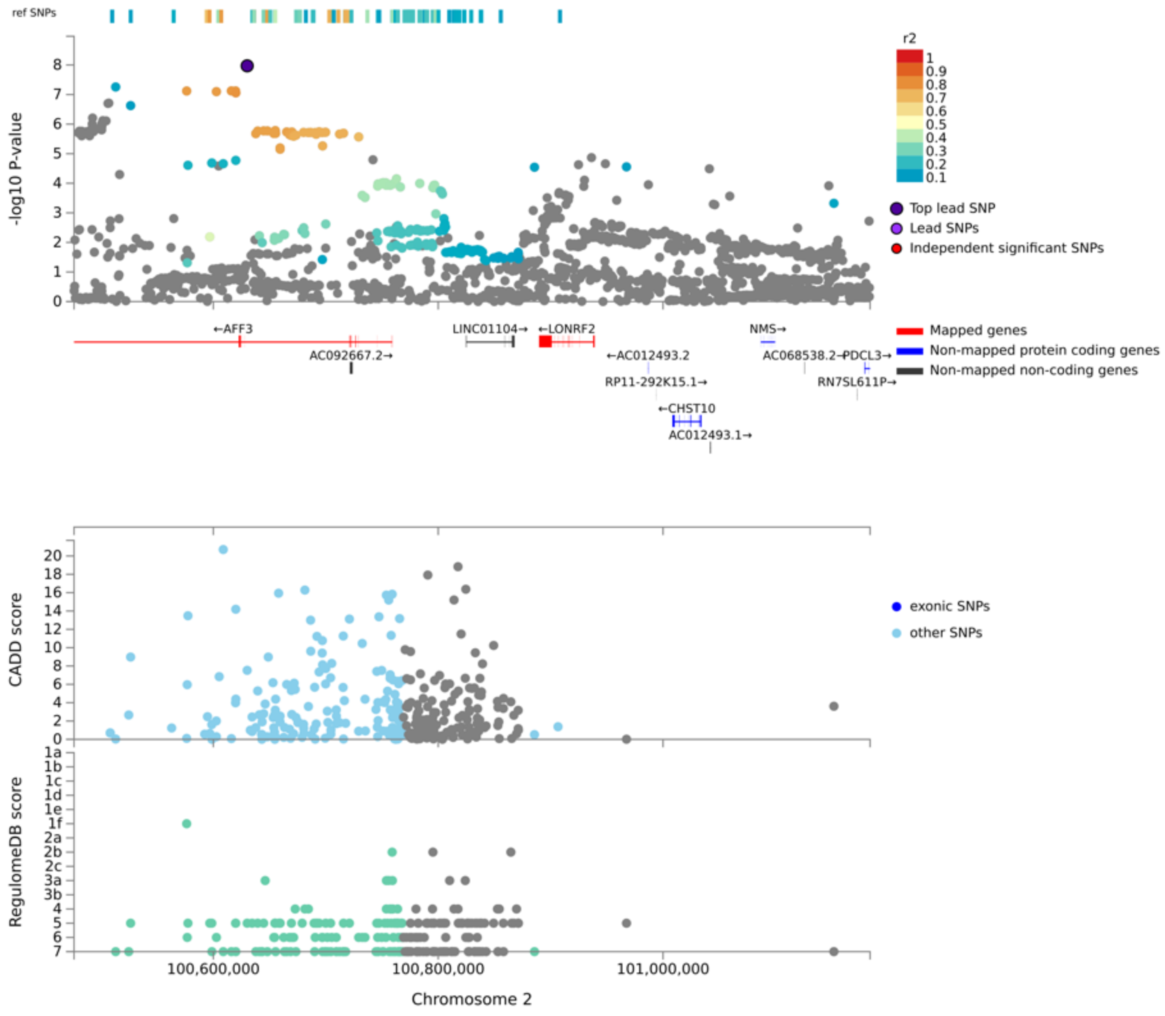

Supplementary Fig. 19. Regional association plot for SNP rs9277988.
Regional plot of the locus around the lead variant rs9277988 identified in the *mvAge* multivariate genome-wide association study (GWAS) (N=1,958,774). **Top** The top panel presents the -log10(P-values) results of two-sided Wald tests for each variant on *mvAge* and linkage disequilibrium (LD) R^2 information for the variants in the locus (variants are colored by LD R^2) and the genes prioritized by FUMA are highlighted in red on the track below. **Bottom** The bottom panel presents the CADD (combined annotation dependent depletion) scores and RegulomeDB scores (top and bottom tracks, respectively).

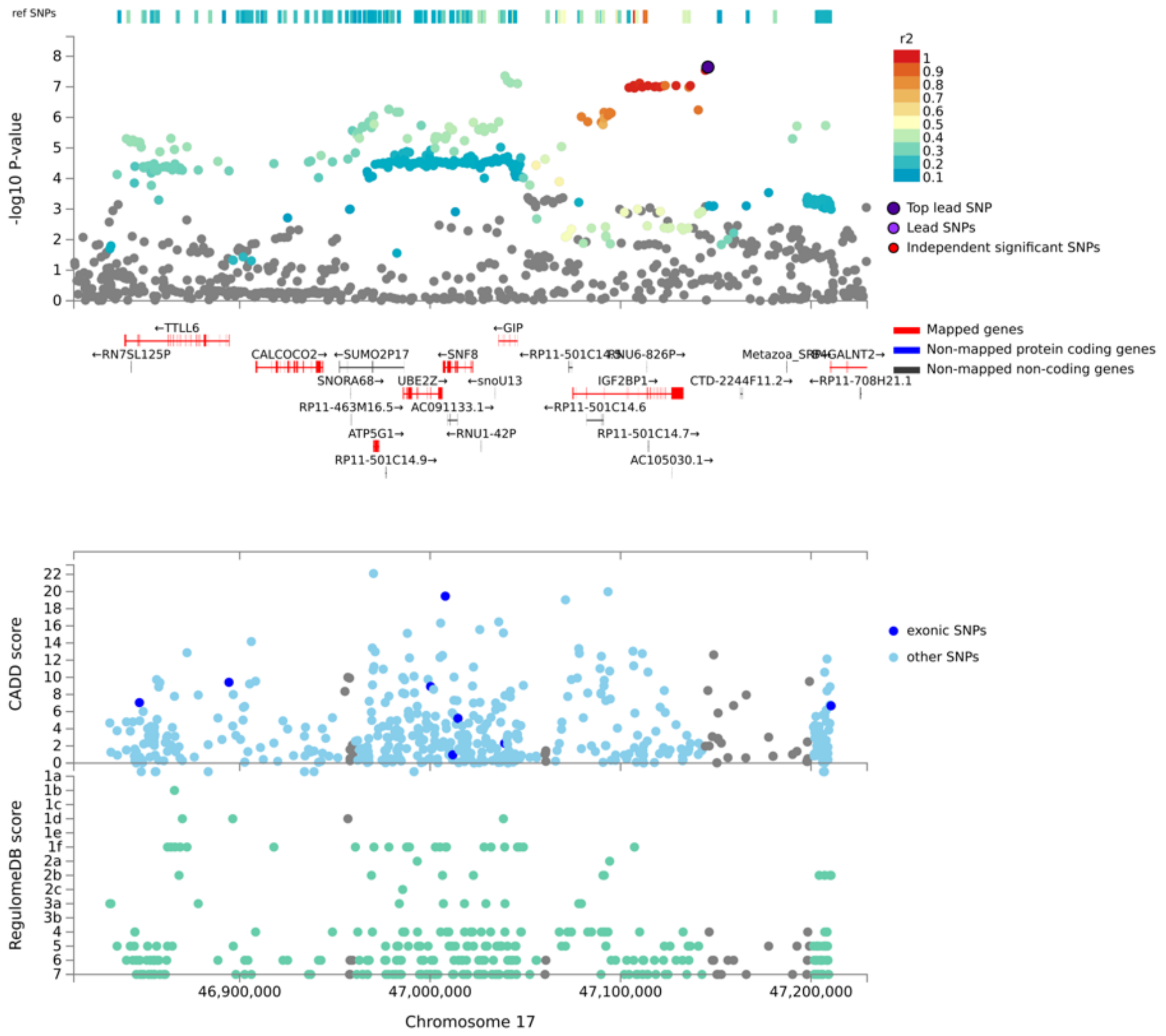

Supplementary Fig. 20. Regional association plot for SNP rs940088.
Regional plot of the locus around the lead variant rs940088 identified in the *mvAge* multivariate genome-wide association study (GWAS) (N=1,958,774). **Top** The top panel presents the -log10(P-values) results of two-sided Wald tests for each variant on *mvAge* and linkage disequilibrium (LD) R^2 information for the variants in the locus (variants are colored by LD R^2) and the genes prioritized by FUMA are highlighted in red on the track below. **Bottom** The bottom panel presents the CADD (combined annotation dependent depletion) scores and RegulomeDB scores (top and bottom tracks, respectively).

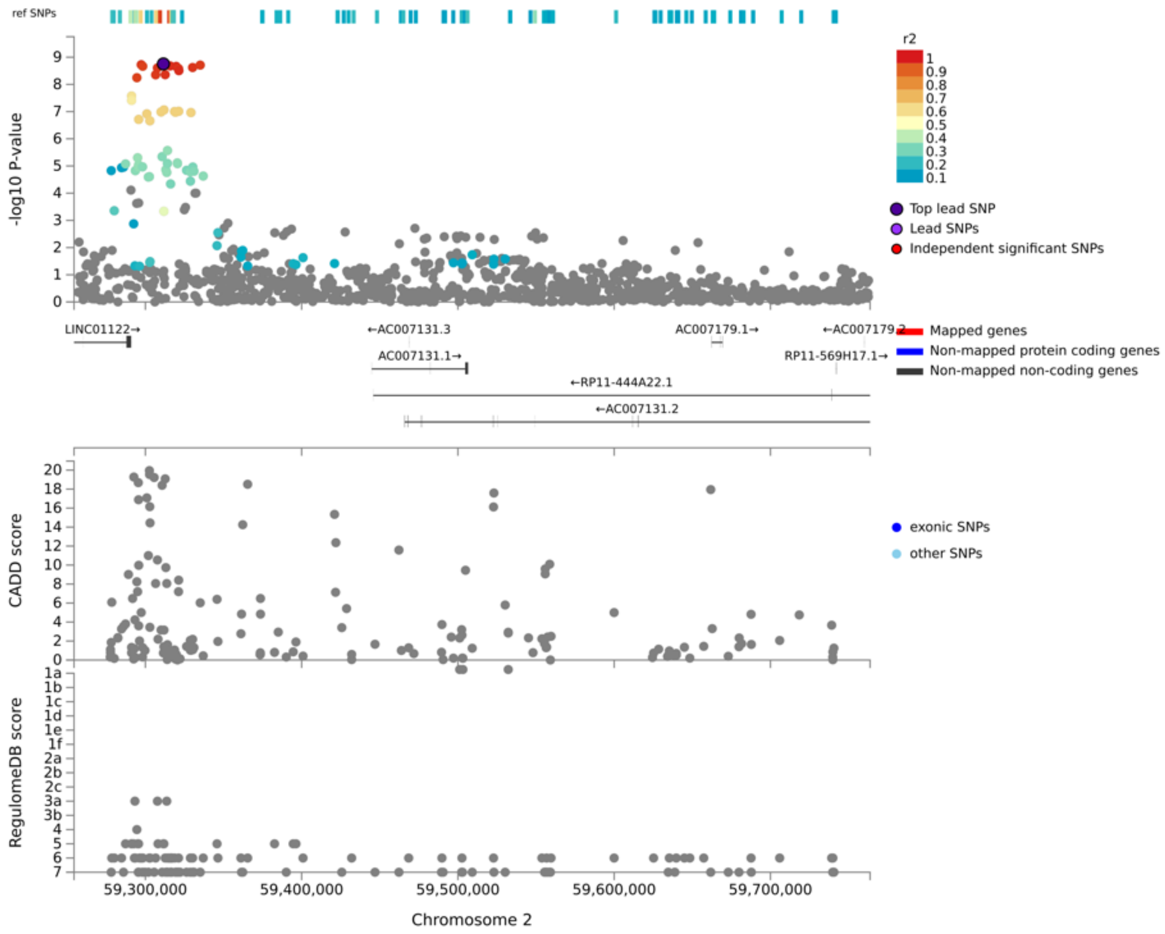

Supplementary Fig. 21. Regional association plot for SNP rs980183.
Regional plot of the locus around the lead variant rs980183 identified in the *mvAge* multivariate genome-wide association study (GWAS) (N=1,958,774). **Top** The top panel presents the -log10(P-values) results of two-sided Wald tests for each variant on *mvAge* and linkage disequilibrium (LD) R^2 information for the variants in the locus (variants are colored by LD R^2) and the genes prioritized by FUMA are highlighted in red on the track below. **Bottom** The bottom panel presents the CADD (combined annotation dependent depletion) scores and RegulomeDB scores (top and bottom tracks, respectively).

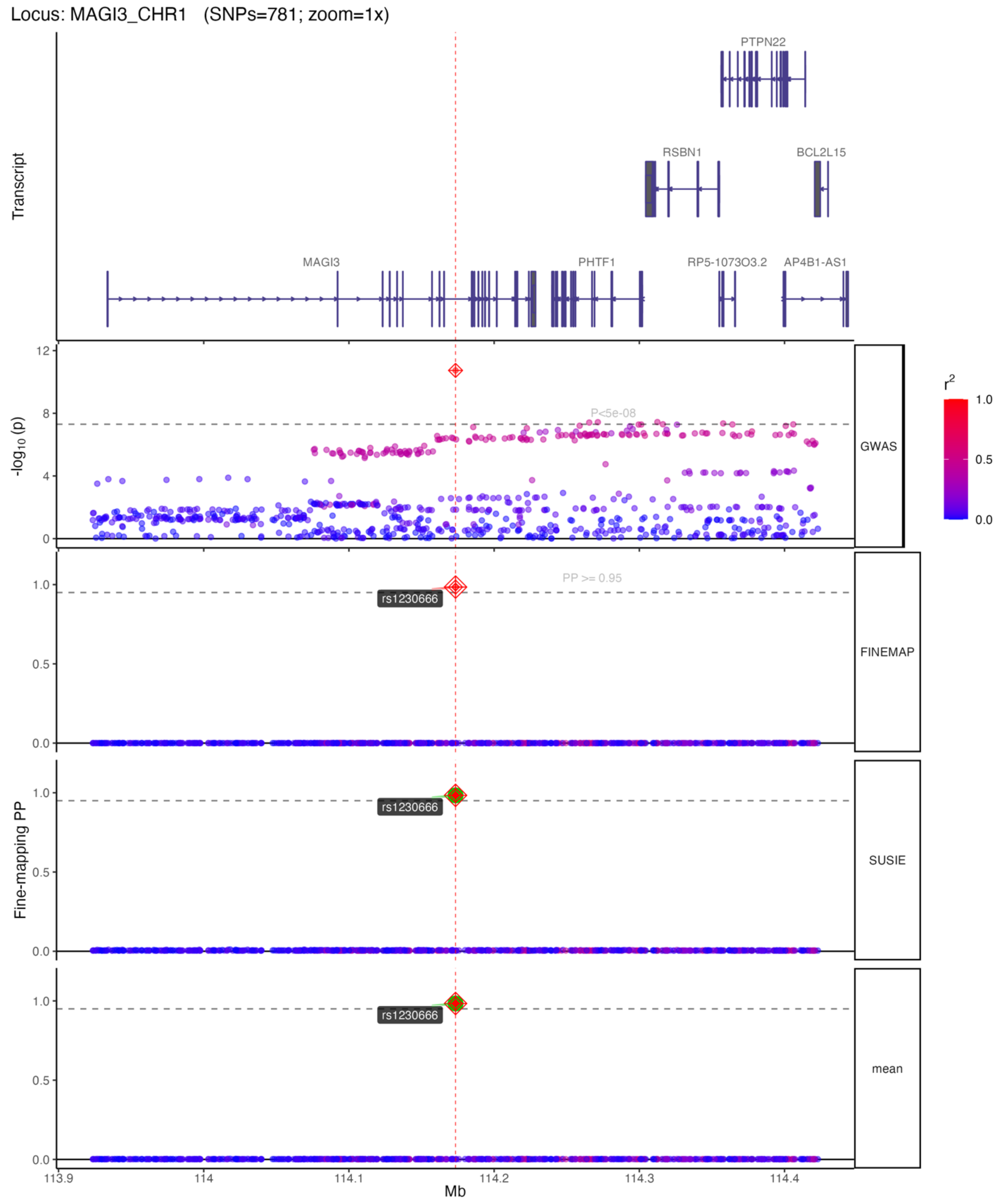

Supplementary Fig. 22. Fine mapping results of MAGI3 locus (chromosome 1).
Multitrack plot for displaying genes located within 250 kilobases of the lead variant (top panel); the regional GWAS results (-log$_{10}$(P-value) (two-sided Wald tests for each variant on *mvAge*) colored by the LD R^2 between the SNPs (second panel); the fine mapping results of the SuSIE method (third panel); fine mapping results from the FINEMAP method (fourth panel); and echolocatoR mean posterior probability (set equal to 1 when the fine mapping posterior probabilities for both SuSIE and FINEMAP *both* exceed 0.95 and is set to 0 otherwise). The vertical red line is the location of the GWAS lead SNP.

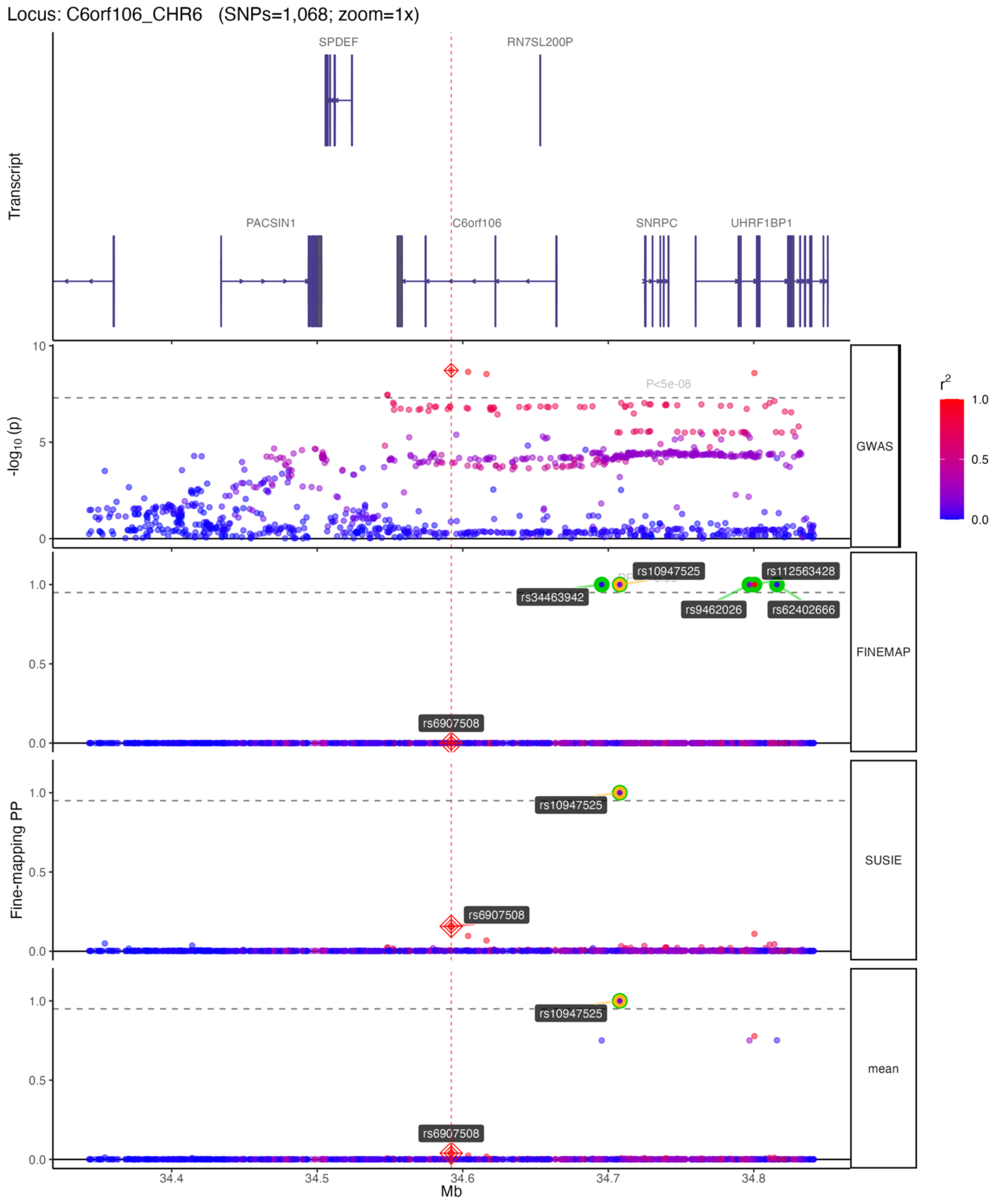

Supplementary Fig. 23. Fine mapping results of C6orf106 locus (chromosome 6).
Multitrack plot for displaying genes located within 250 kilobases of the lead variant (top panel); the regional GWAS results (-log10(P-value) (two-sided Wald tests for each variant on *mvAge*) colored by the LD R^2 between the SNPs (second panel); the fine mapping results of the SuSIE method (third panel); fine mapping results from the FINEMAP method (fourth panel); and echolocatoR mean posterior probability (set equal to 1 when the fine mapping posterior probabilities for both SuSIE and FINEMAP *both* exceed 0.95 and is set to 0 otherwise). The vertical red line is the location of the GWAS lead SNP.

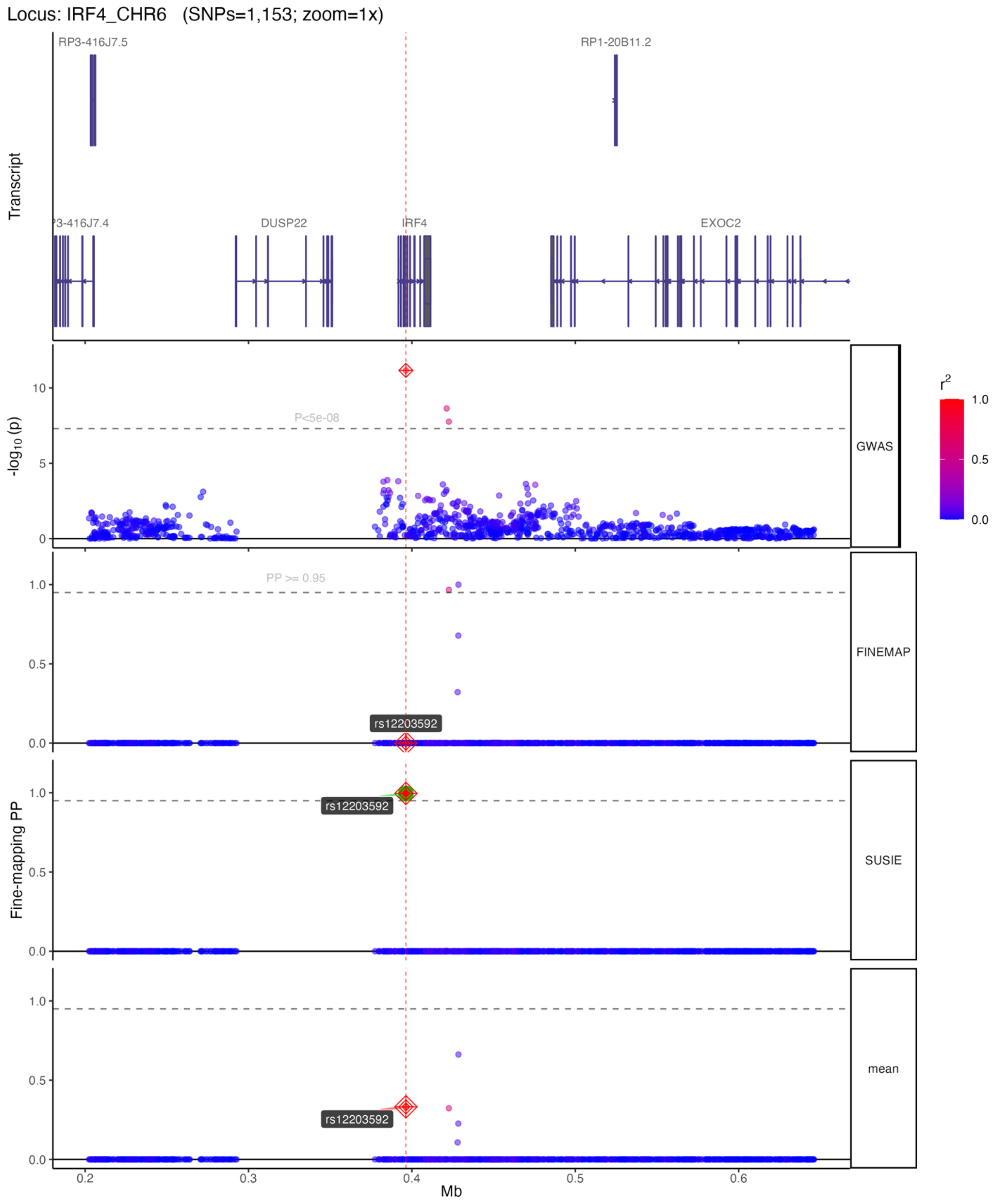

Supplementary Fig. 24. Fine mapping results of IRF4 locus (chromosome 6).
Multitrack plot for displaying genes located within 250 kilobases of the lead variant (top panel); the regional GWAS results (-$\log_{10}$(P-value) (two-sided Wald tests for each variant on *mvAge*) colored by the LD R^2 between the SNPs (second panel); the fine mapping results of the SuSIE method (third panel); fine mapping results from the FINEMAP method (fourth panel); and echolocatoR mean posterior probability (set equal to 1 when the fine mapping posterior probabilities for both SuSIE and FINEMAP *both* exceed 0.95 and is set to 0 otherwise). The vertical red line is the location of the GWAS lead SNP.

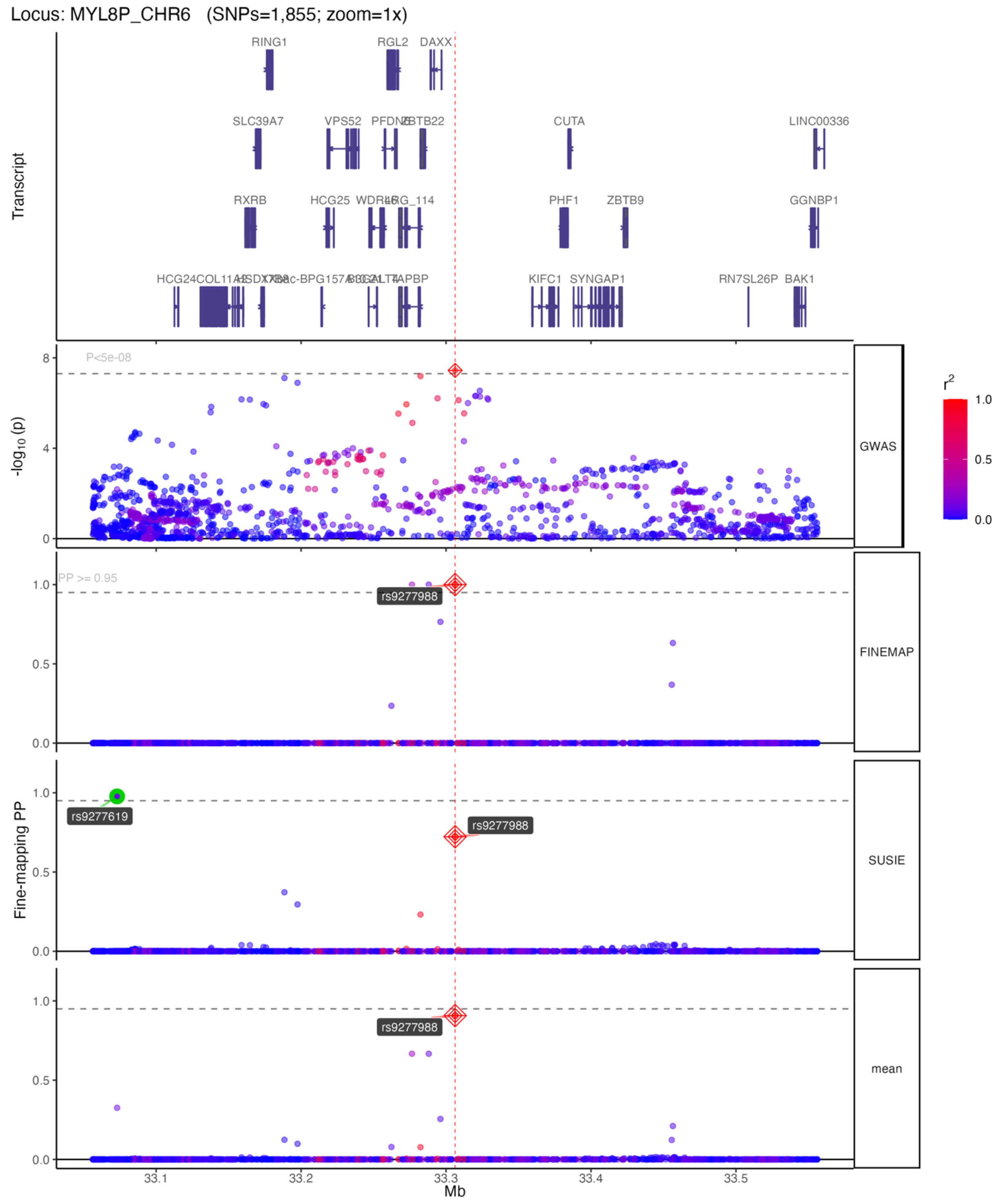

Supplementary Fig. 25. Fine mapping results of MYL8P locus (chromosome 6).
Multitrack plot for displaying genes located within 250 kilobases of the lead variant (top panel); the regional GWAS results (-log10(P-value) (two-sided Wald tests for each variant on *mvAge*) colored by the LD R^2 between the SNPs (second panel); the fine mapping results of the SuSIE method (third panel); fine mapping results from the FINEMAP method (fourth panel); and echolocator mean posterior probability (set equal to 1 when the fine mapping posterior probabilities for both SuSIE and FINEMAP *both* exceed 0.95 and is set to 0 otherwise). The vertical red line is the location of the GWAS lead SNP.

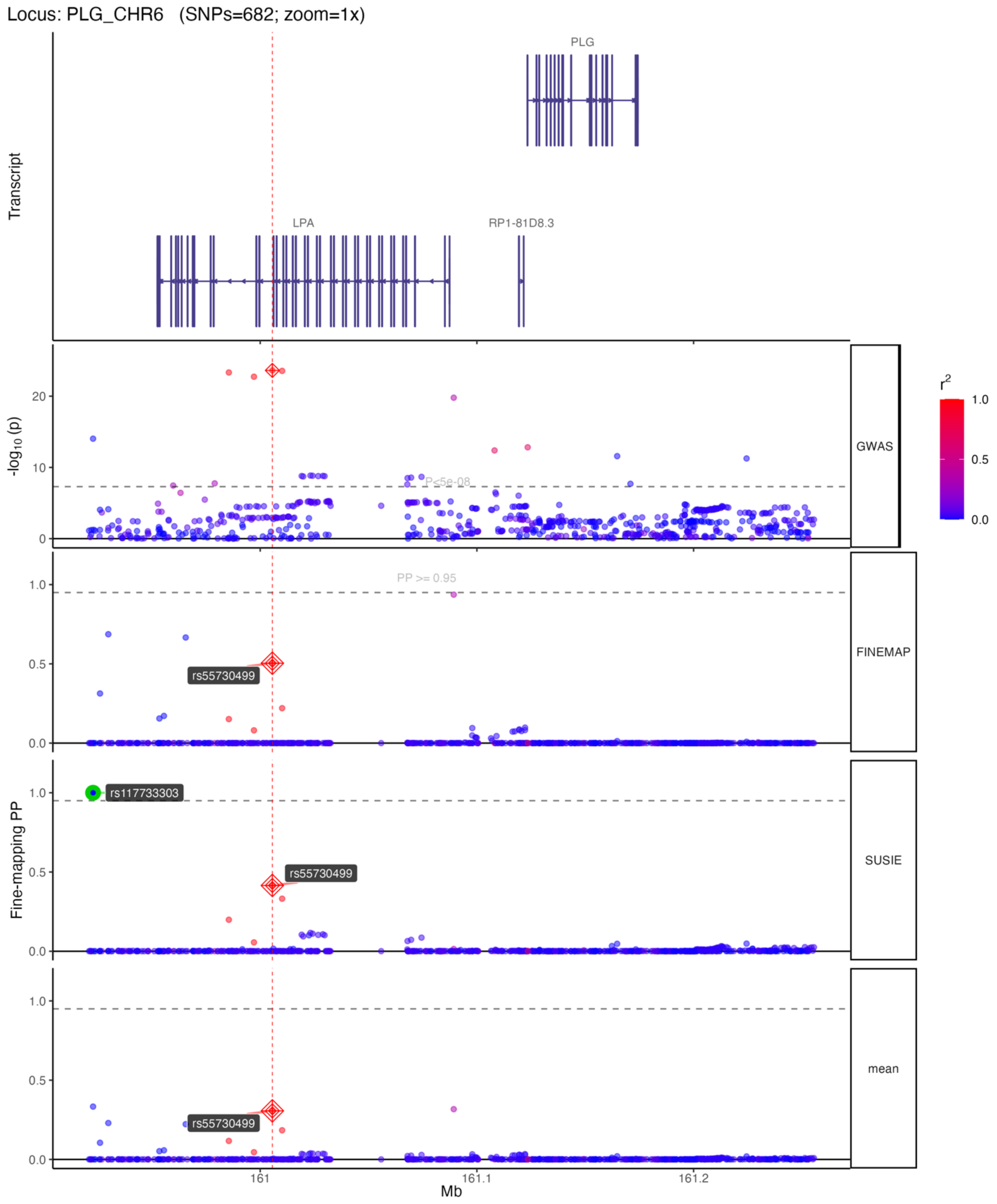

Supplementary Fig. 26. Fine mapping results of PLG locus (chromosome 6).
Multitrack plot for displaying genes located within 250 kilobases of the lead variant (top panel); the regional GWAS results (-log$_{10}$(P-value) (two-sided Wald tests for each variant on *mvAge*) colored by the LD R^2 between the SNPs (second panel); the fine mapping results of the SuSIE method (third panel); fine mapping results from the FINEMAP method (fourth panel); and echolocator mean posterior probability (set equal to 1 when the fine mapping posterior probabilities for both SuSIE and FINEMAP *both* exceed 0.95 and is set to 0 otherwise). The vertical red line is the location of the GWAS lead SNP.

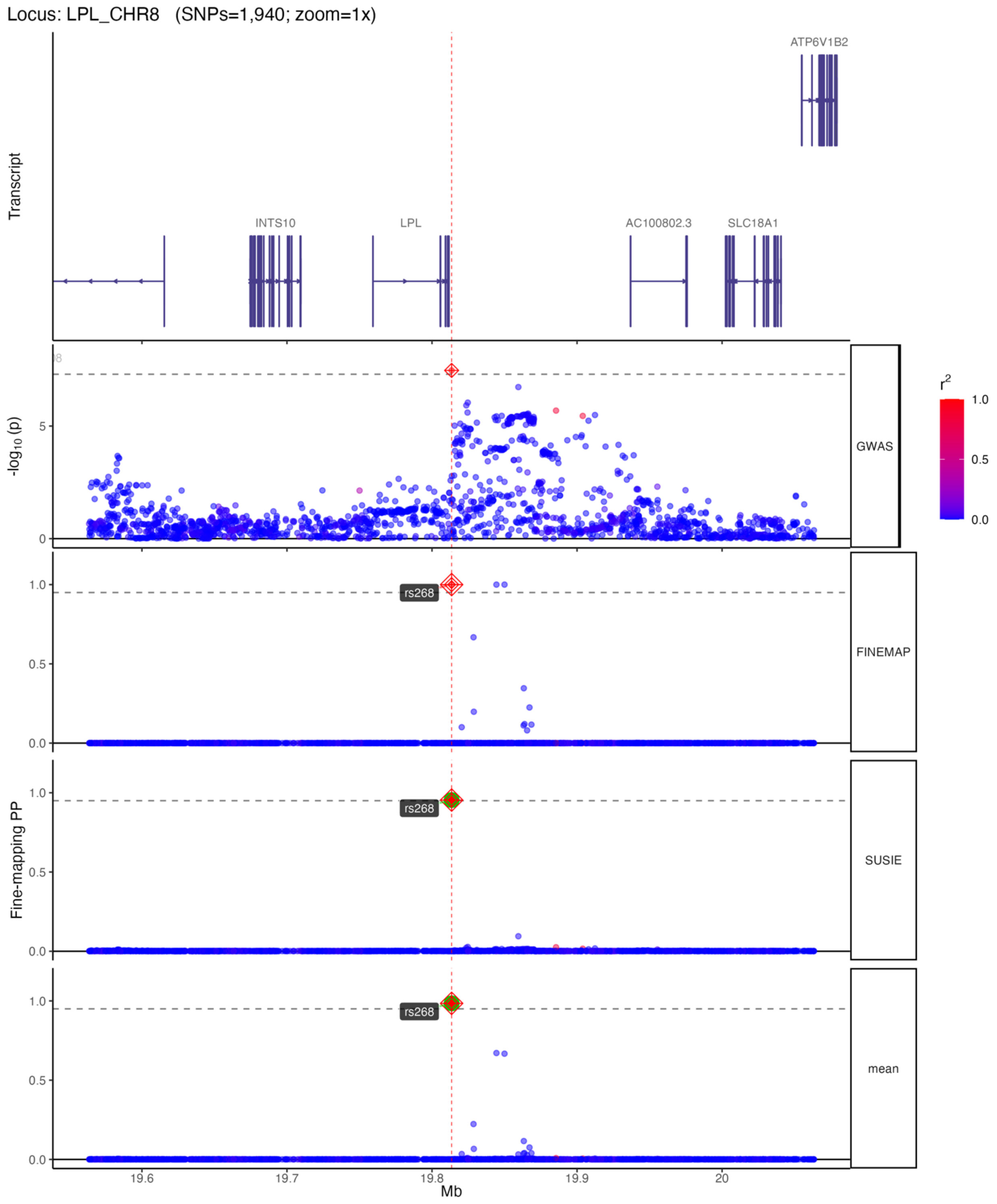

Supplementary Fig. 27. Fine mapping results of LPL locus (chromosome 8).
Multitrack plot for displaying genes located within 250 kilobases of the lead variant (top panel); the regional GWAS results ($-\log_{10}$(P-value) (two-sided Wald tests for each variant on *mvAge*) colored by the LD R^2 between the SNPs (second panel); the fine mapping results of the SuSIE method (third panel); fine mapping results from the FINEMAP method (fourth panel); and echolocatoR mean posterior probability (set equal to 1 when the fine mapping posterior probabilities for both SuSIE and FINEMAP *both* exceed 0.95 and is set to 0 otherwise). The vertical red line is the location of the GWAS lead SNP.

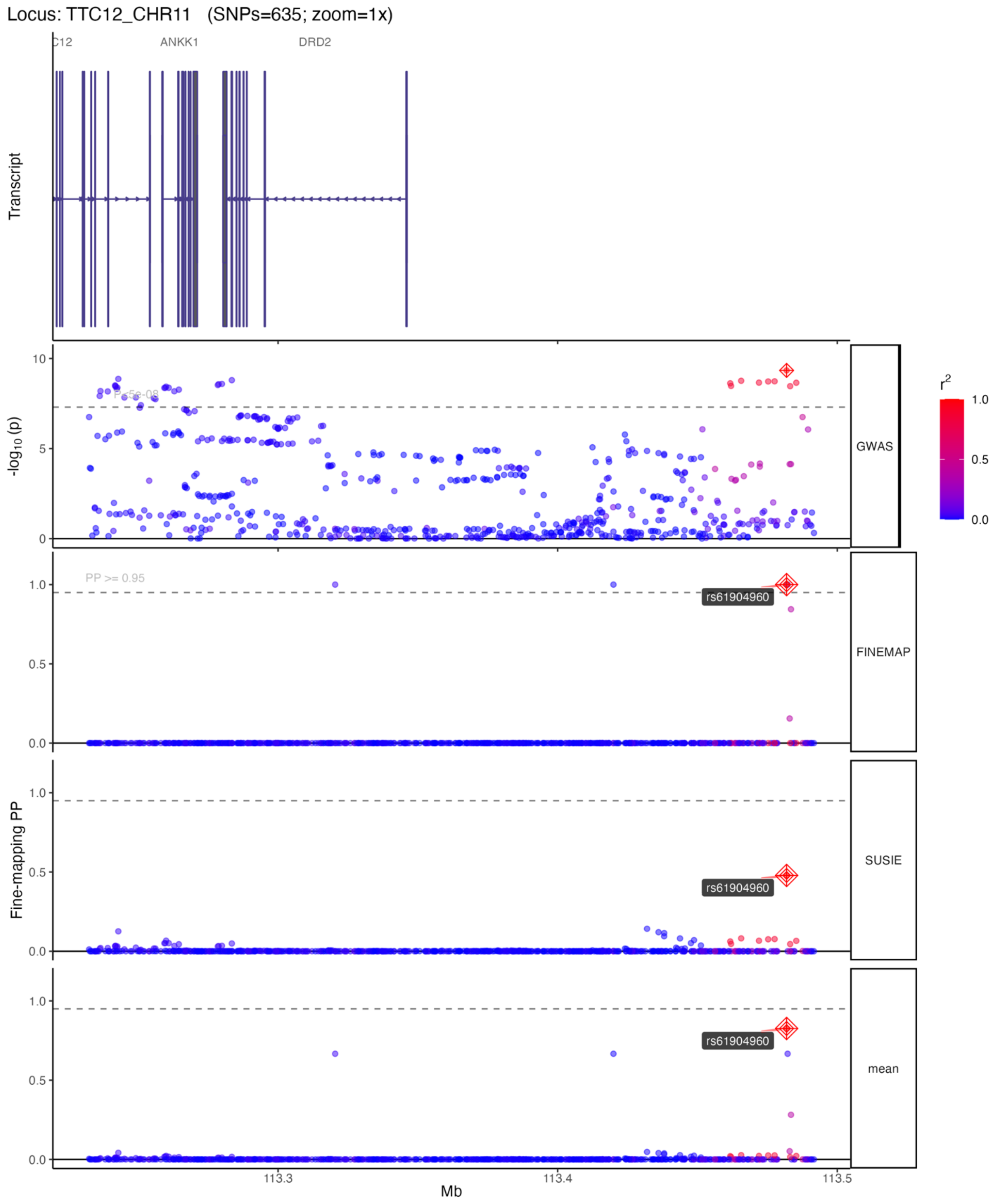

Supplementary Fig. 28. Fine mapping results of TTC12 locus (chromosome 11).
Multitrack plot for displaying genes located within 250 kilobases of the lead variant (top panel); the regional GWAS results (-log10(P-value) (two-sided Wald tests for each variant on *mvAge*) colored by the LD R^2 between the SNPs (second panel); the fine mapping results of the SuSIE method (third panel); fine mapping results from the FINEMAP method (fourth panel); and echolocator mean posterior probability (set equal to 1 when the fine mapping posterior probabilities for both SuSIE and FINEMAP *both* exceed 0.95 and is set to 0 otherwise). The vertical red line is the location of the GWAS lead SNP.

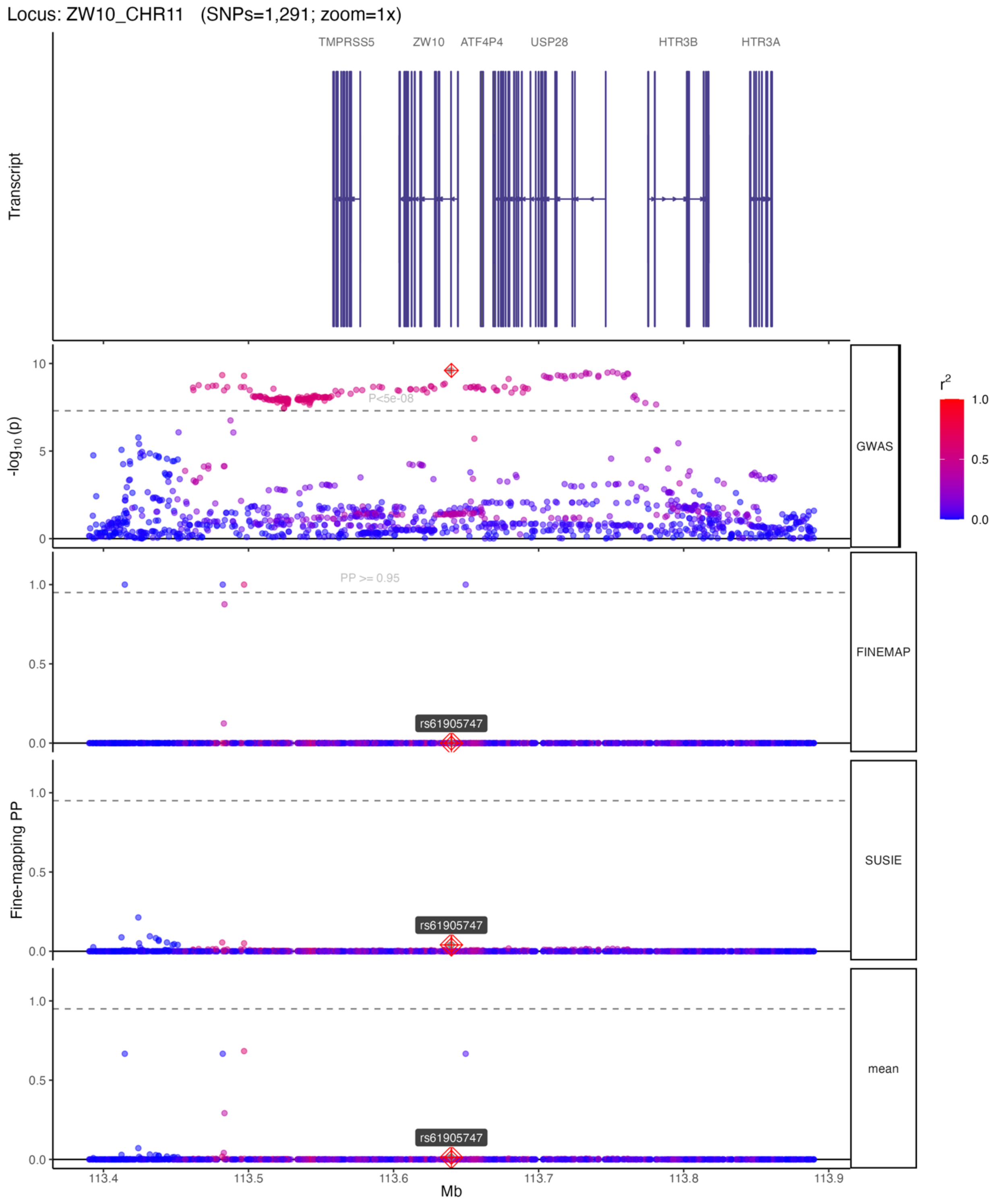

Supplementary Fig. 29. Fine mapping results of ZW10 locus (chromosome 11).
Multitrack plot for displaying genes located within 250 kilobases of the lead variant (top panel); the regional GWAS results (-log10(P-value) (two-sided Wald tests for each variant on *mvAge*) colored by the LD R^2 between the SNPs (second panel); the fine mapping results of the SuSIE method (third panel); fine mapping results from the FINEMAP method (fourth panel); and echolocatoR mean posterior probability (set equal to 1 when the fine mapping posterior probabilities for both SuSIE and FINEMAP *both* exceed 0.95 and is set to 0 otherwise). The vertical red line is the location of the GWAS lead SNP.

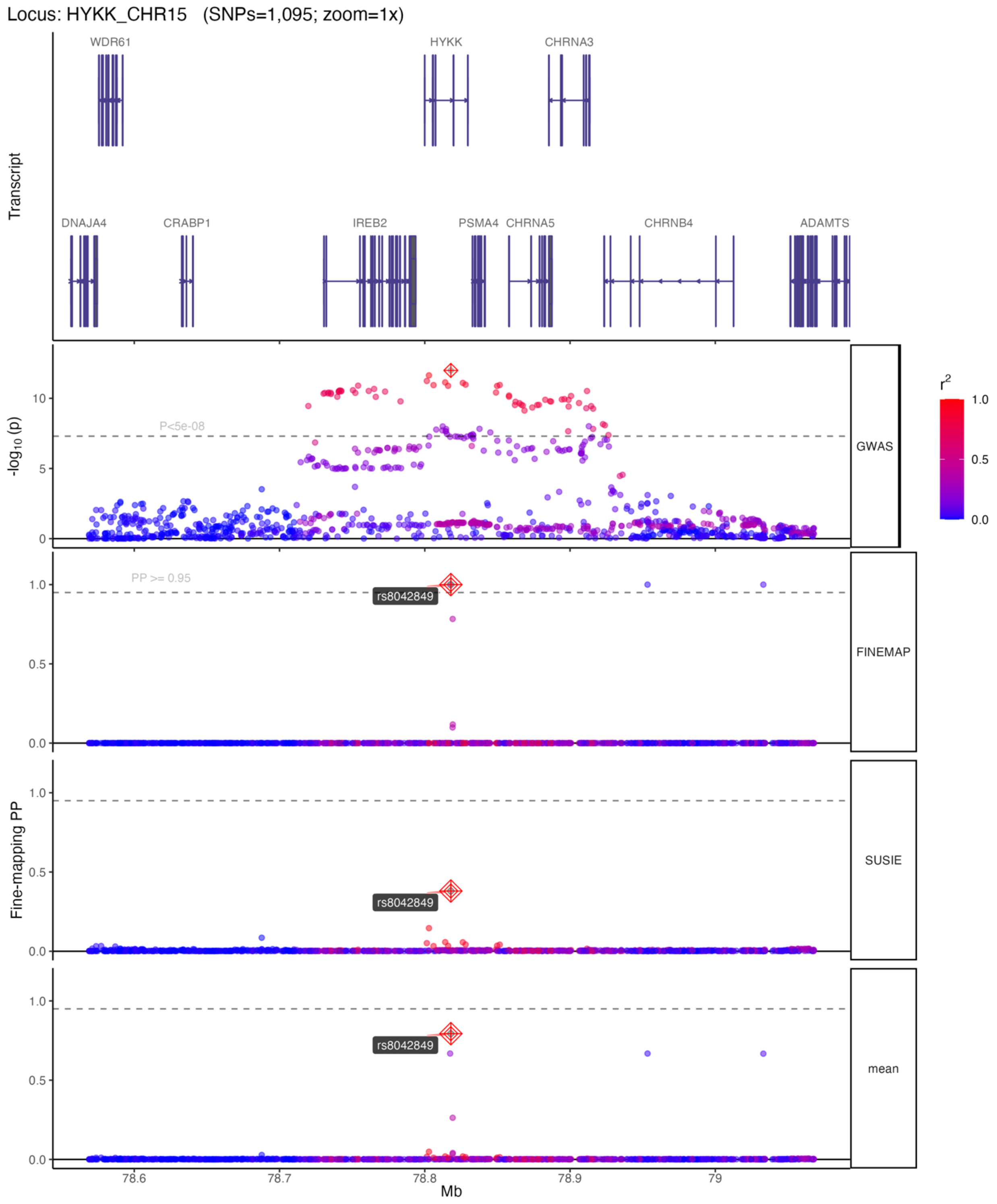

Supplementary Fig. 30. Fine mapping results of HYKK locus (chromosome 15). Multitrack plot for displaying genes located within 250 kilobases of the lead variant (top panel); the regional GWAS results (-log10(P-value) (two-sided Wald tests for each variant on *mvAge*) colored by the LD R^2 between the SNPs (second panel); the fine mapping results of the SuSIE method (third panel); fine mapping results from the FINEMAP method (fourth panel); and echolocatoR mean posterior probability (set equal to 1 when the fine mapping posterior probabilities for both SuSIE and FINEMAP *both* exceed 0.95 and is set to 0 otherwise). The vertical red line is the location of the GWAS lead SNP.

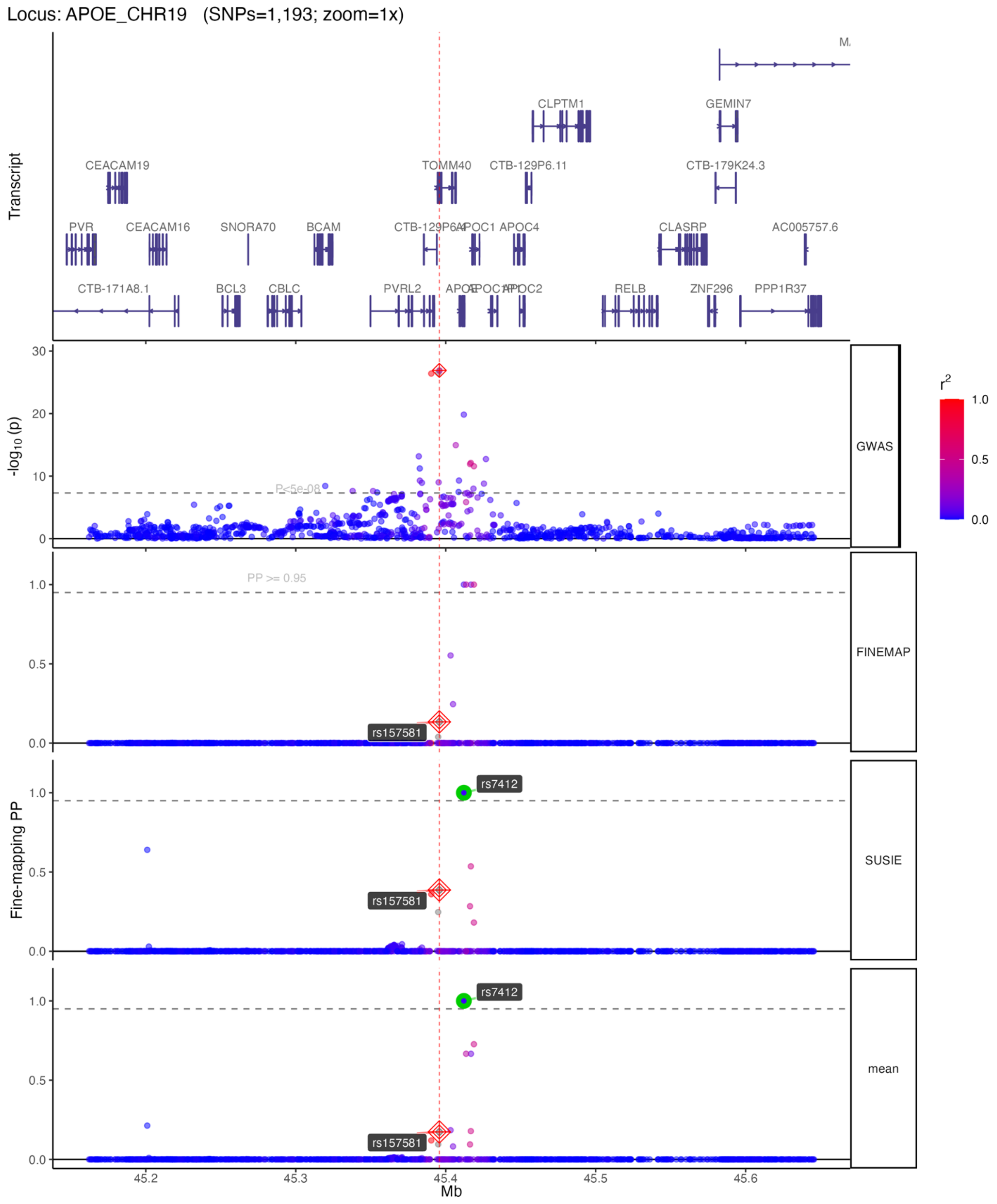

Supplementary Fig. 31. Fine mapping results of APOE locus (chromosome 19). Multitrack plot for displaying genes located within 250 kilobases of the lead variant (top panel); the regional GWAS results (-$\log_{10}$(P-value) (two-sided Wald tests for each variant on *mvAge*) colored by the LD R^2 between the SNPs (second panel); the fine mapping results of the SuSIE method (third panel); fine mapping results from the FINEMAP method (fourth panel); and echolocatoR mean posterior probability (set equal to 1 when the fine mapping posterior probabilities for both SuSIE and FINEMAP *both* exceed 0.95 and is set to 0 otherwise). The vertical red line is the location of the GWAS lead SNP.

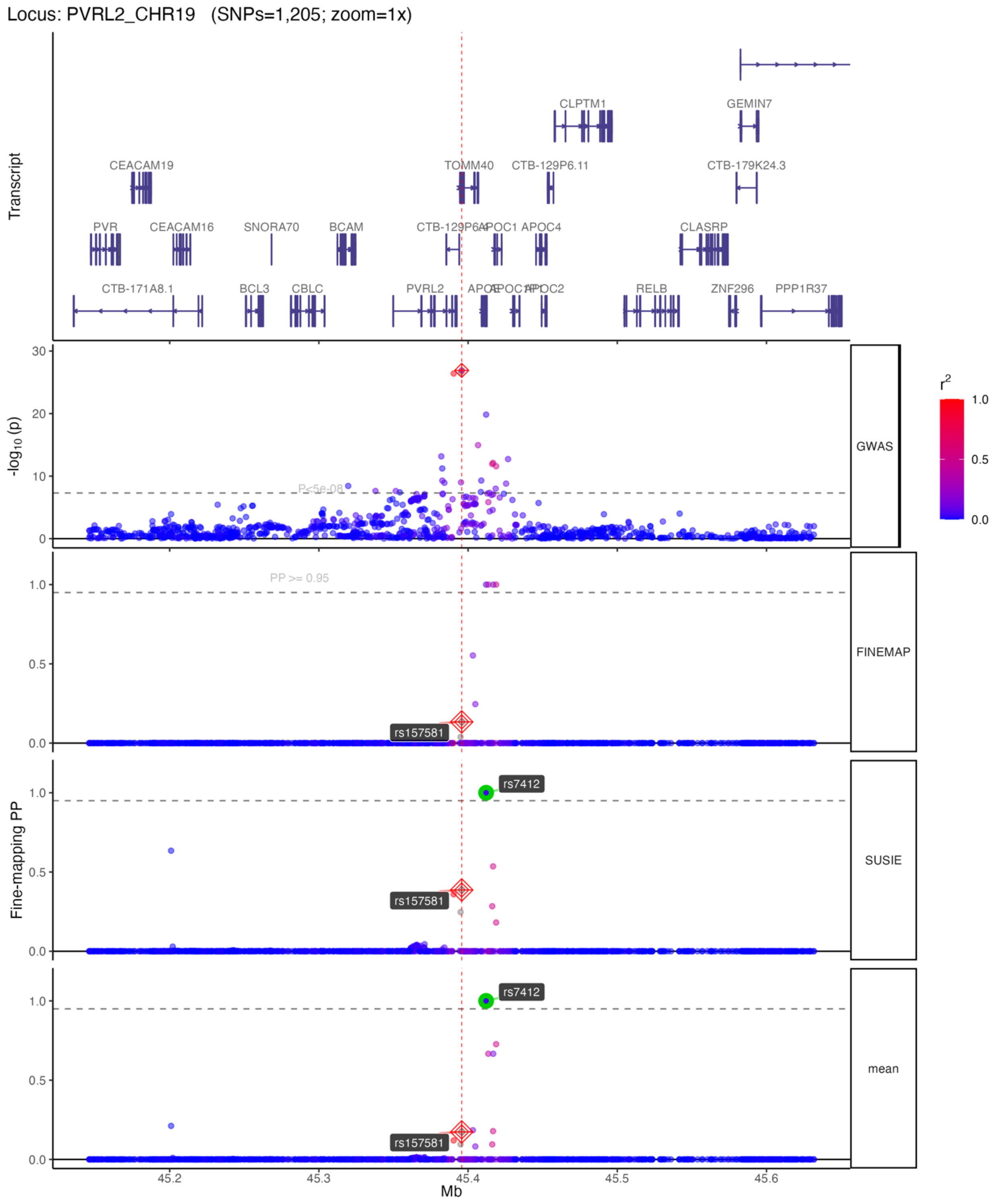

Supplementary Fig. 32. Fine mapping results of PVRL2 locus (chromosome 19).
Multitrack plot for displaying genes located within 250 kilobases of the lead variant (top panel); the regional GWAS results (-$\log_{10}$(P-value) (two-sided Wald tests for each variant on *mvAge*) colored by the LD R^2 between the SNPs (second panel); the fine mapping results of the SuSIE method (third panel); fine mapping results from the FINEMAP method (fourth panel); and echolocatoR mean posterior probability (set equal to 1 when the fine mapping posterior probabilities for both SuSIE and FINEMAP *both* exceed 0.95 and is set to 0 otherwise). The vertical red line is the location of the GWAS lead SNP.

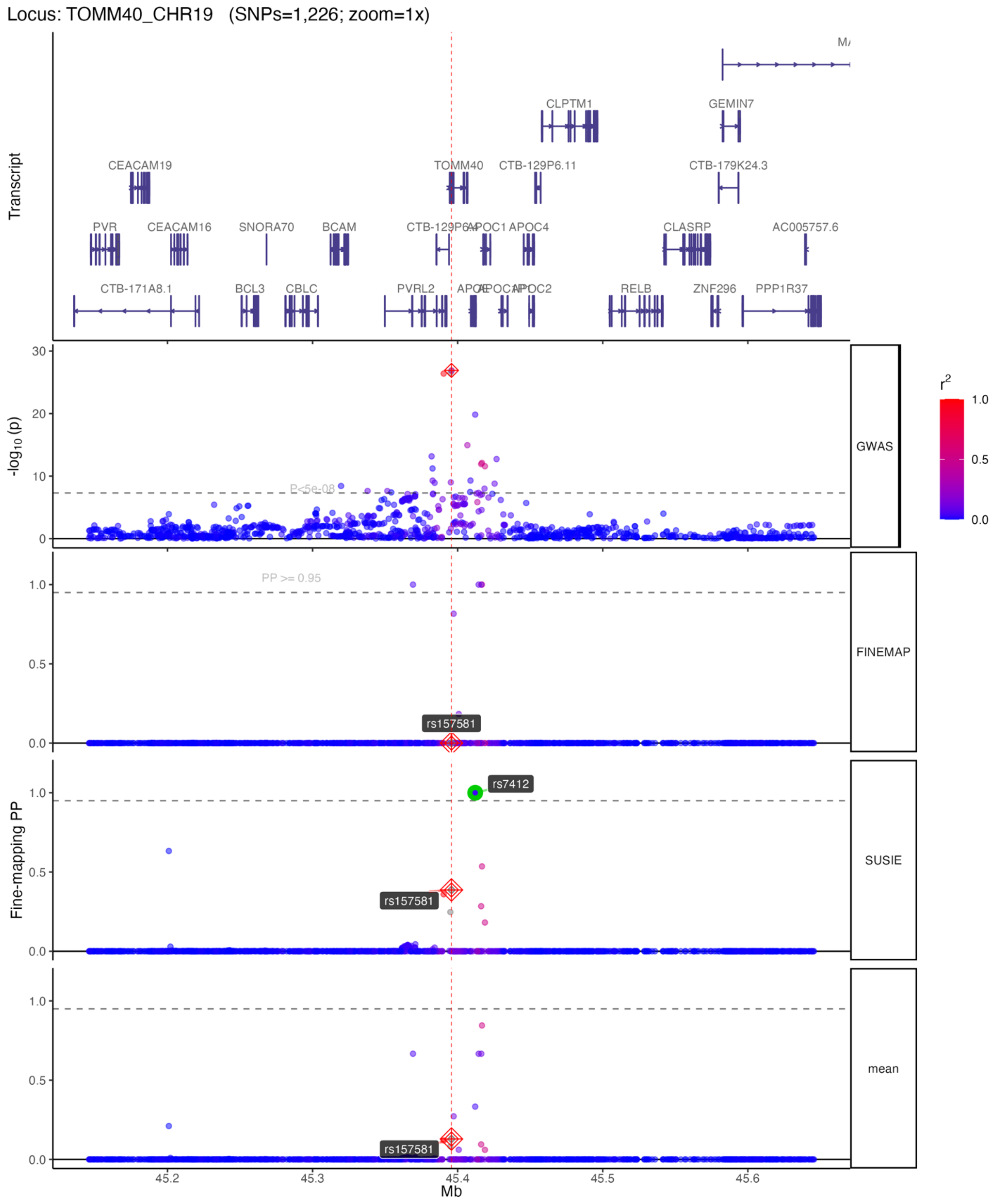

Supplementary Fig. 33. Fine mapping results of TOMM40 locus (chromosome 19).
Multitrack plot for displaying genes located within 250 kilobases of the lead variant (top panel); the regional GWAS results (-$\log_{10}$(P-value) (two-sided Wald tests for each variant on *mvAge*) colored by the LD R^2 between the SNPs (second panel); the fine mapping results of the SuSIE method (third panel); fine mapping results from the FINEMAP method (fourth panel); and echolocatoR mean posterior probability (set equal to 1 when the fine mapping posterior probabilities for both SuSIE and FINEMAP *both* exceed 0.95 and is set to 0 otherwise). The vertical red line is the location of the GWAS lead SNP.

REFERENCES

1. Folkersen L, Gustafsson S, Wang Q, et al. Genomic and drug target evaluation of 90 cardiovascular proteins in 30,931 individuals. *Nat Metab.* 2020;2(10):1135-1148.
2. Lawlor DA, Tilling K, Davey Smith G. Triangulation in aetiological epidemiology. *International Journal of Epidemiology.* 2016;45(6):1866-1886.
3. Onken B, Driscoll M. Metformin Induces a Dietary Restriction–Like State and the Oxidative Stress Response to Extend C. elegans Healthspan via AMPK, LKB1, and SKN-1. *PLOS ONE.* 2010;5(1):e8758.
4. Slack C, Foley A, Partridge L. Activation of AMPK by the putative dietary restriction mimetic metformin is insufficient to extend lifespan in Drosophila. *PLoS One.* 2012;7(10):e47699.
5. Martin-Montalvo A, Mercken EM, Mitchell SJ, et al. Metformin improves healthspan and lifespan in mice. *Nature Communications.* 2013;4(1):2192.
6. Sanderson E, Glymour MM, Holmes MV, et al. Mendelian randomization. *Nature Reviews Methods Primers.* 2022;2(1):6.
7. Schmidt AF, Finan C, Gordillo-Marañón M, et al. Genetic drug target validation using Mendelian randomisation. *Nature communications.* 2020;11(1):3255-3255.
8. Wang C, Chen B, Feng Q, Nie C, Li T. Clinical perspectives and concerns of metformin as an anti-aging drug. *Aging Med (Milton).* 2020;3(4):266-275.
9. Evans JMM, Donnelly LA, Emslie-Smith AM, Alessi DR, Morris AD. Metformin and reduced risk of cancer in diabetic patients. *BMJ.* 2005;330(7503):1304.
10. Libby G, Donnelly LA, Donnan PT, Alessi DR, Morris AD, Evans JM. New users of metformin are at low risk of incident cancer: a cohort study among people with type 2 diabetes. *Diabetes Care.* 2009;32(9):1620-1625.
11. Chen HP, Shieh JJ, Chang CC, et al. Metformin decreases hepatocellular carcinoma risk in a dose-dependent manner: population-based and in vitro studies. *Gut.* 2013;62(4):606-615.
12. Suissa S, Azoulay L. Metformin and the risk of cancer: time-related biases in observational studies. *Diabetes Care.* 2012;35(12):2665-2673.
13. Medicine AECo. Metformin in Longevity Study (MILES). (MILES) . https://clinicaltrials.gov/ct2/show/NCT02432287. Accessed July 5 2022.
14. Barzilai N, Crandall JP, Kritchevsky SB, Espeland MA. Metformin as a Tool to Target Aging. *Cell Metab.* 2016;23(6):1060-1065.
15. Xie K, Chen CH, Tsai SP, et al. Loss of Life Expectancy by 10 Years or More From Elevated Aspartate Aminotransferase: Finding Aspartate Aminotransferase a Better Mortality Predictor for All-Cause and Liver-Related than Alanine Aminotransferase. *Am J Gastroenterol.* 2019;114(9):1478-1487.
16. Richardson TG, Wang Q, Sanderson E, et al. Effects of apolipoprotein B on lifespan and risks of major diseases including type 2 diabetes: a mendelian randomisation analysis using outcomes in first-degree relatives. *The Lancet Healthy Longevity.* 2021;2(6):e317-e326.
17. Shaw M, Mitchell R, Dorling D. Time for a smoke? One cigarette reduces your life by 11 minutes. *BMJ (Clinical research ed).* 2000;320(7226):53-53.

18. Tam J, Warner KE, Meza R. Smoking and the Reduced Life Expectancy of Individuals With Serious Mental Illness. *American Journal of Preventive Medicine.* 2016;51(6):958-966.
19. Case A, Deaton A. Life expectancy in adulthood is falling for those without a BA degree, but as educational gaps have widened, racial gaps have narrowed. *Proceedings of the National Academy of Sciences.* 2021;118(11):e2024777118.
20. Zhou H, Zhang Y, Liu J, et al. Education and lung cancer: a Mendelian randomization study. *International Journal of Epidemiology.* 2019;48(3):743-750.
21. Rosoff DB, Davey Smith G, Mehta N, Clarke TK, Lohoff FW. Evaluating the relationship between alcohol consumption, tobacco use, and cardiovascular disease: A multivariable Mendelian randomization study. *PLoS Med.* 2020;17(12):e1003410.
22. Tillmann T, Vaucher J, Okbay A, et al. Education and coronary heart disease: mendelian randomisation study. *BMJ (Clinical research ed).* 2017;358:j3542-j3542.
23. Rosoff DB, Kaminsky ZA, Lohoff FW. Educational attainment reduces the risk of suicide attempt among individuals with and without psychiatric disorders independent of cognition: a multivariable Mendelian randomization study with more than 815,000 participants. *medRxiv.* 2019:2019.2012.2014.19014787.
24. Lee JS, Mukhopadhyay P, Matyas C, et al. PCSK9 inhibition as a novel therapeutic target for alcoholic liver disease. *Scientific Reports.* 2019;9(1):17167.
25. Rosoff DB, Charlet K, Jung J, et al. Association of High-Intensity Binge Drinking With Lipid and Liver Function Enzyme Levels. *JAMA Network Open.* 2019;2(6):e195844-e195844.
26. Richardson TG, Sanderson E, Palmer TM, et al. Evaluating the relationship between circulating lipoprotein lipids and apolipoproteins with risk of coronary heart disease: A multivariable Mendelian randomisation analysis. *PLOS Medicine.* 2020;17(3):e1003062.
27. Holmes MV, Richardson TG, Ference BA, Davies NM, Davey Smith G. Integrating genomics with biomarkers and therapeutic targets to invigorate cardiovascular drug development. *Nat Rev Cardiol.* 2021;18(6):435-453.
28. Stanley ER, Berg KL, Einstein DB, et al. Biology and action of colony--stimulating factor-1. *Mol Reprod Dev.* 1997;46(1):4-10.
29. Shaposhnik Z, Wang X, Lusis AJ. Arterial colony stimulating factor-1 influences atherosclerotic lesions by regulating monocyte migration and apoptosis. *J Lipid Res.* 2010;51(7):1962-1970.
30. Le Meur Y, Fixe P, Aldigier JC, Leroux-Robert C, Praloran V. Macrophage colony stimulating factor involvement in uremic patients. *Kidney Int.* 1996;50(3):1007-1012.
31. Masek-Hammerman K, Peeva E, Ahmad A, et al. Monoclonal antibody against macrophage colony-stimulating factor suppresses circulating monocytes and tissue macrophage function but does not alter cell infiltration/activation in cutaneous lesions or clinical outcomes in patients with cutaneous lupus erythematosus. *Clin Exp Immunol.* 2016;183(2):258-270.
32. Calvo A, Joensuu H, Sebastian M, et al. Phase Ib/II study of lacnotuzumab (MCS110) combined with spartalizumab (PDR001) in patients (pts) with advanced tumors. *Journal of Clinical Oncology.* 2018;36(15_suppl):3014-3014.
33. Pognan F, Couttet P, Demin I, et al. Colony-Stimulating Factor-1 Antibody Lacnotuzumab in a Phase 1 Healthy Volunteer Study and Mechanistic Investigation of

Safety Outcomes. *Journal of Pharmacology and Experimental Therapeutics.* 2019;369(3):428.
34. Vincenti MP, Brinckerhoff CE. Transcriptional regulation of collagenase (MMP-1, MMP-13) genes in arthritis: integration of complex signaling pathways for the recruitment of gene-specific transcription factors. *Arthritis Research & Therapy.* 2002;4(3):157.
35. Cabral-Pacheco GA, Garza-Veloz I, Castruita-De la Rosa C, et al. The Roles of Matrix Metalloproteinases and Their Inhibitors in Human Diseases. *Int J Mol Sci.* 2020;21(24):9739.
36. Rabkin SW. The Role Matrix Metalloproteinases in the Production of Aortic Aneurysm. *Prog Mol Biol Transl Sci.* 2017;147:239-265.
37. Winer A, Adams S, Mignatti P. Matrix Metalloproteinase Inhibitors in Cancer Therapy: Turning Past Failures Into Future Successes. *Mol Cancer Ther.* 2018;17(6):1147-1155.
38. Levin M, Udi Y, Solomonov I, Sagi I. Next generation matrix metalloproteinase inhibitors — Novel strategies bring new prospects. *Biochimica et Biophysica Acta (BBA) - Molecular Cell Research.* 2017;1864(11, Part A):1927-1939.
39. Rose-John S. Interleukin-6 signalling in health and disease. *F1000Res.* 2020;9:F1000 Faculty Rev-1013.
40. Tanaka T, Narazaki M, Kishimoto T. IL-6 in inflammation, immunity, and disease. *Cold Spring Harb Perspect Biol.* 2014;6(10):a016295-a016295.
41. Grotzinger AD, Rhemtulla M, de Vlaming R, et al. Genomic structural equation modelling provides insights into the multivariate genetic architecture of complex traits. *Nature Human Behaviour.* 2019;3(5):513-525.
42. Cheung MW. metaSEM: an R package for meta-analysis using structural equation modeling. *Front Psychol.* 2014;5:1521.
43. Savalei V, Bentler PM. A Two-Stage Approach to Missing Data: Theory and Application to Auxiliary Variables. *Structural Equation Modeling: A Multidisciplinary Journal.* 2009;16(3):477-497.
44. Yuan K-H, Bentler PM. Robust mean and covariance structure analysis through iteratively reweighted least squares. *Psychometrika.* 2000;65(1):43-58.
45. Turley P, Walters RK, Maghzian O, et al. Multi-trait analysis of genome-wide association summary statistics using MTAG. *Nature Genetics.* 2018;50(2):229-237.
46. Bulik-Sullivan BK, Loh P-R, Finucane HK, et al. LD Score regression distinguishes confounding from polygenicity in genome-wide association studies. *Nature Genetics.* 2015;47(3):291-295.
47. Karlsson Linnér R, Mallard TT, Barr PB, et al. Multivariate analysis of 1.5 million people identifies genetic associations with traits related to self-regulation and addiction. *Nat Neurosci.* 2021;24(10):1367-1376.
48. Mallard TT, Savage JE, Johnson EC, et al. Item-Level Genome-Wide Association Study of the Alcohol Use Disorders Identification Test in Three Population-Based Cohorts. *American Journal of Psychiatry.* 2021:appi.ajp.2020.20091390.
49. Hu Lt, Bentler PM. Cutoff criteria for fit indexes in covariance structure analysis: Conventional criteria versus new alternatives. *Structural Equation Modeling: A Multidisciplinary Journal.* 1999;6(1):1-55.

50. Karlsson Linnér R, Mallard TT, Barr PB, et al. Multivariate analysis of 1.5 million people identifies genetic associations with traits related to self-regulation and addiction. *Nature Neuroscience.* 2021;24(10):1367-1376.
51. Tejedor MT, Garcia-Sobreviela MP, Ledesma M, Arbones-Mainar JM. The apolipoprotein E polymorphism rs7412 associates with body fatness independently of plasma lipids in middle aged men. *PLoS One.* 2014;9(9):e108605.
52. Bekris LM, Millard SP, Galloway NM, et al. Multiple SNPs within and surrounding the apolipoprotein E gene influence cerebrospinal fluid apolipoprotein E protein levels. *J Alzheimers Dis.* 2008;13(3):255-266.
53. Li Z, Shue F, Zhao N, Shinohara M, Bu G. APOE2: protective mechanism and therapeutic implications for Alzheimer's disease. *Molecular Neurodegeneration.* 2020;15(1):63.
54. United Nations DoEaSA, Population Division. World Populaiton Prospects 2022: Summary of Results. UN DESA/POP/2022/TR/NO. 3. Published 2022. Accessed.
55. Garmany A, Yamada S, Terzic A. Longevity leap: mind the healthspan gap. *npj Regenerative Medicine.* 2021;6(1):57.
56. Beard JR, Officer A, de Carvalho IA, et al. The World report on ageing and health: a policy framework for healthy ageing. *Lancet.* 2016;387(10033):2145-2154.
57. Olshansky SJ. From Lifespan to Healthspan. *Jama.* 2018;320(13):1323-1324.
58. Wang G, Sarkar A, Carbonetto P, Stephens M. A simple new approach to variable selection in regression, with application to genetic fine mapping. *Journal of the Royal Statistical Society: Series B (Statistical Methodology).* 2020;82(5):1273-1300.
59. Zou Y, Carbonetto P, Wang G, Stephens M. Fine-mapping from summary data with the "Sum of Single Effects" model. *PLOS Genetics.* 2022;18(7):e1010299.
60. Benner C, Spencer CCA, Havulinna AS, Salomaa V, Ripatti S, Pirinen M. FINEMAP: efficient variable selection using summary data from genome-wide association studies. *Bioinformatics.* 2016;32(10):1493-1501.
61. Schilder BM, Humphrey J, Raj T. echolocatoR: an automated end-to-end statistical and functional genomic fine-mapping pipeline. *Bioinformatics.* 2022;38(2):536-539.
62. Hutchinson A, Asimit J, Wallace C. Fine-mapping genetic associations. *Hum Mol Genet.* 2020;29(R1):R81-r88.
63. Feng H, Mancuso N, Gusev A, et al. Leveraging expression from multiple tissues using sparse canonical correlation analysis and aggregate tests improve the power of transcriptome-wide association studies. *bioRxiv.* 2020:2020.2007.2003.186247.
64. Feng H, Mancuso N, Gusev A, et al. Leveraging expression from multiple tissues using sparse canonical correlation analysis and aggregate tests improves the power of transcriptome-wide association studies. *PLOS Genetics.* 2021;17(4):e1008973.
65. Giambartolomei C, Vukcevic D, Schadt EE, et al. Bayesian test for colocalisation between pairs of genetic association studies using summary statistics. *PLoS Genet.* 2014;10(5):e1004383.
66. Mancuso N, Freund MK, Johnson R, et al. Probabilistic fine-mapping of transcriptome-wide association studies. *Nature Genetics.* 2019;51(4):675-682.
67. Dall'Aglio L, Lewis CM, Pain O. Delineating the Genetic Component of Gene Expression in Major Depression. *Biological Psychiatry.* 2021;89(6):627-636.
68. de Leeuw CA, Mooij JM, Heskes T, Posthuma D. MAGMA: Generalized Gene-Set Analysis of GWAS Data. *PLOS Computational Biology.* 2015;11(4):e1004219.

69. Watanabe K, Taskesen E, van Bochoven A, Posthuma D. Functional mapping and annotation of genetic associations with FUMA. *Nature Communications.* 2017;8(1):1826.
70. Sobczyk MK, Gaunt TR, Paternoster L. MendelVar: gene prioritization at GWAS loci using phenotypic enrichment of Mendelian disease genes. *Bioinformatics.* 2021.
71. Schriml LM, Munro JB, Schor M, et al. The Human Disease Ontology 2022 update. *Nucleic Acids Res.* 2022;50(D1):D1255-d1261.
72. Köhler S, Gargano M, Matentzoglu N, et al. The Human Phenotype Ontology in 2021. *Nucleic Acids Res.* 2021;49(D1):D1207-d1217.
73. Davey Smith G, Hemani G. Mendelian randomization: genetic anchors for causal inference in epidemiological studies. *Human molecular genetics.* 2014;23(R1):R89-R98.
74. Smith GD. Use of genetic markers and gene-diet interactions for interrogating population-level causal influences of diet on health. *Genes & nutrition.* 2011;6(1):27-43.
75. Smith GD, Ebrahim S. 'Mendelian randomization': can genetic epidemiology contribute to understanding environmental determinants of disease? *Int J Epidemiol.* 2003;32(1):1-22.
76. Davies NM, Holmes MV, Smith GD. Reading Mendelian randomisation studies: a guide, glossary, and checklist for clinicians. *Bmj.* 2018;362.
77. Bosnes I, Nordahl HM, Stordal E, Bosnes O, Myklebust T, Almkvist O. Lifestyle predictors of successful aging: A 20-year prospective HUNT study. *PLoS One.* 2019;14(7):e0219200.
78. Hartmann A, Hartmann C, Secci R, Hermann A, Fuellen G, Walter M. Ranking Biomarkers of Aging by Citation Profiling and Effort Scoring. *Frontiers in Genetics.* 2021;12.
79. Zenin A, Tsepilov Y, Sharapov S, et al. Identification of 12 genetic loci associated with human healthspan. *Communications Biology.* 2019;2(1):41.
80. Searle SD, Mitnitski A, Gahbauer EA, Gill TM, Rockwood K. A standard procedure for creating a frailty index. *BMC Geriatrics.* 2008;8(1):24.
81. Atkins JL, Jylhävä J, Pedersen NL, et al. A genome-wide association study of the frailty index highlights brain pathways in ageing. *Aging Cell.* 2021;20(9):e13459.
82. Robinson SM, Jameson KA, Syddall HE, Dennison EM, Cooper C, Aihie Sayer A. Clustering of lifestyle risk factors and poor physical function in older adults: the Hertfordshire cohort study. *J Am Geriatr Soc.* 2013;61(10):1684-1691.
83. Krokstad S, Ding D, Grunseit AC, et al. Multiple lifestyle behaviours and mortality, findings from a large population-based Norwegian cohort study - The HUNT Study. *BMC Public Health.* 2017;17(1):58.
84. Lafortune L, Martin S, Kelly S, et al. Behavioural Risk Factors in Mid-Life Associated with Successful Ageing, Disability, Dementia and Frailty in Later Life: A Rapid Systematic Review. *PLoS One.* 2016;11(2):e0144405.
85. Rico-Uribe LA, Caballero FF, Martín-María N, Cabello M, Ayuso-Mateos JL, Miret M. Association of loneliness with all-cause mortality: A meta-analysis. *PLoS One.* 2018;13(1):e0190033.
86. Vila J. Social Support and Longevity: Meta-Analysis-Based Evidence and Psychobiological Mechanisms. *Frontiers in Psychology.* 2021;12.
87. Steptoe A, Shankar A, Demakakos P, Wardle J. Social isolation, loneliness, and all-cause mortality in older men and women. *Proceedings of the National Academy of Sciences.* 2013;110(15):5797-5801.

88. Nelson PG, Promislow DEL, Masel J. Biomarkers for Aging Identified in Cross-sectional Studies Tend to Be Non-causative. *J Gerontol A Biol Sci Med Sci.* 2020;75(3):466-472.
89. Mutlu AS, Duffy J, Wang MC. Lipid metabolism and lipid signals in aging and longevity. *Dev Cell.* 2021;56(10):1394-1407.
90. Yourman LC, Cenzer IS, Boscardin WJ, et al. Evaluation of Time to Benefit of Statins for the Primary Prevention of Cardiovascular Events in Adults Aged 50 to 75 Years: A Meta-analysis. *JAMA Intern Med.* 2021;181(2):179-185.
91. Brewer RA, Gibbs VK, Smith DL, Jr. Targeting glucose metabolism for healthy aging. *Nutr Healthy Aging.* 2016;4(1):31-46.
92. Barzilai N, Huffman DM, Muzumdar RH, Bartke A. The critical role of metabolic pathways in aging. *Diabetes.* 2012;61(6):1315-1322.
93. Miura K, Daviglus ML, Dyer AR, et al. Relationship of Blood Pressure to 25-Year Mortality Due to Coronary Heart Disease, Cardiovascular Diseases, and All Causes in Young Adult Men: The Chicago Heart Association Detection Project in Industry. *Archives of Internal Medicine.* 2001;161(12):1501-1508.
94. Rea F, Cantarutti A, Merlino L, Ungar A, Corrao G, Mancia G. Antihypertensive Treatment in Elderly Frail Patients. *Hypertension.* 2020;76(2):442-449.
95. Vaduganathan M, Claggett BL, Juraschek SP, Solomon SD. Assessment of Long-term Benefit of Intensive Blood Pressure Control on Residual Life Span: Secondary Analysis of the Systolic Blood Pressure Intervention Trial (SPRINT). *JAMA Cardiology.* 2020;5(5):576-581.
96. Larbi A, Franceschi C, Mazzatti D, Solana R, Wikby A, Pawelec G. Aging of the immune system as a prognostic factor for human longevity. *Physiology (Bethesda).* 2008;23:64-74.
97. Franceschi C, Bonafè M, Valensin S, et al. Inflamm-aging. An evolutionary perspective on immunosenescence. *Ann N Y Acad Sci.* 2000;908:244-254.
98. Hurme M, Kivimäki M, Pertovaara M, et al. CRP gene is involved in the regulation of human longevity: A follow-up study in Finnish nonagenarians. *Mechanisms of Ageing and Development.* 2007;128(10):574-576.
99. Oh TK, Jang ES, Song I-A. Long-term mortality due to infection associated with elevated liver enzymes: a population-based cohort study. *Scientific Reports.* 2021;11(1):12490.
100. Sohn J, Kang DR, Kim HC, et al. Elevation of Serum Aminotransferase Levels and Future Risk of Death from External Causes: A Prospective Cohort Study in Korea. *Yonsei Med J.* 2015;56(6):1582-1589.
101. Ori A, Toyama Brandon H, Harris Michael S, et al. Integrated Transcriptome and Proteome Analyses Reveal Organ-Specific Proteome Deterioration in Old Rats. *Cell Systems.* 2015;1(3):224-237.
102. Liu Y, Basty N, Whitcher B, et al. Genetic architecture of 11 organ traits derived from abdominal MRI using deep learning. *Elife.* 2021;10.
103. Mangan D. Iron: an underrated factor in aging. *Aging (Albany NY).* 2021;13(19):23407-23415.
104. Timmers PRHJ, Wilson JF, Joshi PK, Deelen J. Multivariate genomic scan implicates novel loci and haem metabolism in human ageing. *Nature Communications.* 2020;11(1):3570.
105. Hemani G, Tilling K, Smith GD. Orienting the causal relationship between imprecisely measured traits using GWAS summary data. *Plos Genetics.* 2017;13(11).

106. Burgess S, Thompson SG. Avoiding bias from weak instruments in Mendelian randomization studies. *Int J Epidemiol.* 2011;40(3):755-764.
107. Hemani G, Zheng J, Elsworth B, et al. The MR-Base platform supports systematic causal inference across the human phenome. *Elife.* 2018;7.
108. Bowden J, Del Greco MF, Minelli C, et al. Improving the accuracy of two-sample summary-data Mendelian randomization: moving beyond the NOME assumption. *Int J Epidemiol.* 2019;48(3):728-742.
109. Bowden J, Del Greco MF, Minelli C, Smith GD, Sheehan N, Thompson J. A framework for the investigation of pleiotropy in two-sample summary data Mendelian randomization. *Statistics in Medicine.* 2017;36(11):1783-1802.
110. Yavorska OO, Burgess S. MendelianRandomization: an R package for performing Mendelian randomization analyses using summarized data. *Int J Epidemiol.* 2017;46(6):1734-1739.
111. Rees JMB, Wood AM, Burgess S. Extending the MR-Egger method for multivariable Mendelian randomization to correct for both measured and unmeasured pleiotropy. *Stat Med.* 2017;36(29):4705-4718.
112. Yavorska OO, Burgess S. MendelianRandomization: an R package for performing Mendelian randomization analyses using summarized data. *International journal of epidemiology.* 2017;46(6):1734-1739.
113. Skrivankova VW, Richmond RC, Woolf BAR, et al. Strengthening the reporting of observational studies in epidemiology using mendelian randomisation (STROBE-MR): explanation and elaboration. *BMJ.* 2021;375:n2233.
114. Burgess S, Davies NM, Thompson SG. Bias due to participant overlap in two-sample Mendelian randomization. *Genet Epidemiol.* 2016;40(7):597-608.
115. Mounier N, Kutalik Z. Bias correction for inverse variance weighting Mendelian randomization. *bioRxiv.* 2022:2021.2003.2026.437168.
116. Minelli C, Del Greco MF, van der Plaat DA, Bowden J, Sheehan NA, Thompson J. The use of two-sample methods for Mendelian randomization analyses on single large datasets. *Int J Epidemiol.* 2021;50(5):1651-1659.
117. Neale-Lab. UK Biobank GWAS. http://www.nealelab.is/uk-biobank/. Published 2018. Accessed June 2019.
118. Chaudhury A, Duvoor C, Reddy Dendi VS, et al. Clinical Review of Antidiabetic Drugs: Implications for Type 2 Diabetes Mellitus Management. *Front Endocrinol (Lausanne).* 2017;8:6.
119. Mendez D, Gaulton A, Bento AP, et al. ChEMBL: towards direct deposition of bioassay data. *Nucleic Acids Research.* 2019;47(D1):D930-D940.
120. Wishart DS, Knox C, Guo AC, et al. DrugBank: a comprehensive resource for in silico drug discovery and exploration. *Nucleic Acids Res.* 2006;34(Database issue):D668-672.
121. Ference BA, Kastelein JJP, Ray KK, et al. Association of Triglyceride-Lowering LPL Variants and LDL-C–Lowering LDLR Variants With Risk of Coronary Heart Disease. *JAMA.* 2019;321(4):364-373.
122. Bhatt-Wessel B, Jordan TW, Miller JH, Peng L. Role of DGAT enzymes in triacylglycerol metabolism. *Arch Biochem Biophys.* 2018;655:1-11.
123. Willer CJ, Schmidt EM, Sengupta S, et al. Discovery and refinement of loci associated with lipid levels. *Nat Genet.* 2013;45(11):1274-1283.

124. Suchard MA, Schuemie MJ, Krumholz HM, et al. Comprehensive comparative effectiveness and safety of first-line antihypertensive drug classes: a systematic, multinational, large-scale analysis. *The Lancet.* 2019;394(10211):1816-1826.
125. Gill D, Georgakis MK, Koskeridis F, et al. Use of Genetic Variants Related to Antihypertensive Drugs to Inform on Efficacy and Side Effects. *Circulation.* 2019;140(4):270-279.
126. Auton A, Abecasis GR, Altshuler DM, et al. A global reference for human genetic variation. *Nature.* 2015;526(7571):68-74.
127. Gordillo-Marañón M, Zwierzyna M, Charoen P, et al. Validation of lipid-related therapeutic targets for coronary heart disease prevention using human genetics. *Nature Communications.* 2021;12(1):6120.
128. Holmes MV, Richardson TG, Ference BA, Davies NM, Davey Smith G. Integrating genomics with biomarkers and therapeutic targets to invigorate cardiovascular drug development. *Nature Reviews Cardiology.* 2021;18(6):435-453.
129. McCorrison J, Girke T, Goetz LH, Miller RA, Schork NJ. Genetic Support for Longevity-Enhancing Drug Targets: Issues, Preliminary Data, and Future Directions. *J Gerontol A Biol Sci Med Sci.* 2019;74(Suppl_1):S61-s71.
130. Smedley D, Haider S, Ballester B, et al. BioMart – biological queries made easy. *BMC Genomics.* 2009;10(1):22.
131. Chen J, Spracklen CN, Marenne G, et al. The trans-ancestral genomic architecture of glycemic traits. *Nat Genet.* 2021;53(6):840-860.
132. Rosoff Daniel B, Bell Andrew S, Jung J, Wagner J, Mavromatis Lucas A, Lohoff Falk W. Mendelian Randomization Study of PCSK9 and HMG-CoA Reductase Inhibition and Cognitive Function. *Journal of the American College of Cardiology.* 2022;80(7):653-662.
133. Finan C, Gaulton A, Kruger FA, et al. The druggable genome and support for target identification and validation in drug development. *Sci Transl Med.* 2017;9(383).
134. Gaziano L, Giambartolomei C, Pereira AC, et al. Actionable druggable genome-wide Mendelian randomization identifies repurposing opportunities for COVID-19. *Nature Medicine.* 2021;27(4):668-676.
135. Kuhn M, von Mering C, Campillos M, Jensen LJ, Bork P. STITCH: interaction networks of chemicals and proteins. *Nucleic Acids Res.* 2008;36(Database issue):D684-688.
136. Griffith M, Griffith OL, Coffman AC, et al. DGIdb: mining the druggable genome. *Nat Methods.* 2013;10(12):1209-1210.
137. Folkersen L, Gustafsson S, Wang Q, et al. Genomic evaluation of circulating proteins for drug target characterisation and precision medicine. *bioRxiv.* 2020:2020.2004.2003.023804.
138. Sun BB, Maranville JC, Peters JE, et al. Genomic atlas of the human plasma proteome. *Nature.* 2018;558(7708):73-79.
139. Suhre K, Arnold M, Bhagwat AM, et al. Connecting genetic risk to disease end points through the human blood plasma proteome. *Nature Communications.* 2017;8(1):14357.
