# Supplementary material for: Multivariate genome-wide analysis of aging-related traits identifies novel loci and new drug targets for healthy aging
Source: Nat Aging. 2023 Aug 7;3(8):1020–35. doi: 10.1038/s43587-023-00455-5 (PMC10432278; doi:10.1038/s43587-023-00455-5)
Supplement: Supplementary file 2 — Reporting Summary [file 43587_2023_455_MOESM2_ESM.pdf]

nature portfolio

Corresponding author(s): Falk W. Lohoff

Last updated by author(s): May 15th, 2023

Reporting Summary

Nature Portfolio wishes to improve the reproducibility of the work that we publish. This form provides structure for consistency and transparency in reporting. For further information on Nature Portfolio policies, see our Editorial Policies and the Editorial Policy Checklist.

Statistics

For all statistical analyses, confirm that the following items are present in the figure legend, table legend, main text, or Methods section.

n/a	Confirmed	
☐	☒	The exact sample size (*n*) for each experimental group/condition, given as a discrete number and unit of measurement
☐	☒	A statement on whether measurements were taken from distinct samples or whether the same sample was measured repeatedly
☐	☒	The statistical test(s) used AND whether they are one- or two-sided<br>*Only common tests should be described solely by name; describe more complex techniques in the Methods section.*
☒	☐	A description of all covariates tested
☐	☒	A description of any assumptions or corrections, such as tests of normality and adjustment for multiple comparisons
☐	☒	A full description of the statistical parameters including central tendency (e.g. means) or other basic estimates (e.g. regression coefficient) AND variation (e.g. standard deviation) or associated estimates of uncertainty (e.g. confidence intervals)
☐	☒	For null hypothesis testing, the test statistic (e.g. *F*, *t*, *r*) with confidence intervals, effect sizes, degrees of freedom and *P* value noted<br>*Give P values as exact values whenever suitable.*
☒	☐	For Bayesian analysis, information on the choice of priors and Markov chain Monte Carlo settings
☒	☐	For hierarchical and complex designs, identification of the appropriate level for tests and full reporting of outcomes
☐	☒	Estimates of effect sizes (e.g. Cohen's *d*, Pearson's *r*), indicating how they were calculated

Our web collection on statistics for biologists contains articles on many of the points above.

Software and code

Policy information about availability of computer code

Data collection	No software was used to collect data for this study. All analyses in this study were conducted using publicly available data.
Data analysis	The software used in this study are available at the respective online repositories. R package GenomicSEM version 0.5.0: https://github.com/GenomicSEM/GenomicSEM; R package TwoSampleMR version 0.5.6: https://mrcieu.github.io/TwoSampleMR/; R package MendelianRandomization version 0.7.0: https://cran.r-project.org/web/packages/MendelianRandomization/index.html/; R package MRlap verion 0.0.3.0: https://github.com/n-mounier/MRlap; STITCH: http://stitch.embl.de/; R FUSION Pipeline version 1.4.2: http://gusevlab.org/projects/fusion/; Python package FOCUS version 0.6.10: http://github.com/bogdanlab/focus/; R package coloc version 5.1.0.1: https://cran.r-project.org/web/packages/coloc/index.html; Python package CELLECT version 1.3.0: https://github.com/perslab/CELLECT; Python package CELLEX version 1.2.1: https://github.com/perslab/CELLEX; R package echolocatoR version 2.0.3: https://github.com/RajLabMSSM/echolocatoR; MendelVar: https://mendelvar.mrcieu.ac.uk/; FUMA, MAGMA version 1.4.0: https://fuma.ctglab.nl/; R version 4.2.1 and Python version 3.8 were used to format data for analyses. Fig. 1 and Extended Data Figs. 5 and 7 were created using BioRender.com. Supplementary Figs. 1-21 was generated using FUMA version 1.4.0: https://fuma.ctglab.nl/; Supplementary Figs. 22-33 were generated using R package echolocatoR version 2.0.3: https://github.com/RajLabMSSM/echolocatoR.

For manuscripts utilizing custom algorithms or software that are central to the research but not yet described in published literature, software must be made available to editors and reviewers. We strongly encourage code deposition in a community repository (e.g. GitHub). See the Nature Portfolio guidelines for submitting code & software for further information.

Data

Policy information about availability of data

All manuscripts must include a data availability statement. This statement should provide the following information, where applicable:

- Accession codes, unique identifiers, or web links for publicly available datasets
- A description of any restrictions on data availability
- For clinical datasets or third party data, please ensure that the statement adheres to our policy

All analyses in this study were conducted using publicly available data. URLs for the source datasets are as follows: mvAge GWAS summary statistics: https://doi.org/10,5281/zenodo.7926323; longevity GWAS summary statistics: https://www.longevitygenomics.org/downloads; parental lifespan GWAS summary statistics: https://datashare.ed.ac.uk/handle/10283/3209; healthspan GWAS summary statistics: https://www.gwasarchive.org/; frailty index GWAS summary statistics: https://figshare.com/articles/dataset/Genome-Wide_Association_Study_of_the_Frailty_Index_-_Atkins_et_al_2019/9204998; epigenetic age acceleration, GWAS summary statistics: https://datashare.ed.ac.uk/handle/10283/3645; sCCA weights (used for transcriptomic imputation) and 1000 Genomes Project Phase 3 European genomic reference data (used for transcriptomic imputation and MR): http://gusevlab.org/projects/fusion/; biomarker and risk factor GWAS summary statistics used for MR: https://gwas.mrcieu.ac.uk/; scRNA-seq data used for cell-type enrichment analysis: https://tabula-muris.ds.czbiohub.org/; circulating protein levels from the SCALLOP Consortium: https://zenodo.org/record/2615265#.ZGEzyezMLN0. Any other data generated in this study upon which conclusions are based are available in the Supplementary Tables.

Human research participants

Policy information about studies involving human research participants and Sex and Gender in Research.

Reporting on sex and gender	All analyses in this study were conducted using publicly available genome-wide association study (GWAS) summary level statistics. Therefore, sex and gender were not considered in the design of this study. Also, sex-stratified GWASs of the aging-related traits incorporated in this study, i.e., longevity, lifespan, healthspan, frailty index, and epigenetic age acceleration, were not available, so disaggregated sex and gender multivariate GWASs of the shared aging factor could not be generated. The GWAS summary statistics for the five aging-related traits incorporated into the multivariate GWAS in this study were generated using additive models that accounted for covariates including self-reported sex. 1,560,432 participants were represented overall in the five aging-related GWASs, 51.8% of whom were female.
Population characteristics	This study uses only genome-wide association study (GWAS) summary level data (i.e., this study does not used individual-level data). See the GWAS links (provided in the manuscript and the Data section of this Reporting Summary) for information regarding population characteristics for the participating cohorts in the five aging-related GWASs incorporated into the multivariate GWAS generated in this study.
Recruitment	This study uses only genome-wide association study (GWAS) summary level data (i.e., this study does not used individual-level data). See the GWAS links (provided in the manuscript and the Data section of this Reporting Summary) for information regarding study recruitment for the participating cohorts in five aging-related GWASs incorporated into the multivariate GWAS generated in this study.
Ethics oversight	This study uses only publicly available genome-wide association study summary level data. The original GWAS studies each had approval from their respective review boards and ethics oversight authorities.

Note that full information on the approval of the study protocol must also be provided in the manuscript.

Field-specific reporting

Please select the one below that is the best fit for your research. If you are not sure, read the appropriate sections before making your selection.

☒ Life sciences ☐ Behavioural & social sciences ☐ Ecological, evolutionary & environmental sciences

For a reference copy of the document with all sections, see nature.com/documents/nr-reporting-summary-flat.pdf

Life sciences study design

All studies must disclose on these points even when the disclosure is negative.

Sample size	As statistical power for multivariate GWAS, transcriptomic imputation, polygenic Mendelian randomization, and drug-target Mendelian randomization analyses are dependent upon sample size, we maximized power of these analyses by including the largest GWAS available for each exposure and outcome.
Data exclusions	We used genetic variants associated with the exposures in these analyses (i.e., gene expression in the transcriptomic imputation and drug-target Mendelian randomization analyses, etc.). For the polygenic MR analyses of biomarkers and risk factors, we excluded genetic variants not strongly associated with their respective exposure (P-value > 5x10-8), or if they were in linkage disequilibrium with other strongly associated variants. We also performed extensive heterogeneity testing and follow up analyses excluding variants that demonstrated evidence for violating the MR assumptions #2 and #3. For the transcriptomic imputation and drug-target MR analyses, we used genetic

	variants located within or near the genomic loci of the genes included in the analyses. Genetic variants were conditionally independent and associated with the gene expression at pre-specified, analysis-specific P-value thresholds (i.e., default association criteria for the FUSION TWAS) and P-value < 5x10-8 for the MR analyses. For the other drug-target MR analyses of approved and proposed therapies as well as for the screen of protein-coding genes, we used standard MR genetic instrument selection criteria (P-value < 5x10-8) and selected genetic variants associated with each exposure irregardless of genomic position as is done with polygenic MR analysis.
Replication	We were unable to perform replication of the multivariate GWAS because independent GWAS summary level statistics for the five aging-related traits incorporated were not available. We performed extensive replication of the drug-target Mendelian randomization analyses using independent data sources (discussed in the Methods and Supplementary Methods)
Randomization	GWAS assess the associations of common variants with traits of interest. Fine-mapping, transcriptomic imputation and Mendelian randomization rely on genetic variation, which is randomized at conception for each study participant (i.e., genetic alleles are independently allocated at this time).
Blinding	This study does not used individual-level participant data (i.e., no raw data). Only publicly available summary statistics were used. Therefore, blinding is not applicable.

Reporting for specific materials, systems and methods

We require information from authors about some types of materials, experimental systems and methods used in many studies. Here, indicate whether each material, system or method listed is relevant to your study. If you are not sure if a list item applies to your research, read the appropriate section before selecting a response.

Materials & experimental systems

n/a	Involved in the study
☒	☐ Antibodies
☒	☐ Eukaryotic cell lines
☒	☐ Palaeontology and archaeology
☒	☐ Animals and other organisms
☒	☐ Clinical data
☒	☐ Dual use research of concern

Methods

n/a	Involved in the study
☒	☐ ChIP-seq
☒	☐ Flow cytometry
☒	☐ MRI-based neuroimaging
